# Author Correction: Reliability of high-quantity human brain organoids for modeling microcephaly, glioma invasion and drug screening

**DOI:** 10.1038/s41467-025-56677-1

**Published:** 2025-02-04

**Authors:** Anand Ramani, Giovanni Pasquini, Niklas J. Gerkau, Vaibhav Jadhav, Omkar Suhas Vinchure, Nazlican Altinisik, Hannes Windoffer, Sarah Muller, Ina Rothenaigner, Sean Lin, Aruljothi Mariappan, Dhanasekaran Rathinam, Ali Mirsaidi, Olivier Goureau, Lucia Ricci-Vitiani, Quintino Giorgio D’Alessandris, Bernd Wollnik, Alysson Muotri, Limor Freifeld, Nathalie Jurisch-Yaksi, Roberto Pallini, Christine R. Rose, Volker Busskamp, Elke Gabriel, Kamyar Hadian, Jay Gopalakrishnan

**Affiliations:** 1 Institute of Human Genetics, University Hospital, Friedrich-Schiller-Universität Jena, 07740 Jena, Germany; 2 Department of Ophthalmology, University Hospital Bonn, Medical Faculty, Bonn, Germany; 3 Institute of Neurobiology, Faculty of Mathematics and Natural Sciences, Heinrich-Heine-Universität, 40225 Düsseldorf, Germany; 4 Research Unit Signaling and Translation, Helmholtz Zentrum München, 85764 Neuherberg, Germany; 5 Kugelmeiers Ltd, Erlenbach, Switzerland; 6 Institut de la Vision, Sorbonne Université, INSERM, CNRS, F-75012 Paris, France; 7 Department of Oncology and Molecular Medicine, Istituto Superiore di Sanità, Viale Regina Elena 299, 00161 Rome, Italy; 8 Department of Neuroscience, Neurosurgery Section, Università Cattolica del Sacro Cuore, Rome, Italy; 9 Institute of Human Genetics, University Medical Center Göttingen, Göttingen, Germany; 10 University of California San Diego, School of Medicine, Department of Pediatrics/Rady Children’s Hospital-San Diego, San Diego, USA; 11 Department of Cellular & Molecular Medicine, Stem Cell Program, La Jolla, CA 92093 MC 0695 USA; 12 Department of Biomedical Engineering, Technion-Israel Institute of Technology, Haifa, Israel; 13 Department of Clinical and Molecular Medicine, Faculty of Medicine and Health Sciences, Norwegian University of Science and Technology, Trondheim, Norway; 14 Institute of Human Genetics, University Hospital, Heinrich-Heine-Universität, 40225 Düsseldorf, Germany

**Keywords:** Disease model, Developmental disorders, Stem-cell differentiation

Correction to: *Nature Communications* 10.1038/s41467-024-55226-6, published online 19 December 2024

The original version of this Article contained an error in Fig. 6Ai in which the image for the IMR90 Day 30 condition inadvertently used the image of the IMR90 Day 15 condition presented in Fig. 1G. Figure 6Ai has been updated with the correct image of IMR90 Day 30 condition. This error has been corrected in both the PDF and HTML versions of the Article.

In addition, the Supplementary Information file contained an error in Supplementary Fig. 6B in which the timepoints were incorrectly labelled in the order “Post thaw, Day5 - Post thaw, Day 30 - Post thaw, Day 15”. This has been updated with the correct order “Post thaw, Day 5 – Post thaw, Day 15 – Post thaw, Day 30”. Furthermore, the same figure panel contained an error in which the representative image for each timepoint of the “Non-frozen organoids” condition was inadvertently displayed as an incorrect timepoint: the image for “Post thaw, Day 5” was wrongly displayed as “Post thaw, Day 30”, the image for “Post thaw, Day 15” was wrongly displayed as “Post thaw, Day 5”, and the image for “Post thaw, Day 30” was wrongly displayed as “Post thaw, Day 15”. These errors have been corrected in the Supplementary Information PDF file.

