# Supplementary material for: Reliability of high-quantity human brain organoids for modeling microcephaly, glioma invasion and drug screening
Source: Nat Commun. 2024 Dec 19;15:10703. doi: 10.1038/s41467-024-55226-6 (PMC11659410; doi:10.1038/s41467-024-55226-6)
Supplement: Supplementary file 1 — Supplementary information [file 41467_2024_55226_MOESM1_ESM.pdf]

Supplementary information
Figure legend (Supplementary)

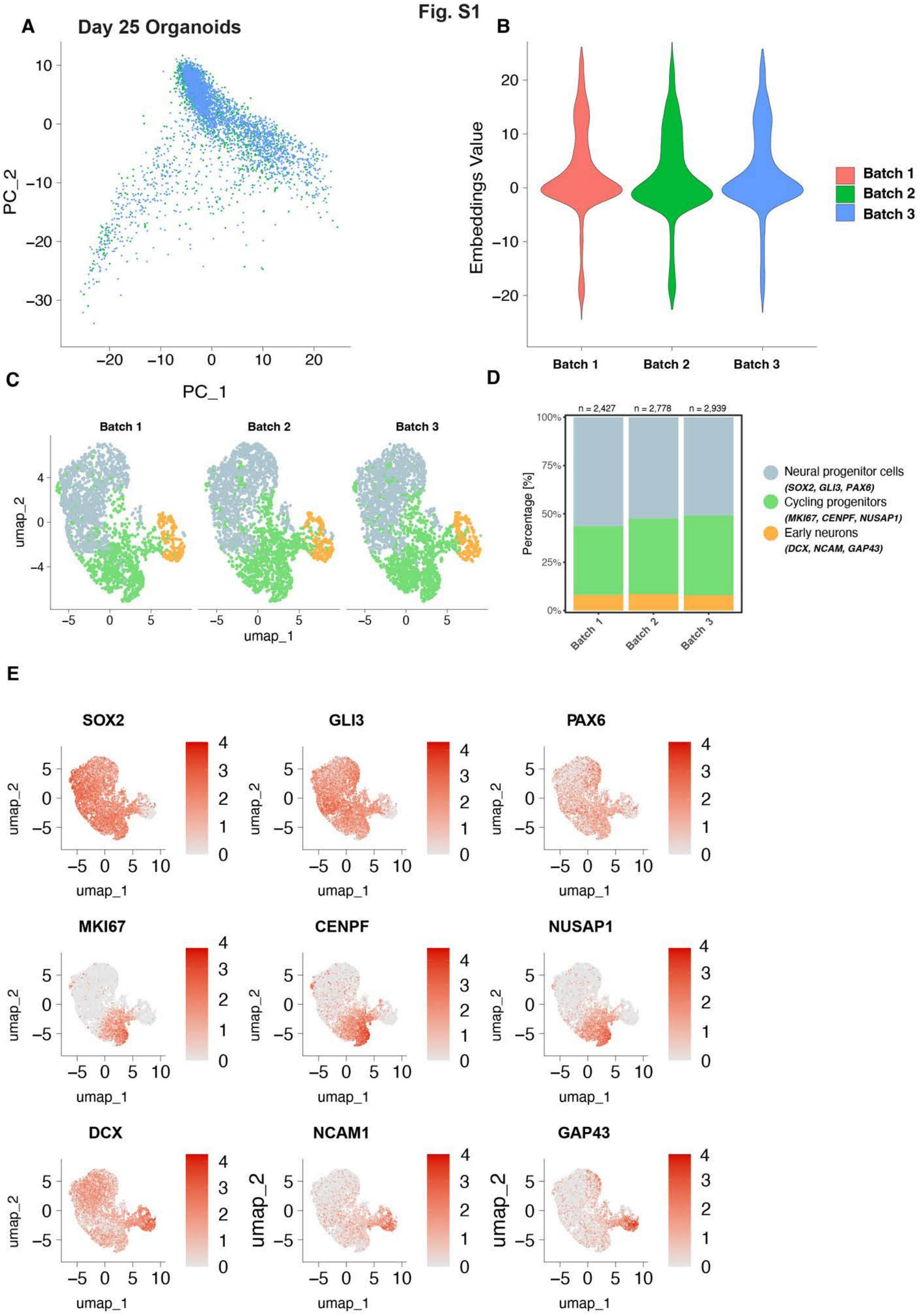

Figure S1: Testing batch-to-batch variations in Hi-Q brain organoids (Related to Figure 2).
A-B. Diagnostic **(A)** and a violin plot **(B)** of principal component (PC) analysis of sc-RNA transcriptomes from three independent batches of Hi-Q brain organoids representing cells. The batch effect is not apparent as all medians of samples are close to zero.

C. UMAP visualization and annotated cell types in three different batches.

D. Proportions of cells in each type showing no significant differences, confirming low batch-to-batch variation in terms of cell types and their proportions.

E. Feature plot of cells positive for individual marker genes used to identify and annotate cell types.

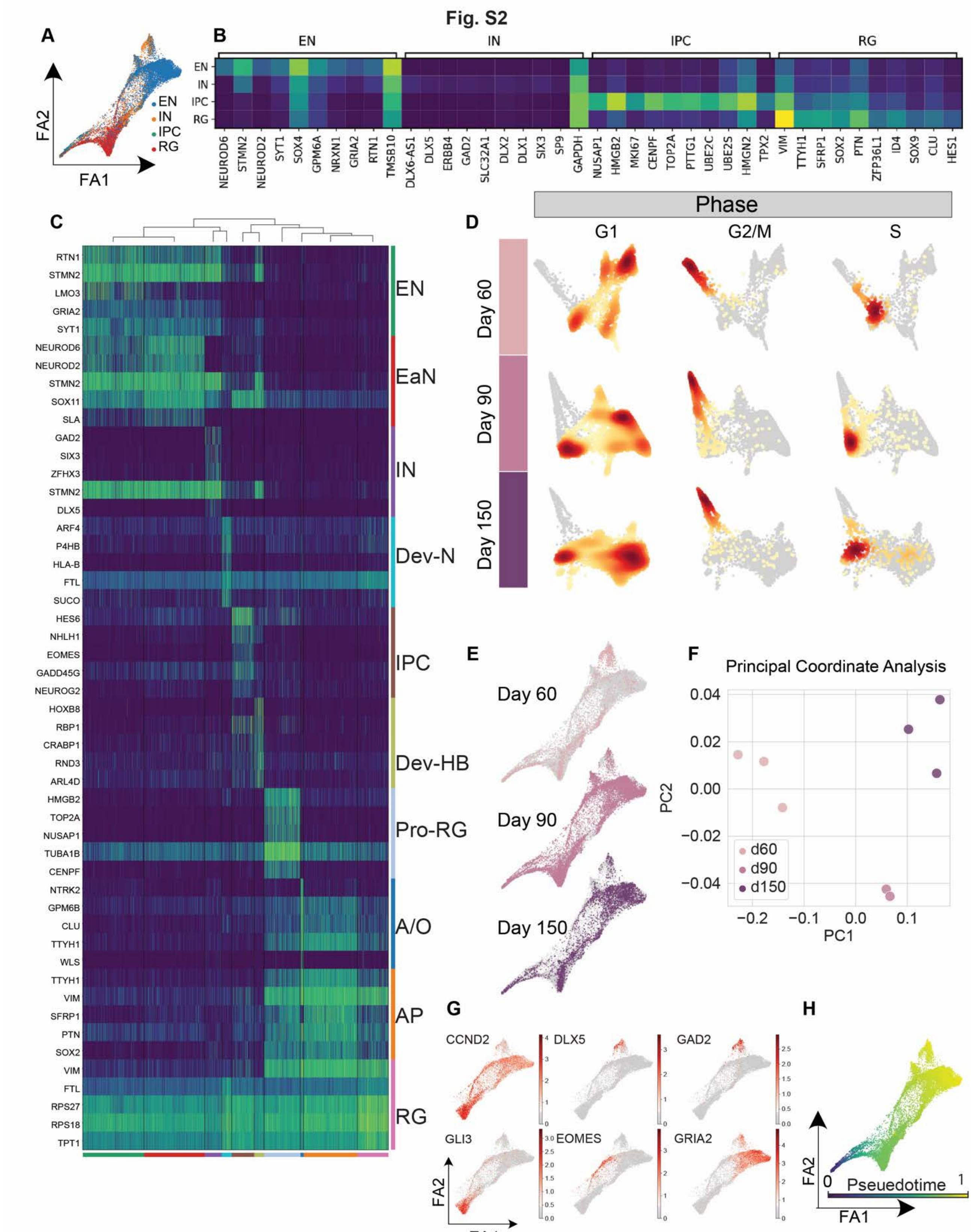

Figure S2. Cell type heterogeneity in Hi-Q brain organoids across different time points of maturation (Related to Figure 2).

A. Force Altas (FA) 2D representation of the neighbor graph of Hi-Q brain organoids from day 60, day 90, and day 150 datasets with cell type predicted labels.

B. Matrixplot showing the average gene expression of the marker genes (top 10) of each cell type label in the integrated dataset. Markers are calculated by pairwise Wilcoxon rank sum test of each group against the rest of the cells.

C. Heatmap showing the expression of marker genes calculated on unbiased Leiden clustering.

D. FA embedding of day 60, day 90, and day 150 separately indicates the density of cells expressing markers characteristic of cell-cycle stages.

E. FA embedding of the integrated datasets highlighting cells from different collection time points.

F. Scatterplot reporting each dataset in the function of the first two principal coordinates (PC) calculated from the proportion of cell types predicted in each batch of Hi-Q brain organoids.

G-H. FA embedding of the integrated dataset subset. Cells are highlighted by the expression of ventral telencephalon development markers (CCND2, DLX5, and GAD2) and dorsal telencephalon development markers (GLI3, EOMES, and GRIA2). **(H)** FA embedding of the whole integrated dataset is highlighted by the diffusion pseudotime score computed, setting the Pro-RG cluster as the root.

Fig. S3

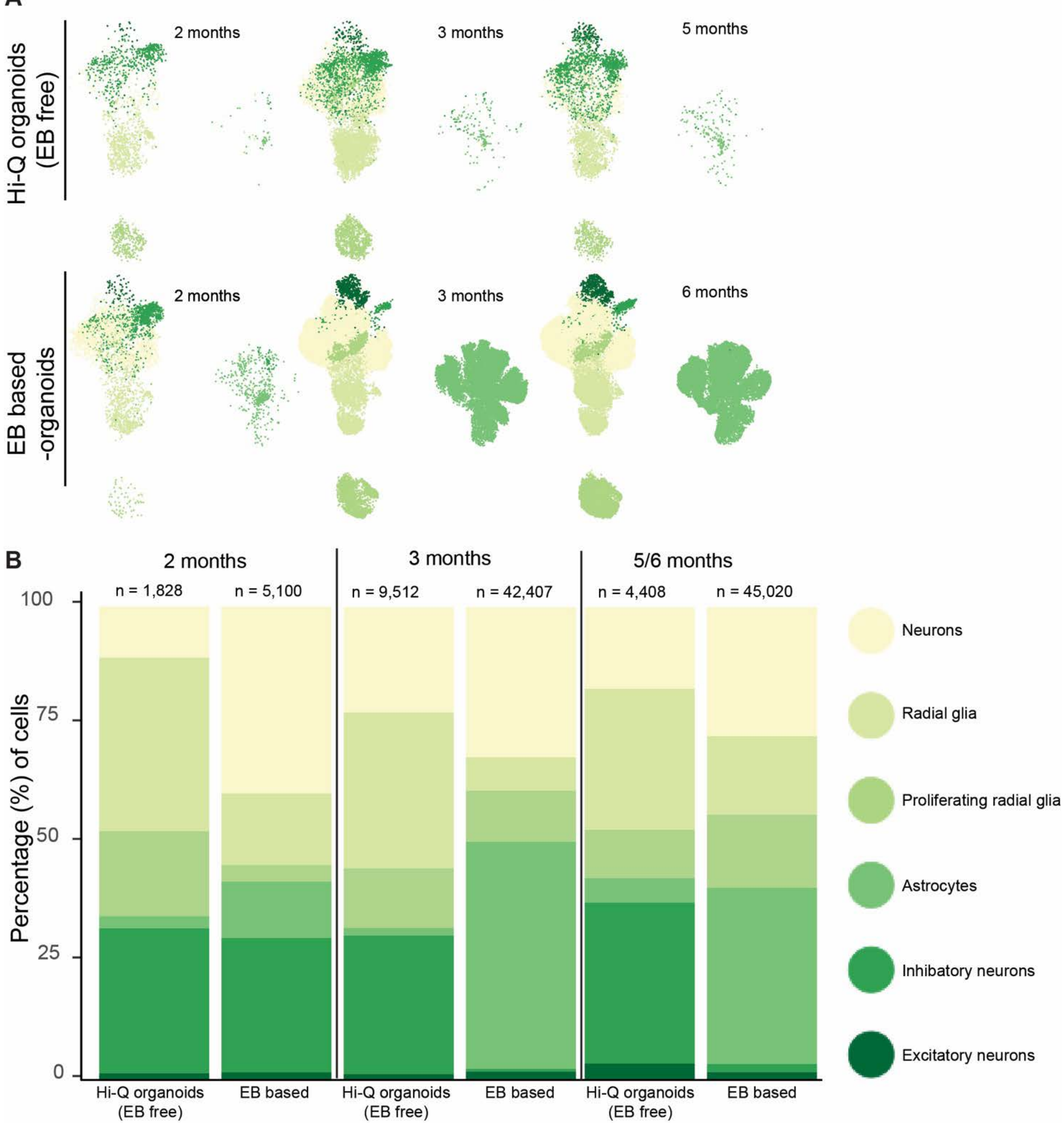

Figure S3. Cell type heterogeneity in Hi-Q brain organoids compare to EB-based brain organoids.

A. UMAP projection of the clusters representing assigned cell types across different types of age-matched organoids.

B. Bar plot depicting the proportions of assigned cell types in each organoid dataset, representing cell type heterogeneity.

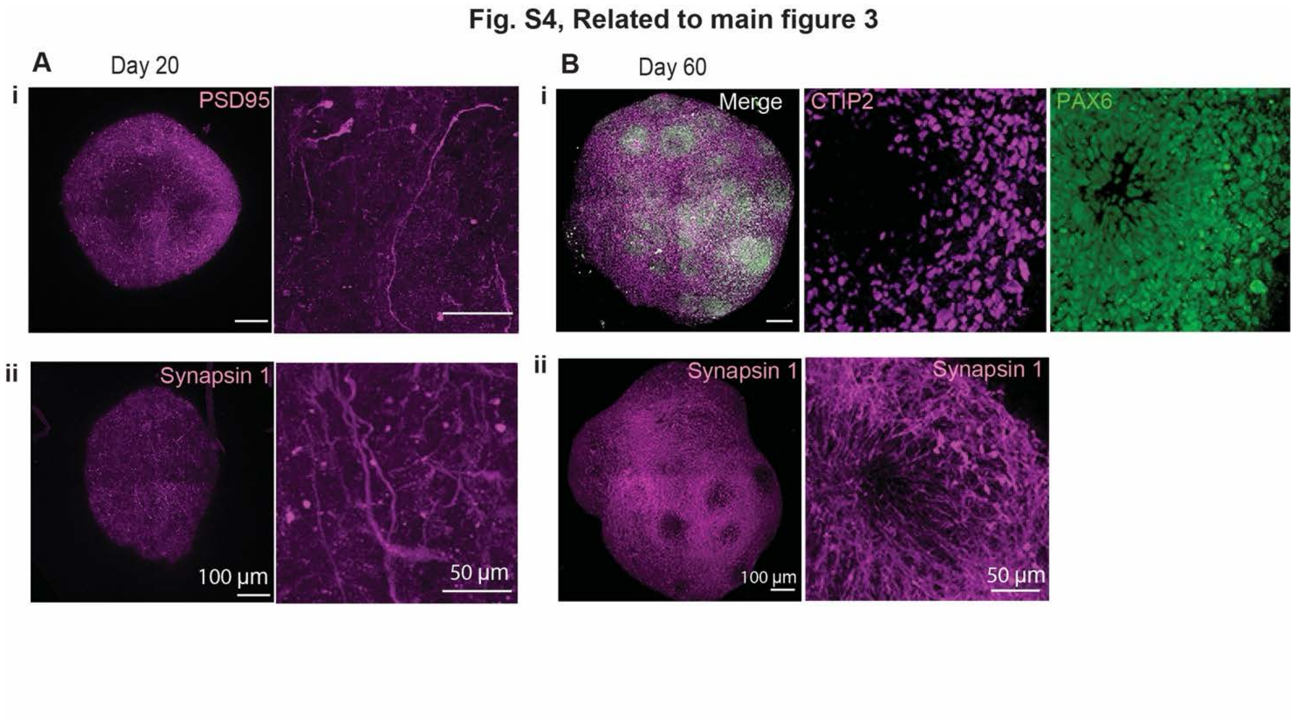

Figure S4: Hi-Q brain organoids mature over time (Related to Figure 3)
A-B. Tissue clearing and wholemount staining of day 20 **(Ai-ii)** and 60-day **(Bi-ii)** old Hi-Q brain organoids show the presence of PSD95 (magenta), CTIP2 (magenta), Synapsin 1(magenta), and PAX6 (green). Representative images are shown, and panels show scale bars.

Fig. S5, Related to main figure 3

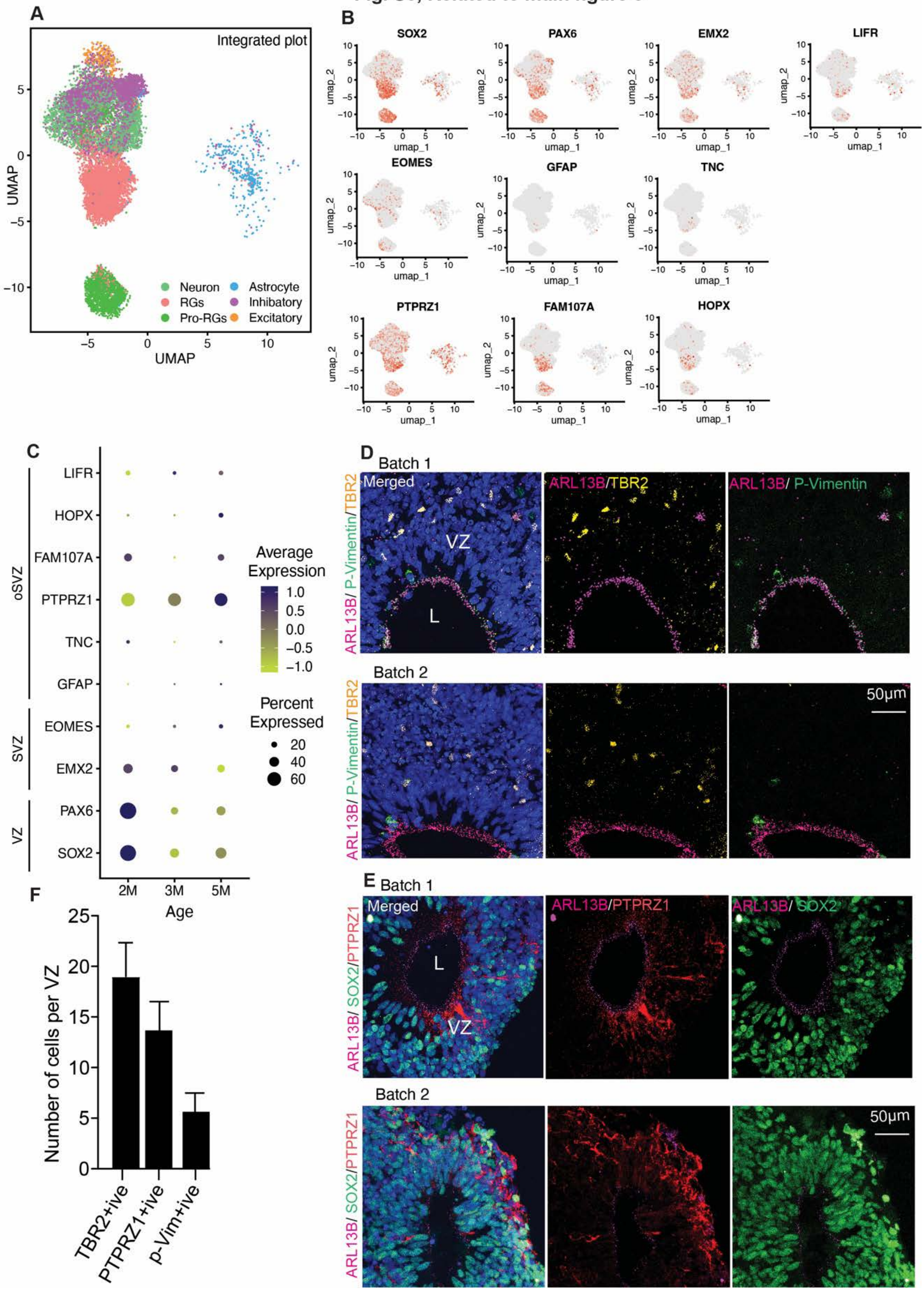

Figure S5: Layer identities in the ventricular zones of Hi-Q brain organoids.

A. UMAP visualization single-cell data showing six significant types of cell clusters (Color-coded).

B. Feature plots of annotated cell types positive for individual markers of VZ, SVZ, and oSVZ.

C. Dot plots displaying the marker expression levels across various age groups.

D. Immunofluorescence validation for the presence of various markers in organoid thin sections. In all cases, ARL13B (Magenta) labels primary cilia at the VZ lumen (L) at the apical side. TBR2

(Yellow) marks the distribution of intermediate progenitors, specifying the presence of sVZ, and p-Vimentin (green) marks outer radial glial cells that are basal to the VZ. P-Vimentin data in this figure should be combined with **Figure 6Ei**. Two representative illustrations from at least two independent batches of 60-day-old Hi-Q brain organoids. Scale bar 50 µm.

E. Immunofluorescence validation for the presence of PTPRZ1 (Red) specifying oSVZ. ARL13B (Magenta) labels primary cilia at the apical side of the VZ lumen (L). SOX2 (Green) marks the distribution of VZ progenitors. Two representative figures are given from at least two independent batches of organoids—scale bar 50 µm.

F. The bar diagram below shows the average number of each cell type quantified from at least six organoids (n=6) from two independent batches.

Fig. S6 (related to main figure 5)

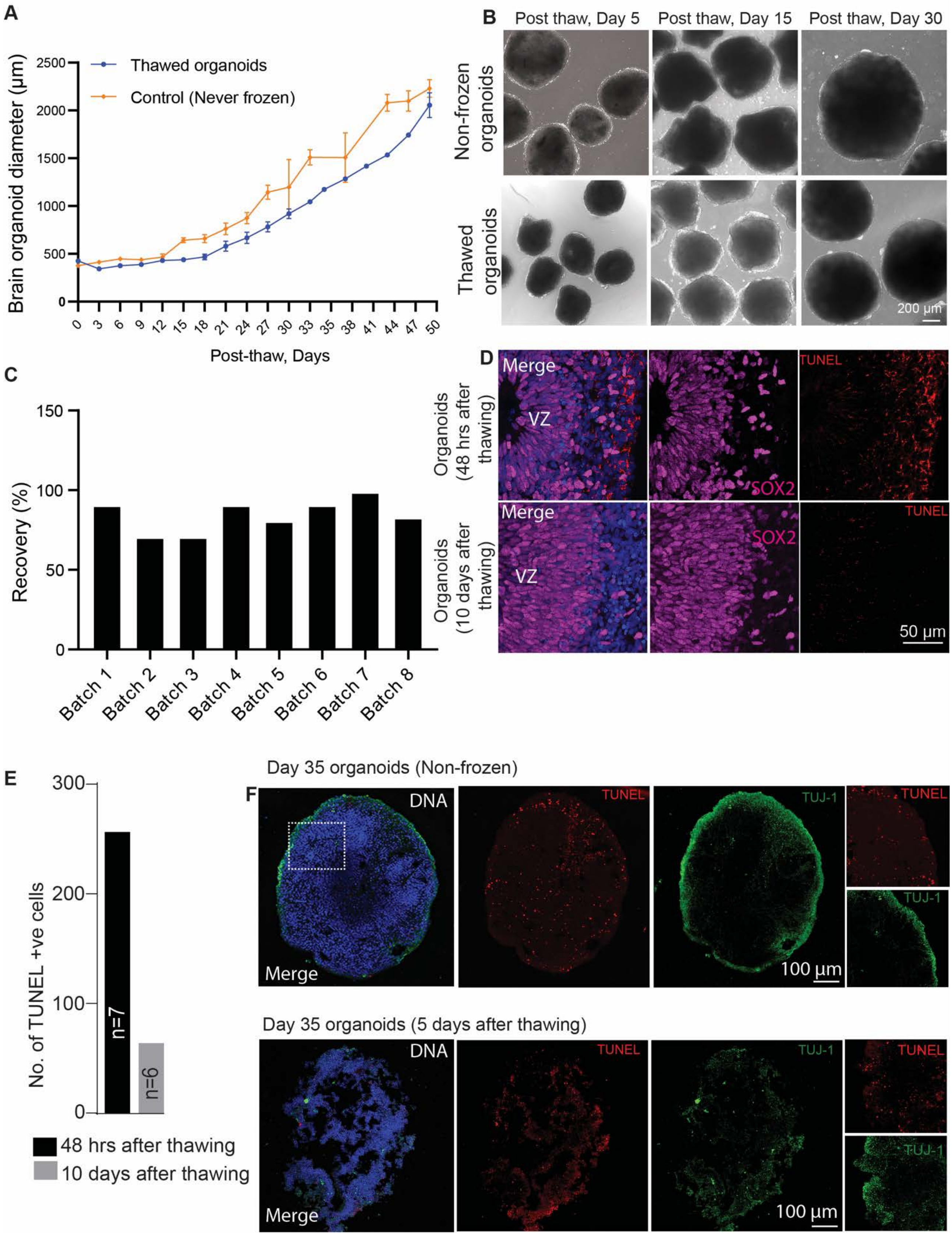

Figure S6: Cryopreservation, thawing, and re-culturing of Hi-Q brain organoids (Related to Figure 5)

A. Growth kinetics of thawed Hi-Q brain organoids (blue line) compared to control organoids (orange line) that have never been frozen. Each time point shows the average diameter of at least four organoids.

B. Macroscopical images of thawed Hi-Q organoids at various time points compared to control organoids that have never been frozen. Scale bar 200 µm shown in the panel.

C. Percentage recovery of thawed brain organoids from at least eight batches. Each batch contained at least ten brain organoids.

D. Cytoarchitectural analysis of thawed organoids after 48 hrs (top panel) and 10 days (bottom panel). SOX2 (magenta) specifies developing VZ, and TUNEL labels dead cells (red). The panel shows a scale bar.

E. Bar diagram counts TUNEL-positive cells between organoids 48 hrs and ten days after thawing of Hi-Q organoids. The number of organoids used in each experiment is denoted by "n."

F. Unlike Day 18 Hi-Q organoids, thawed Day 35 organoids did not display an intact cytoarchitectural organization. In addition, thawed organoids contained massively elevated TUNEL-positive cells. TUNEL (red) labels dead cells. TUJ1 (green) labels neurons. The panel shows a scale bar.

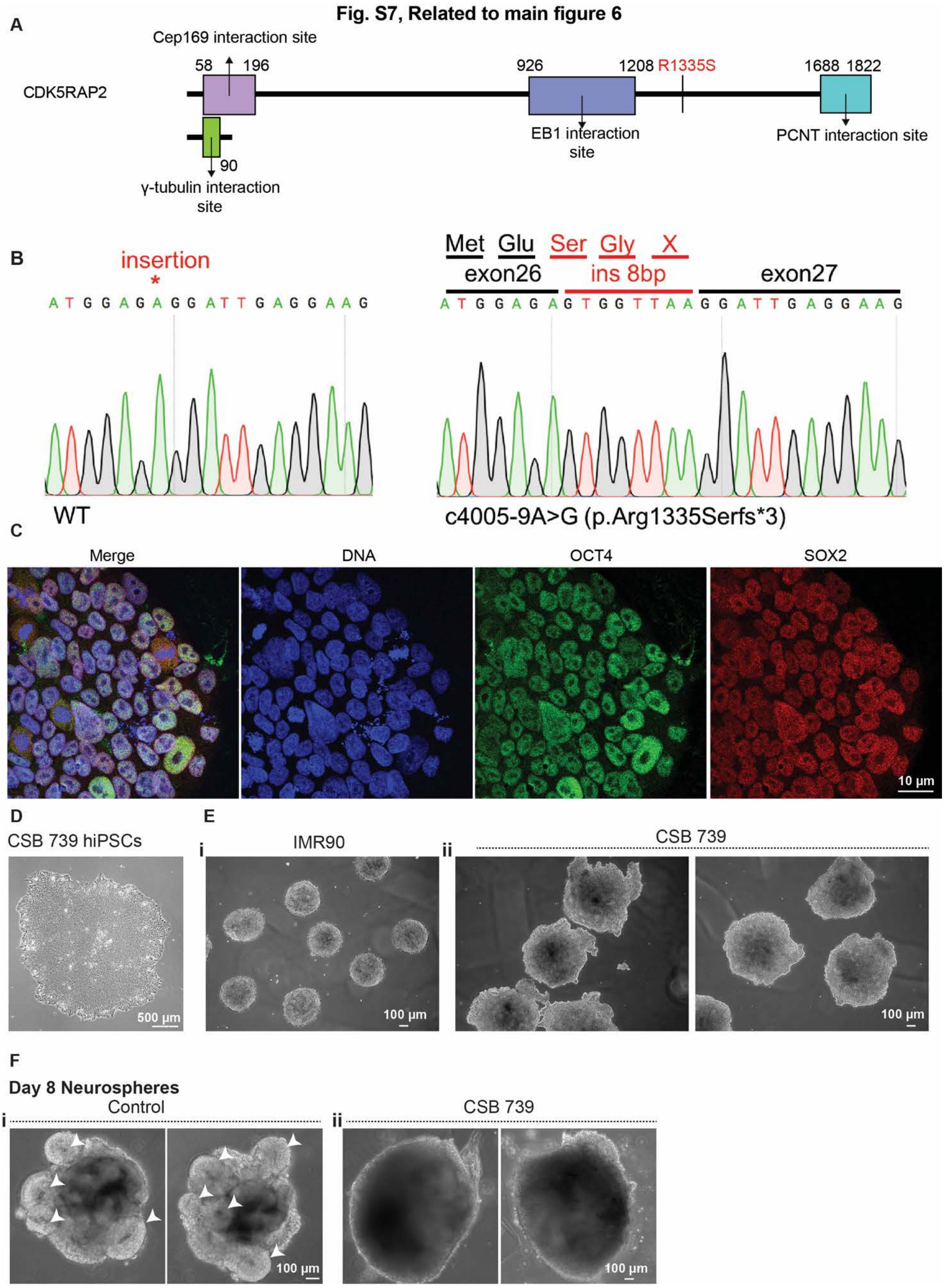

Figure S7: Hi-Q brain organoids model microcephaly caused by a CDK5RAP2 mutation and brain organization defects caused by a CSB mutation (Related to Figure 6)

A. Schematics of the CDK5RAP2 secondary structure showing protein interaction sites and the frameshift mutation R1335S.

B. DNA sequence of mutant CDK5RAP2 from patient-derived hiPSC (right) compared to healthy control (left). Nucleotide insertions between exon 26 and 27 are highlighted (red), leading to a frameshift with an amino acid change from R to S.

C. Patient-derived hiPSC displaying pluripotent markers of OCT4 and SOX2.

D-F. Comparison of neurospheres derived from healthy control (IMR90) and Cockayne Syndrome (CSB 739; due to a mutation is CSB). Bright field images. Arrowheads in panel F point to well-defined neurospheres in healthy controls. Such structures are not observed in CSB739. Panels show the scale bar.

Fig. S8 GBM screening, second part 1

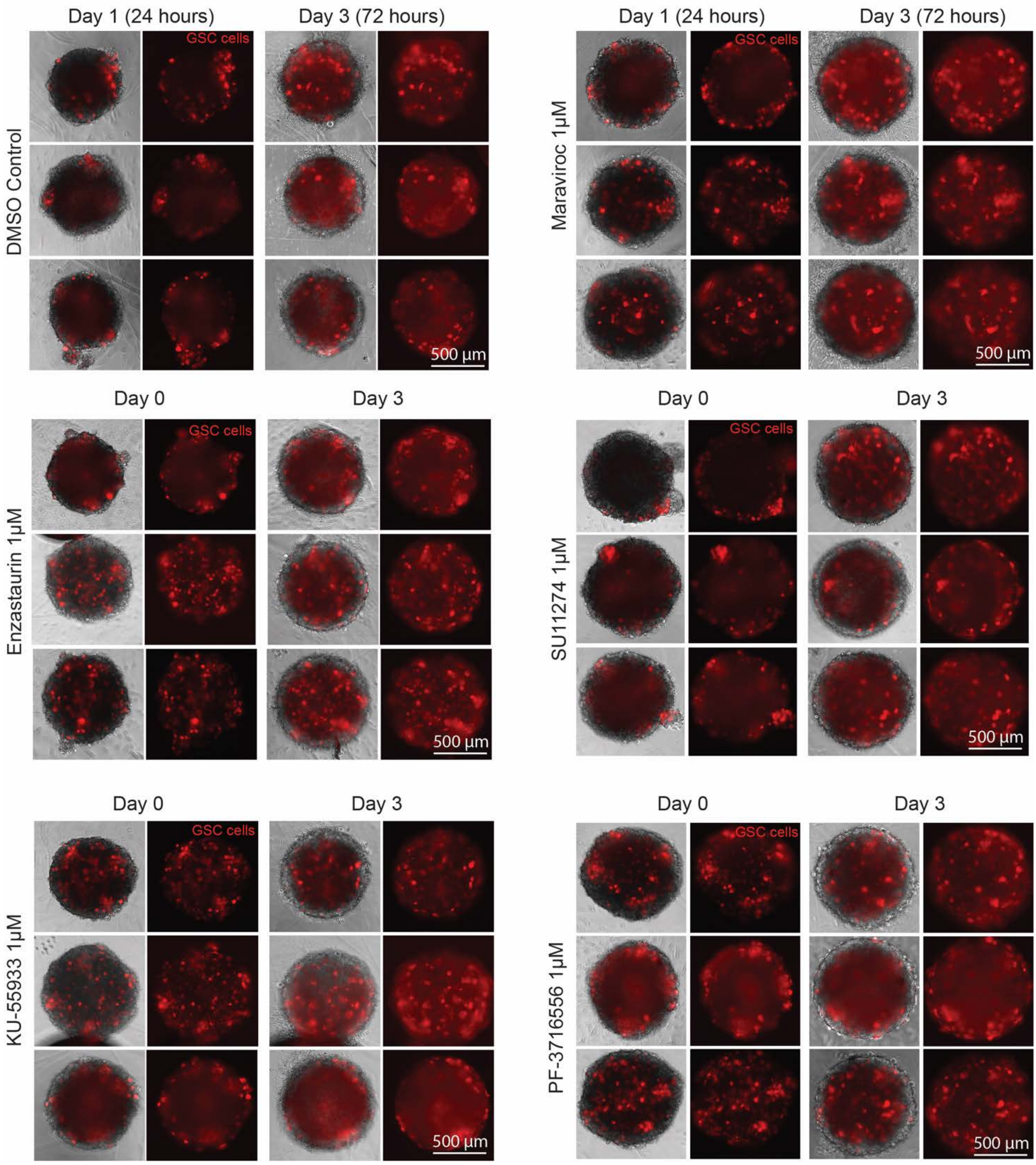

Figure S8 (Parts 1 and 2): Secondary screening of compounds that perturb GSC invasion in Hi-Q brain organoids.

Fig. S8 GBM screening, second part 2

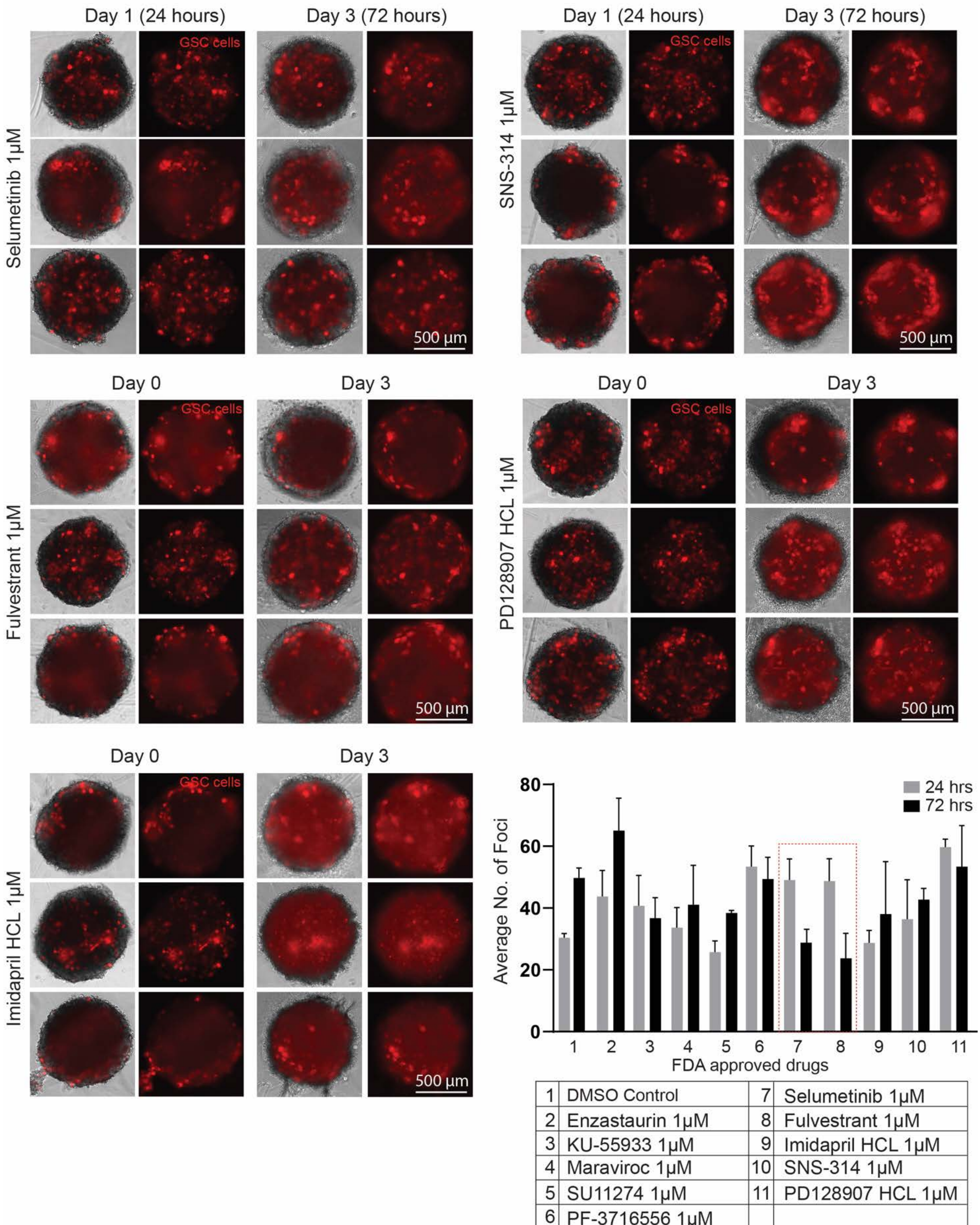

1	DMSO Control	7	Selumetinib 1μM
2	Enzastaurin 1μM	8	Fulvestrant 1μM
3	KU-55933 1μM	9	Imidapril HCL 1μM
4	Maraviroc 1μM	10	SNS-314 1μM
5	SU11274 1μM	11	PD128907 HCL 1μM
6	PF-3716556 1μM		

Testing of ten compounds (listed in the table at the end of the second part of the figure) for their ability to impair GSCs invasions in day 50 Hi-Q brain organoids. Patient-derived GSCs are labeled with mCherry. All experimental point includes three independent invasion assays with 1 μM of the selected compound. Invasions were measured at two different time points (24 and 72 hours). Note that the time point day 1 (or 24 hours) is 24 hours after seeding GSCs to brain organoids. The GSCs foci were calculated by automated imaging. The bar graph at the bottom quantifies the GSCs foci at 24 and 72 hours. The secondary screen identifies compounds 7 and 8 (Selumetinib and Fulvestrant) inhibiting GSC invasion (The dotted red box).

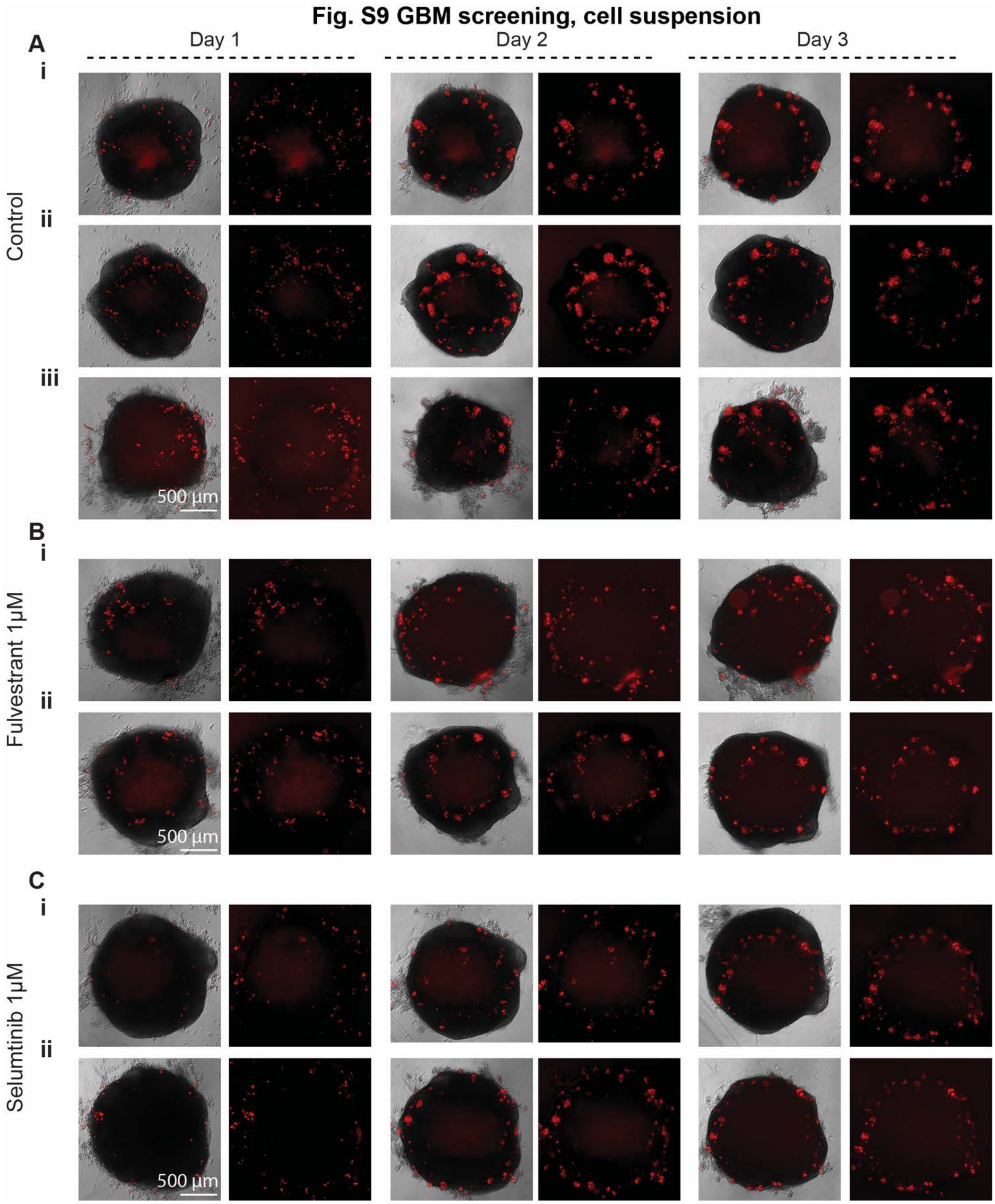

Figure S9: Testing Selumetinib and Fulvestrant for their ability to perturb GSCs invasions in Hi-Q brain organoids (as cell suspension at low resolution)

A. Kinetics of GSCs (red) invasions at day 0, day 1, and 2. Experiments were conducted in triplicate **(i-iii).** Panels show scale bars.

B-C. Exposure to 1µM Selumetinib **(i-ii)** and Fulvestrant **(i-ii)** appears to prevent GSC invasions into the organoids. Compared to untreated controls, drug-treated organoids show GSCs at the periphery of organoids. At least two representative organoid images are given. Panels show scale bar.

Fig. S10 GBM screening, spheres

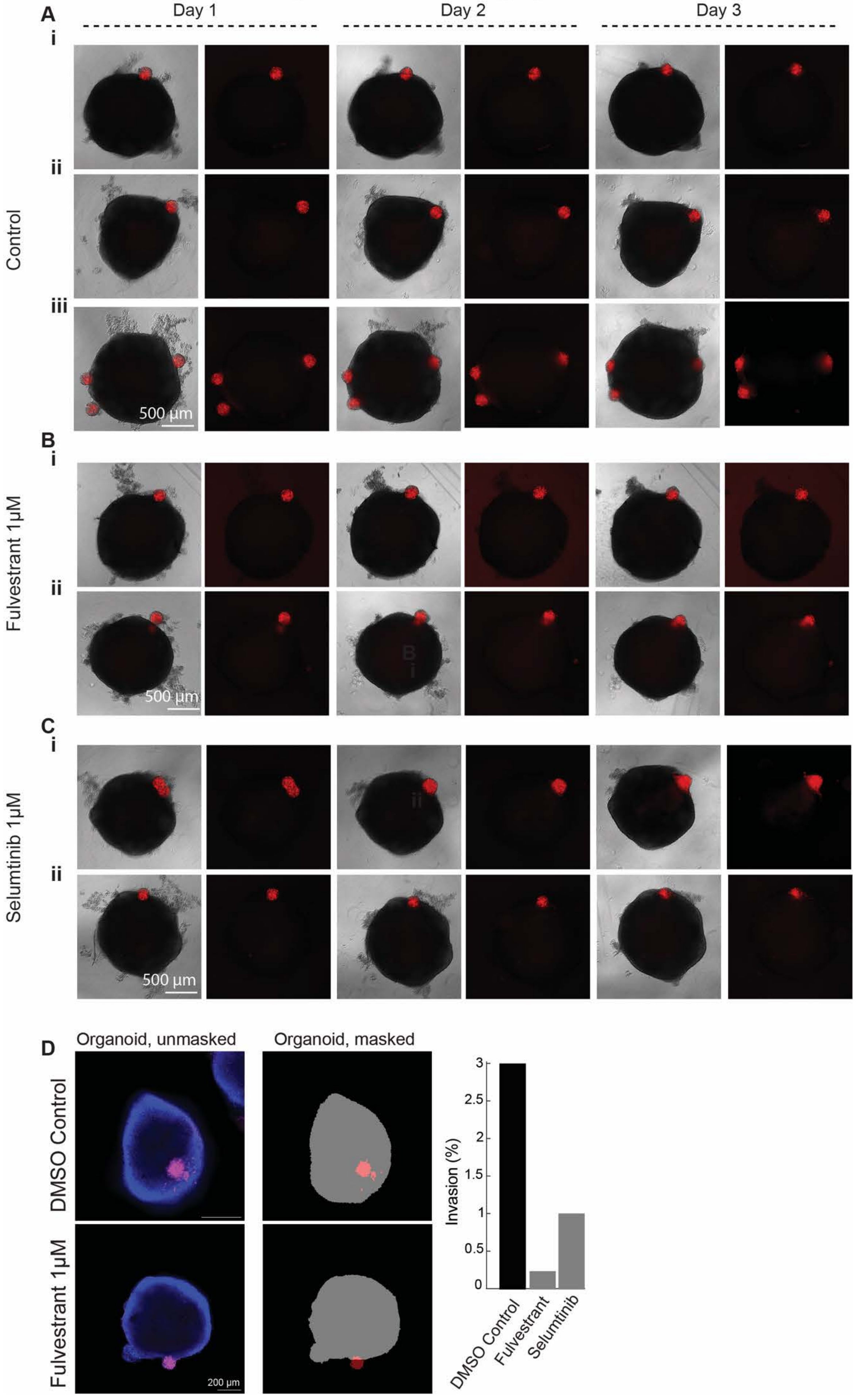

Figure S10: Testing Selumetinib and Fulvestrant for their ability to perturb GSCs invasions in Hi-Q brain organoids (as spheres at low resolution)
A. Kinetics of GSCs spheres (red) invasions on day 0, day 1, and 2. Experiments were conducted in triplicate **(i-iii).** GSC spheres integrate into organoids. Panels show scale bars.

B-C. Exposure to 1µM Selumetinib **(i-ii)** and Fulvestrant **(i-ii)** appears to prevent GSC spheres integration to organoids. Compared to untreated controls, drug-treated GSC spheres mainly stay at the edges of organoids. At least two representative organoid images are given. Panels show scale bar.

D. Computing the fraction of the organoid volume occupied by GSC spheres. A bar plot on the right shows the calculated percentage volume of organoids invaded by GSC spheres. Blue: Organoid. Magenta: GSC spheres.

Figure S11

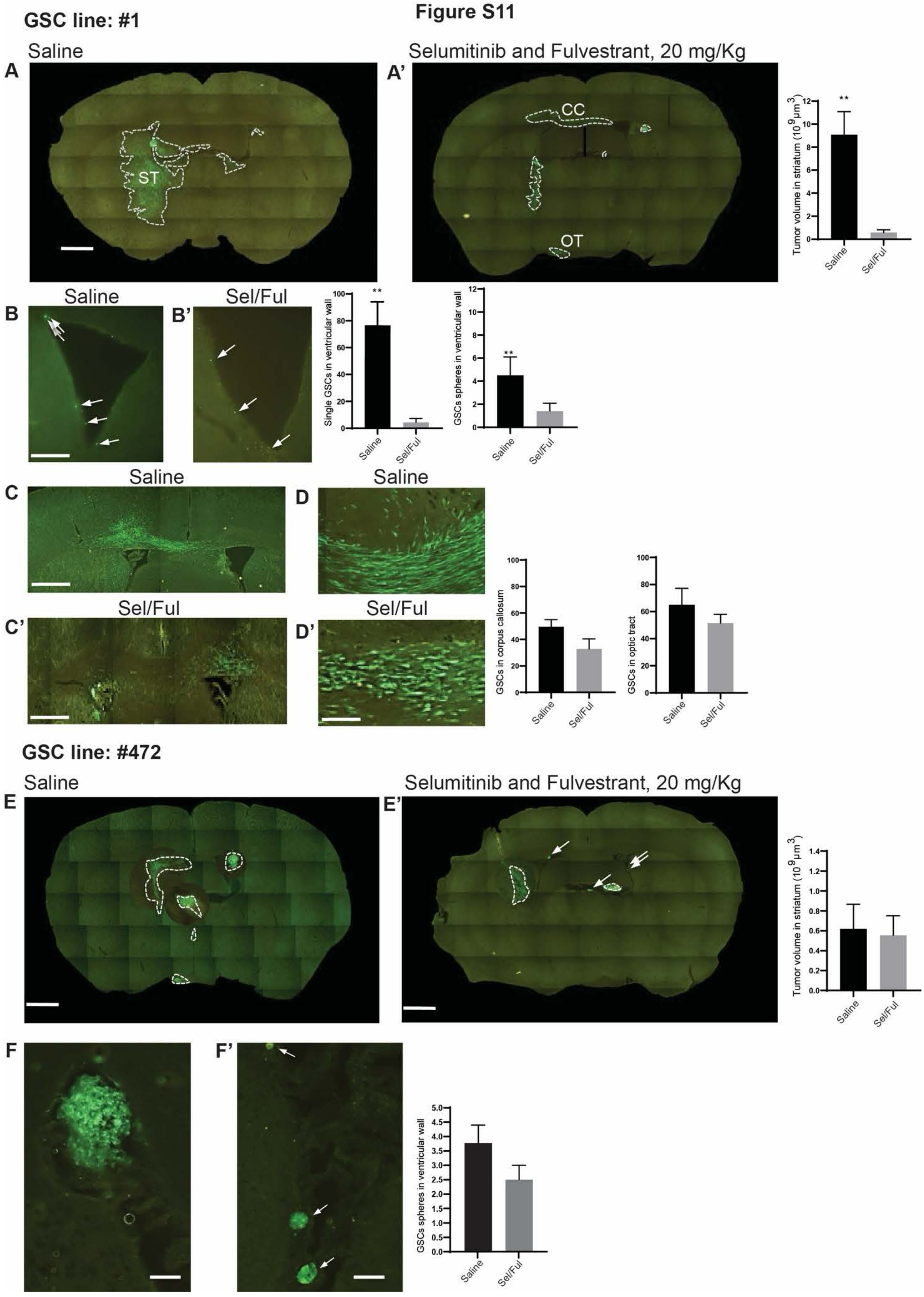

GSC line: #1
Saline
Selumitinib and Fulvestrant, 20 mg/Kg
A
A'
ST
CC
OT
Tumor volume in striatum ($10^9 \mu m^3$)
**
Saline
Sel/Ful
B
B'
Single GSCs in ventricular wall
GSCs spheres in ventricular wall
C
C'
D
D'
GSCs in corpus callosum
GSCs in optic tract
GSC line: #472
E
E'
F
F'

Figure S11: Combined treatment of Selumetinib and Fulvestrant perturb GSC (GSC line #1 and GSC line #472) invasion in mouse xenografts.
A-A'. In contrast to saline treatment, 20 mg/Kg treatment of Selumetinib and Fulvestrant **(A', right)** prevents integration of grafted GSC into the corpus callosum (CC) and optic tract (OT). Drug treatment reduces the tumor volume in the striatum. The bar diagram at right quantifies the tumor volume. Unpaired t-test and Mann-Whitney U test were used.

B-B'. Compared to saline treatment **(B)**, Selumetinib and Fulvestrant **(B', right)** prevent integration of grafted GSC into the ventricular wall. The bar diagram at right quantifies the integrated GSCs. Unpaired t-test and Mann-Whitney U test were used.

C-C'. In contrast to saline treatment **(C)**, Selumetinib and Fulvestrant **(C', bottom)** prevent grafted GSC integration into the corpus callosum ventricular wall. The bar diagram at right quantifies the integrated GSCs.

D-D'. Selumetinib and Fulvestrant treatment **(D', bottom)** prevents grafted GSC integration into the optic tract. Panel **D** (top) shows saline control. The bar diagram at right quantifies the number of GSCs integration between control and treated groups.

E-E'. Combined treatment of Selumetinib and Fulvestrant perturb GSC (GSC line #472) invasion in mouse xenografts. Selumetinib and Fulvestrant-treated animals **(E', right)** show a reduced tumor volume in the striatum. The saline-treated group is labeled as **E.** The bar diagram at right quantifies the tumor volume.

F-F'. Compared to saline treatment **(F)**, Selumetinib and Fulvestrant **(F', right)** prevent the integration of grafted GSC spheres in the ventricular wall. The bar diagram on the right shows the quantification.

Figure legend (Movie)

Movie 1: Bioreactor equipped with spinning arms to culture Hi-Q brain organoids. The movie shows suspending brain organoids.

Movie 2 (Related to main figure 3): Day 20 and day 60 Hi-Q brain organoids showing the distribution of various cell identity markers such as Acetylated a-tubulin, PSD97, SOX2, DCX, Synapsin 1, Tau, MAP2, Actin, P-Vimentin, Nestin and PCP4. Z-series stacks were collected after whole-mount staining, tissue clearing, and confocal imaging. Scale bar 500 µM.

Movie 3 (Related to main figure 5): Control (never frozen) and thawed Hi-Q brain organoid after cryopreservation showing the cytoarchitecture and distribution of dead cells labeled by TUNNEL. Z-series stacks were collected after whole-mount staining, tissue clearing, and confocal imaging. Scale bar 500 µM.

Supplementary Table 1. Media composition and incubation

Sr. No	Media Name	Media Composition/ company)	No. of Days in respective media
1	hiPSC maintenance medium	mTeSR-1 (Stem Cell Technologies)	Variable (up to 80% confluency)
2	Neural Induction medium	STEM Diff Neural induction medium (NIM) (Stem cell technologies, Catalog # 05835)	5
3	Wash media	DMEM/ F12	-
4	Neurosphere medium	1:1 DMEM/F12 and Neural Basal medium, supplemented with N2 (1:200), B27 w/o Vitamin A (1:100) (Thermo-scientific) 0.05mM MEM non-essential amino acids (Gibco), L-glutamine (1:100, Gibco), Pen-strep (100 µg/ml each), Insulin (0.2755 µM, Sigma Aldrich), 0.05 mM β-Mercaptoethanol (Life Technologies).	4
5	Human brain organoid differentiation medium	1:1 DMEM/F12 and Neural Basal medium, supplemented with N2 (1:200), B27 w/o Vitamin A (1:100) (Thermo-scientific) 0.05mM MEM non-essential amino acids (Gibco), L-glutamine (1:100, Gibco), Pen-strep (100 µg/ml each), Insulin (0.2755 µM, Sigma Aldrich), 0.05 mM β-Mercaptoethanol (Life Technologies), and supplemented with 5 µM SB431542 (Selleckchem, USA) and 0.5 µM Dorsomorphin (Sigma-Aldrich, USA)	21

6	Human brain organoid maturation medium	1:1 DMEM/F12 and Neural Basal medium, supplemented with N2 (1:200), B27 w/o Vitamin A (1:100) (Thermo-scientific) 0.05mM MEM non-essential amino acids (Gibco), L-glutamine (1:100, Gibco), Pen-strep (100 µg/ml each), Insulin (0.2755 µM, Sigma Aldrich), 0.05 mM β-Mercapto ethanol (Life Technologies)<br>Optional: Addition of neural differentiation or maturation factors such as retinoic acid, Brain derived neurotropic factor (BDNF), and Ciliary neurotrophic factor (CNTF).	~ 150-180

Supplementary Table 2. Organoid batches

Exp/ Batch no.	Researcher no.	hiPSC cell type	No. of neurospheres initiated (Approximate)	No. of organoids in spinner flasks (Approximate)	Status of experiment	Remarks	Status of Freeze/thaw
1	Researcher 1	IMR90 (WiCell Research Institute, RRID:CVCL_C436)	370	350	Successful	Neurospheres lost during neural induction and transfer to spinner flasks	Organoids were not frozen
2	Researcher 1	IMR90 (WiCell Research Institute, RRID:CVCL_C436)	1080	950	Successful	Fused organoids were discarded	Organoids were not frozen
3	Researcher 1	IMR90 (WiCell Research Institute, RRID:CVCL_C436)	370	298	Successful	-	20 organoids were freeze-thawed
4	Researcher 1	IMR90 (WiCell Research Institute, RRID:CVCL_C436)	740	725	Successful	Organoids disintegrated in spinner flasks	Organoids were not frozen
5	Researcher 1	IMR90 (WiCell Research Institute, RRID:CVCL_C436)	555	520	Successful	Neurospheres lost during neural induction	Organoids were not frozen
6	Researcher 1	IMR90 (WiCell Research Institute, RRID:CVCL_C436)	555	530	Successful	Fused organoids were discarded from spinner flasks	20 organoids were freeze-thawed

7	Researcher 1	IMR90 (WiCell Research Institute, RRID:CVCL_C436)	740	700	Successful	Fused organoids were discarded from spinner flasks	Organoids were not frozen
8	Researcher 1	Tubulin-GFP tagged hiPSCs (Coriell, Cat# AICS-0012)	370	345	Successful	Neurospheres lost during neural induction	Organoids were not frozen
9	Researcher 1	Tubulin-GFP tagged hiPSCs (Coriell, Cat# AICS-0012)	185	180	Successful	Fused organoids were discarded	Organoids were not frozen
10	Researcher 1	Tubulin-RFP tagged hiPSCs (Coriell Cat# AICS-0031-035)	370	360	Successful	Fused organoids were discarded	Organoids were not frozen
11	Researcher 1	Tubulin-RFP tagged hiPSCs (Coriell Cat# AICS-0031-035)	185	175	Successful	Neurospheres lost during transfer	Organoids were not frozen
12	Researcher 1	Crx-ips (PMID: 30100409)	370	355	Successful	Neurospheres lost during transfer	Organoids were not frozen
13	Researcher 1	Crx-ips (PMID: 30100409)	185	145	Successful	Organoids disintegrated (Reason unknown)	Organoids were not frozen
14	Researcher 1	Crx-ips (PMID: 30100409)	185	170	Successful	Neurospheres lost during transfer	Organoids were not frozen

15	Researcher 1	CDK5RAP2 ipsc	185	180	Successful	Neurospheres lost during transfer	Organoids were not frozen
16	Researcher 1	CDK5RAP2 ipsc	185	160	Successful	Neurospheres lost during transfer / Fused organoids were discarded	Organoids were not frozen
17	Researcher 1	CDK5RAP2 ipsc	185	180	Successful	-	Organoids were not frozen
18	Researcher 1	CSB-GM739 (PMID: 22904069)	370	350	Successful	Fused organoids were discarded	Organoids were not frozen
19	Researcher 1	CSB-GM739 (PMID: 22904069)	185	170	Successful	Neurospheres lost during transfer	Organoids were not frozen
20	Researcher 1	CSB-GM739 (PMID: 22904069)	185	175	Successful	Fused organoids were discarded	Organoids were not frozen
21	Researcher 1	CSB-GM739 (PMID: 22904069)	185	180	Successful	Fused organoids were discarded	Organoids were not frozen
22	Researcher 2	Tubulin-GFP tagged hiPSCs (Coriell, Cat# AICS-0012)	370	325	Successful	Neurospheres lost during neural induction	Organoids were not frozen
23	Researcher 2	Tubulin-GFP tagged hiPSCs (Coriell, Cat# AICS-0012)	370	350	Successful	Fused organoids were discarded	Organoids were not frozen
24	Researcher 2	Tubulin-RFP tagged hiPSCs	370	350	Successful	Neurospheres lost during neural induction	Organoids were not frozen

		(Coriell Cat# AICS-0031-035)					
26	Researcher 2	IMR90 (WiCell Research Institute, RRID:CVCL_C436)	370	100	Partially successful	Neurospheres lost during neural induction andmost organoids were collapsed	Organoids were not frozen
25	Researcher 2	IMR90 (WiCell Research Institute, RRID:CVCL_C436)	370	350	Successful	Fused organoids were discarded	Organoids were frozen
25	Researcher 2	Tubulin-GFP tagged hiPSCs (Coriell, Cat# AICS-0012)	720	700	Successful	Neurospheres lost during neural induction	Organoids were not frozen
26	Researcher 3	IMR90 (WiCell Research Institute, RRID:CVCL_C436)	550	520	Successful	Neurospheres lost during neural induction	Organoids were not frozen
27	Researcher 3	Tubulin-GFP tagged hiPSCs (Coriell, Cat# AICS-0012)	540	510	Successful	Fused organoids were discarded from spinner flasks	Organoids were freeze-thawed
28	Researcher 3	IMR90 (WiCell Research Institute, RRID:CVCL_C436)	720	700	Successful	Fused organoids were discarded from spinner flasks	Organoids were not frozen
29	Researcher 3	Tubulin-GFP tagged hiPSCs (Coriell, Cat# AICS-0012)	370	350	Successful	Neurospheres lost during neural induction	Organoids were not frozen

30	Researcher 3	Tubulin-GFP tagged hiPSCs (Coriell, Cat# AICS-0012)	370	180	Successful	Fused organoids were discarded	Organoids were not frozen
31	Researcher 3	Tubulin-RFP tagged hiPSCs (Coriell Cat# AICS-0031-035)	370	270	Successful	Fused organoids were discarded	Organoids were not frozen
32	Researcher 4	Tubulin-RFP tagged hiPSCs (Coriell Cat# AICS-0031-035)	185	175	Successful	Neurospheres lost during transfer	Organoids were not frozen
33	Researcher 4	IMR90 (WiCell Research Institute, RRID:CVCL_C436)	550	455	Successful	Neurospheres lost during transfer	Organoids were not frozen
34	Researcher 4	IMR90 (WiCell Research Institute, RRID:CVCL_C436)	365	320	Successful	Neurospheres lost during transfer	Organoids were not frozen
35	Researcher 4	IMR90 (WiCell Research Institute, RRID:CVCL_C436)	370	30	Failed	Organoids were collapsed due to unknown reasons	Organoids were not frozen
36	Researcher 5	IMR90 (WiCell Research Institute, RRID:CVCL_C436)	370	360	Successful	Neurospheres lost during transfer	Organoids were not frozen

37	Researcher 5	IMR90 (WiCell Research Institute, RRID:CVCL_C436)	370	350	Successful	Neurospheres lost during transfer	Organoids were not frozen
38	Researcher 6	Tubulin-GFP tagged hiPSCs (Coriell, Cat# AICS-0012)	925	880	Successful	Fused organoids were discarded	Organoids were not frozen
39	Researcher 6	IMR90 (WiCell Research Institute, RRID:CVCL_C436)	925	900	Successful	-	Organoids were frozen

Total number of neurospheres initiated: **17290**

Total number of organoids recovered to spinner flasks: **15373**

Recover/success rate: **88.9%**

Supplementary Table 3. Compound and plate details

Catalog Number	Product Name	barcode			M.w.	CAS Number	Target	DMSO (mg/mL) Max Solubility	DMSO (mM) Max Solubility	water (mg/mL) Max Solubility	water (mM) Max Solubility	URL	Information	Pathway	Formula	Form	Synonyms	SMILES	ALogP	HBA Count	HBD Count	RotatableBond
S1004	Veliparib (ABT-888)	44170800	b2	L3500-01	244,29	912444-00-9	PARP	17	70	<1		http://selleckchem.com/products/ABT-888.html	Veliparib (ABT-888) is a potent inhibitor of PARP1 and PARP2 with Ki of 5.2 nM and 2.9 nM in cell-free assays, respectively. It is inactive to SIRT2. Phase 3.	DNA Damage	C13H16N4O	free base	NSC 737664	C[C@@]1(CCCN1)C1=NC2=C(C=CC=C2N1)C(N)=O |r,c:11,13,t:7,9|	0,986	2	3	2
S1007	Roxadustat (FG-4592)	43888173	c2	L3500-01	352,34	808118-40-3	HIF	70	199	<1		http://selleckchem.com/products/fg-4592.html	Roxadustat (FG-4592) is an HIF-α prolyl hydroxylase inhibitor in a cell-free assay, stabilizes HIF-2 and induces EPO production. Phase 3.	Angiogenesis	C19H16N2O5	free base	ASP1517	CC1=NC(C(=O)NCC(O)=O)=C(O)C2=C1C=C(OC1=CC=CC=C1)C=C2 |c:13,21,23,26,t:1,10,16,19|	2,306	4	2	5
S1008	Selumetinib (AZD6244)	43974219	d2	L3500-01	457,68	606143-52-6	MEK	91	199	<1		http://selleckchem.com/products/AZD6244.html	Selumetinib (AZD6244) is a potent, highly selective MEK inhibitor with IC50 of 14 nM for MEK1 and Kd value of 530 nM for MEK2. It also inhibits ERK1/2 phosphorylation with IC50 of 10 nM, no inhibition to p38α, MKK6, EGFR, ErbB2, ERK2, B-Raf, etc. Phase 3.	MAPK	C17H15BrClFN4O3	free base	ARRY-142886	CN1C=NC2=C(F)C(NC3=CC=C(Br)C=C3Cl)=C(C=C12)C(=O)NOCCO |c:2,4,14,17,t:9,11,19|	3,234	3	3	6
S1014	Bosutinib (SKI-606)	44198714	e2	L3500-01	530,45	380843-75-4	Src	100	189	<1		http://selleckchem.com/products/Bosutinib.html	Bosutinib (SKI-606) is a novel, dual Src/Abl inhibitor with IC50 of 1.2 nM and 1 nM in cell-free assays, respectively.	Angiogenesis	C26H29Cl2N5O3	free base	N/A	COC1=C(Cl)C=C(Cl)C(NC2=C(C=NC3=CC(OCCCN4CCN(C)CC4)=C(OC)C=C23)C#N)=C1 |c:2,12,37,t:5,10,14,28,32|	4,883	4	1	9
S1020	PD184352 (CI-1040)	44134638	f2	L3500-01	478,67	212631-79-3	MEK	96	201	<1		http://selleckchem.com/products/CI-1040-(PD184352).html	PD184352 (CI-1040) is an ATP non-competitive MEK1/2 inhibitor with IC50 of 17 nM in cell- based assays, 100-fold more selective for MEK1/2 than MEK5. Phase 2.	MAPK	C17H14ClF2IN2O2	free base	N/A	FC1=CC=C(C(=O)NOCC2CC2)C(NC2=CC=C(I)C=C2Cl)=C1F |c:21,24,t:1,3,16,18|	4,907	2	2	6
S1022	Ridaforolimus (Deforolimus, MK-8669)	44110983	g2	L3500-01	990,21	572924-54-0	mTOR	198	200	<1		http://selleckchem.com/products/Deforolimus.html	Ridaforolimus (Deforolimus, MK-8669) is a selective mTOR inhibitor with IC50 of 0.2 nM in HT-1080 cell line while not classified as a prodrug, mTOR inhibition and FKBP12 binding is similar to rapamycin. Phase 3.	PI3K/Akt/mTOR	C53H84NO14P	free base	AP23573	[H][C@@]12CC[C@@H](C)[C@@](O)(O1)C(=O)C(=O)N1CCCC[C@@]1([H])C(=O)O[C@@H](CC(=O)[C@H](C)\C=C(C)\[C@@H](O)[C@@H](OC)C(=O)[C@H](C)C[C@H](C)\C=C\C=C\C=C(C)\[C@H](C2)OC)[C@H](C)C[C@@H]1CC[C@@H](OP(C)(C)=O)[C@@H](C1)OC |r,c:31,50,t:46,48|	6,303	12	1	8
S1029	Lenalidomide (CC-5013)	44095531	b3	L3500-01	259,26	191732-72-6	TNF-alpha	52	201	<1		http://selleckchem.com/products/Lenalidomide.ht	Lenalidomide (CC-5013) is a TNF-α secretion inhibitor with IC50 of 13 nM in PBMCs.	Apoptosis	C13H13N3O3	free base	N/A	NC1=C2CN(C3CCC(=O)NC3=O)C(=O)C2=CC=C1 |c:1,17,19|	-0,515	3	2	1

												ml										
S1033	Nilotinib (AMN-107)	43879800	c3	L3500-01	529,52	641571-10-0	Bcr-Abl	27	51	<1		http://selleckchem.com/products/Nilotinib.html	Nilotinib (AMN-107) is a selective Bcr-Abl inhibitor with IC50 less than 30 nM in Murine myeloid progenitor cells.	Angiogenesis	C28H22F3N7O	free base	N/A	CC1=CN(C=N1)C1=CC(NC(=O)C2=CC=C(C)C(NC3=NC=CC(=N3)C3=CN=CC=C3)=C2)=CC(=C1)C(F)(F) F |c:4,22,24,29,31,33,35,37,t:1,7,13,1 5,20,27|	5,084	5	2	7
S1036	PD0325901	44194817	d3	L3500-01	482,19	391210-10-9	MEK	96	199	<1		http://selleckchem.com/products/PD-0325901.html	PD0325901 is a selective and non ATP-competitive MEK inhibitor with IC50 of 0.33 nM in cell-free assays, roughly 500-fold more potent than CI- 1040 on phosphorylation of ERK1 and ERK2. Phase 2.	MAPK	C16H14F3IN2O4	free base	N/A	OC[C@@H](O)CONC(=O)C1=C(NC2=CC=C(I)C=C2F)C(F)=C(F)C=C 1 |r,c:9,17,25,t:12,14,22|	2,578	2	4	7
S1041	STF-62247	43518340	e3	L3500-01	267,35	315702-99-9	Autophagy	53	198	<1		http://selleckchem.com/products/STF-62247.html	STF-62247 is a molecule targeting VHL-deficient renal cell carcinoma that induces autophagy. STF-62247 shows selective toxicity and growth inhibition of renal cells lacking VHL 25-fold greater sensitivity observed for cells with VHL deficiency compared to wild-type (VHL+).	Autophagy	C15H13N3S	free base	N/A	CC1=CC(NC2=NC(=CS2)C2=CC=NC=C2)=CC=C1 |c:7,13,15,17,19,t:1,5,1 1|	3,659	2	1	3
S1044	Temsirolimus (CCI-779, NSC 683864)	44075746	f3	L3500-01	1030,29	162635-04-3	mTOR	67	65	<1		http://selleckchem.com/products/Temsirolimus.ht ml	Temsirolimus (CCI-779, NSC 683864) is a specific mTOR inhibitor with IC50 of 1.76 µM in a cell-free assay.	PI3K/Akt/mTOR	C56H87NO16	free base	N/A	CO[C@@H]1C[C@H](C[C@@H](C)[C@@H]2CC(=O)[C@H](C)\C=C(C)\[C@@H](O)[C@@H](OC)C(=O)[C@H](C)C[C@H](C)\C=C\C=C\C=C(C)\[C@H](C[C@@H]3CC[C@@H](C)[C@@](O)(O3)C(=O)C(=O)N3CCCC[C@H]3C(=O)O2)OC)CC[C@H]1OC(=O)C(C)(CO)CO |r,c:14,33,t:29,31|	6,039	12	3	11
S1048	Tozasertib (VX-680, MK-0457)	43883500	g3	L3500-01	464,59	639089-54-6	Aurora Kinase	93	200	<1		http://selleckchem.com/products/VX-680(MK-0457).html	Tozasertib (VX-680, MK-0457) is a pan-Aurora inhibitor, mostly against Aurora A with Kiapp of 0.6 nM in a cell-free assay, less potent towards Aurora B/Aurora C and 100-fold more selective for Aurora A than 55 other kinases. Phase 2.	Cell Cycle	C23H28N8OS	free base	N/A	CN1CCN(CC1)C1=NC(SC2=CC=C(NC(=O)C3CC3)C=C2)=NC(NC2=NNC(C)=C2)=C1 |c:23,25,33,35,t:8,12,14,29|	4,445	4	3	7
S1055	Enzastaurin (LY317615)	44089519	b4	L3500-01	515,61	170364-57-5	PKC	30	58	<1		http://selleckchem.com/products/Enzastaurin.html	Enzastaurin (LY317615) is a potent PKCβ selective inhibitor with IC50 of 6 nM in cell-free assays, 6- to 20-fold selectivity against PKCα, PKCγ and PKCε. Phase 3.	TGF-beta/Smad	C32H29N5O2	free base	N/A	CN1C=C(C2=CC=CC=C12)C1=C(C(=O)NC1=O)C1=CN(C2CCN(CC3=NC=CC=C3)CC2)C2=CC=CC=C12 |c:2,6,30,32,40,t:4,8,12,20,28,38,42|	4,32	3	1	5
S1065	Pictilisib (GDC-0941)	44117299	c4	L3500-01	513,64	957054-30-7	PI3K	44	86	<1			Pictilisib (GDC-0941) is a potent inhibitor of PI3Kα/δ with IC50 of 3 nM in cell-free assays, with modest	PI3K/Akt/mT	C23H27N7O	free base	RG7321		2,153	6	1	5

												http://selleckchem.com/products/GDC-0941.html	selectivity against p110β (11-fold) and p110γ (25-fold). Phase 2.	OR	3S2			CS(=O)(=O)N1CCN(CC2=CC3=NC(=NC(N4CCOCC4)=C3S2)C2=CC=CC3=C2C=NN3)CC1 |c:[illegible],22,29,31,34,t:9,11,27|				
S1067	SB431542	43525502	d4	L3500-01	384,39	301836-41-9	TGF-beta/Smad	76	198	<1		http://selleckchem.com/products/SB-431542.html	SB431542 is a potent and selective inhibitor of ALK5 with IC50 of 94 nM in a cell-free assay, 100- fold more selective for ALK5 than p38 MAPK and other kinases.	TGF-beta/Smad	C22H16N4O3	free base	N/A	NC(=O)C1=CC=C(C=C1)C1=NC(=C(N1)C1=NC=CC=C1)C1=CC2=C(OCO2)C=C1 |c:5,7,12,18,20,31,t:3,10,16,23,25|	3,447	5	2	4
S1069	Luminespib (AUY-922, NVP-AUY922)	44064612	e4	L3500-01	465,54	747412-49-3	HSP (e.g. HSP90)	93	200	<1		http://selleckchem.com/products/NVP-AUY922.html	Luminespib (AUY-922, NVP-AUY922) is a highly potent HSP90 inhibitor for HSP90α/β with IC50 of 13 nM /21 nM in cell- free assays, weaker potency against the HSP90 family members GRP94 and TRAP-1, exhibits the tightest binding of any small-molecule HSP90 ligand. Phase 2.	Cytoskeletal Signaling	C26H31N3O5	free base	VER-52296	CCNC(=O)C1=NOC(=C1C1=CC=C(CN2CCOCC2)C=C1)C1=CC(C(C)C)=C(O)C=C1O |c:8,23,34,t:5,11,13,26,31|	4,069	4	3	7
S1070	PHA-665752	43915671	f4	L3500-01	641,61	477575-56-7	c-Met	128	199	<1		http://selleckchem.com/products/PHA-665752.html	PHA-665752 is a potent, selective and ATP-competitive c-Met inhibitor with IC50 of 9 nM in cell-free assays, >50-fold selectivity for c-Met than RTKs or STKs.	Protein Tyrosine Kinase	C32H34Cl2N4O4S	free base	N/A	CC1=C(C(=O)N2CCC[C@@H]2CN2CCCC2)C(C)=C(N1)\C=C1/C(=O)NC2=C1C=C(C=C2)S(=O)(=O)CC1=C(Cl)C=CC=C1Cl |r,c:1,19,28,31,33,40,43,45|	5,689	4	2	7
S1071	HA14-1	43446833	g4	L3500-01	409,23	65673-63-4	Bcl-2	82	200	<1		http://selleckchem.com/products/HA14-1.html	HA14-1 is a non-peptidic ligand of a Bcl-2 surface pocket with IC50 of ~9 μM.	Apoptosis	C17H17BrN2O5	free base	N/A	[H][C@@]1([C@H](C#N)C(=O)OCC)C2=CC(Br)=CC=C2OC(N)=C1C(= O)OCC |r,c:13,15,20,t:10|	2,511	5	1	7
S1078	MK-2206 2HCl	44116475	b5	L3500-01	480,39	1032350-13-2	Akt	14	29	<1		http://selleckchem.com/products/MK-2206.html	MK-2206 2HCl is a highly selective inhibitor of Akt1/2/3 with IC50 of 8 nM/12 nM/65 nM in cell- free assays, respectively no inhibitory activities against 250 other protein kinases observed. Phase 2.	PI3K/Akt/mTOR	C25H23Cl2N5O	Dihydrochloride	N/A	Cl.Cl.NC1(CCC1)C1=CC=C(C=C1)C1=C(C=C2C3=NNC(=O)N3C=CC2=N1)C1=CC=CC=C1 |c:8,10,24,27,32,34,t:6,13,15,17,30|	4,183	3	2	3
S1080	SU11274	43903025	c5	L3500-01	568,09	658084-23-2	c-Met	92	162	<1		http://selleckchem.com/products/SU11274.html	SU11274 is a selective Met inhibitor with IC50 of 10 nM in cell-free assays, no effects on PGDFRβ, EGFR or Tie2.	Protein Tyrosine Kinase	C28H30ClN5O4S	free base	PKI-SU11274	CN(C1=CC(Cl)=CC=C1)S(=O)(=O)C1=CC2=C(NC(=O)\C2=C/C2=C(C)C(C(=O)N3CCN(C)CC3)=C(C)N2)C=C1 |c:5,7,23,41,t:2,13,15,36|	3,477	4	2	5
S1085	Belinostat (PXD101)	43953686	d5	L3500-01	318,35	414864-00-9	HDAC	64	201	<1		http://selleckchem.com/products/Belinostat.html	Belinostat (PXD101) is a novel HDAC inhibitor with IC50 of 27 nM in a cell-free assay, with activity demonstrated in cisplatin-resistant tumors.	Epigenetics	C15H14N2O4S	free base	NSC726630, PX-105684	ONC(=O)\C=C\C1=CC(=CC=C1)S(=O)(=O)NC1=CC=CC=C1 |c:8,10,19,21,t:6,17|	1,8	3	2	5
S1087	Iniparib (BSI-201)	44076148	e5	L3500-01	292,03	160003-66-7	PARP	58	199	<1		http://selleck	Iniparib (BSI-201) is a PARP1 inhibitor with demonstrated effectiveness in triple- negative breast cancer (TNBC). Phase 3.	DNA Damage	C7H5IN2O3	free base	NSC-746045, IND-71677	NC(=O)C1=CC(=C(I)C=C	1,304	1	1	2

												chem.com/products/BSI-201.html						1)[N+]([O-])=O |c:8,t:3,5|				
S1089	Refametinib (RDEA119, Bay 86-9766)	44117407	f5	L3500-01	572,34	923032-37-5	MEK	100	175	<1		http://selleckchem.com/products/refametinib-rdea119-bay86-9766.html	Refametinib (RDEA119, Bay 86-9766) is a potent, ATP non-competitive and highly selective inhibitor of MEK1 and MEK2 with IC50 of 19 nM and 47 nM, respectively.	MAPK	C19H20F3IN2O5S	free base	BAY 869766	COC1=C(NS(=O)(=O)C2(C[C@H](O)CO)CC2)C(NC2=CC=C(I)C=C2F)=C(F)C(F)=C1 |r,c:2,24,31,t:19,21,27|	2,827	3	4	9
S1091	Linsitinib (OSI-906)	44164514	g5	L3500-01	421,49	867160-71-2	IGF-1R	84	199	<1		http://selleckchem.com/products/OSI-906.html	Linsitinib (OSI-906) is a selective inhibitor of IGF-1R with IC50 of 35 nM in cell-free assays modestly potent to InsR with IC50 of 75 nM, and no activity towards Abl, ALK, BTK, EGFR, FGFR1/2, PKA etc. Phase 3.	Protein Tyrosine Kinase	C26H23N5O	free base	N/A	C[C@@]1(O)C[C@@H](C1)C1=NC(=C2N1C=CN=C2N)C1=CC=C2C=CC(=NC2=C1)C1=CC=CC=C1 |r,c:9,13,15,23,25,28,33,35,t:7,19,21,31|	3,757	3	1	3
S1092	KU-55933 (ATM Kinase Inhibitor)	44083279	b6	L3500-01	395,49	587871-26-9	ATM/ATR	33	83	<1		http://selleckchem.com/products/KU-55933.html	KU-55933 (ATM Kinase Inhibitor) is a potent and specific ATM inhibitor with IC50/Ki of 12.9 nM/2.2 nM in cell-free assays, and is highly selective for ATM as compared to DNA-PK, PI3K/PI4K, ATR and mTOR.	DNA Damage	C21H17NO3S2	free base	N/A	O=C1C=C(OC(=C1)C1=CC=CC2=C1SC1=CC=CC=C1S2)N1CCOCC1 |c:2,5,10,12,18,20,t:8,16|	4,393	3	0	2
S1093	GSK1904529A	42372696	c6	L3500-01	851,96	1089283-49-7	IGF-1R	124	146	<1		http://selleckchem.com/products/GSK1904529A.html	GSK1904529A is a selective inhibitor of IGF-1R and IR with IC50 of 27 nM and 25 nM in cell-free assays, >100-fold more selective for IGF-1R/InsR than Akt1/2, Aurora A/B,B-Raf, CDK2, EGFR etc.	Protein Tyrosine Kinase	C44H47F2N9O5S	free base	GSK 4529	CCC1=CC(NC2=NC(=CC=N2)C2=C(N=C3C=CC=CN23)C2=CC(C(=O)NC3=C(F)C=CC=C3F)=C(OC)C=C2)=C(OC)C=C1N1CCC(CC1)N1CCN(CC1)S(C)(=O)=O |c:8,10,17,19,30,33,35,42,48,t:2,6,13,15,24,38,44|	6,571	8	2	12
S1094	PF-04217903	43522775	d6	L3500-01	372,38	956905-27-4	c-Met	5	13	<1		http://selleckchem.com/products/PF-04217903.html	PF-04217903 is a selective ATP-competitive c-Met inhibitor with IC50 of 4.8 nM in A549 cell line, susceptible to oncogenic mutations (no activity to Y1230C mutant). Phase 1.	Protein Tyrosine Kinase	C19H16N8O	free base	N/A	OCCN1C=C(C=N1)C1=NC2=C(N=NN2CC2=CC3=CC=CN=C3C=C2)N=C1 |c:4,6,13,22,24,27,30,t:9,11,18,20|	1,561	6	1	5
S1097	BTZ043 Racemate	43732124	e6	L3500-01	431,39	957217-65-1	Anti-infection	22	51	<1		http://selleckchem.com/products/BTZ043.html	BTZ043 racemate is a decaprenylphosphoryl-β-D-ribose 2'-epimerase (DprE1) inhibitor acting as a new antimycobacterial agent that kill Mycobacterium tuberculosis.	Microbiology	C17H16F3N3O5S	free base	N/A	CC1COC2(CCN(CC2)C2=NC(=O)C3=CC(=CC([N+]([O-])=O)=C3S2)C(F)(F)F)O1 |c:17,22,t:11,15|	3,515	4	0	3

S1100	MLN8054	44075910	f6	L3500-01	476,86	869363-13-3	Aurora Kinase	95	199	<1		http://selleckchem.com/products/MLN8054.html	MLN8054 is a potent and selective inhibitor of Aurora A with IC50 of 4 nM in Sf9 insect cell. It is more than 40-fold selective for Aurora A than Aurora B. Phase 1.	Cell Cycle	C25H15ClF2N4O2	free base	N/A	OC(=O)C1=CC=C(NC2=NC3=C(CN=C(C4=CC(Cl)=CC=C34)C3=C(F)C=CC=C3F)C=N2)C=C1 |c:13,18,24,27,29,33,36,t:3,5,8,10,1 5,20|	6,192	4	1	4
S1103	ZM 447439	44138247	g6	L3500-01	513,59	331771-20-1	Aurora Kinase	103	201	<1		http://selleckchem.com/products/ZM-447439.html	ZM 447439 is a selective and ATP-competitive inhibitor for Aurora A and Aurora B with IC50 of 110 nM and 130 nM, respectively. It is more than 8-fold selective for Aurora A/B than MEK1, Src, Lck and has little effect against CDK1/2/4, Plk1, Chk1, etc.	Cell Cycle	C29H31N5O4	free base	N/A	COC1=C(OCCCN2CCOCC2)C=C2N=CN=C(NC3=CC=C(NC(=O)C4=CC=CC=C4)C=C3)C2=C1 |c:2,17,31,33,36,40,t:15,19,22,24,29 |	4,118	6	2	10
S1106	OSU-03012 (AR-12)	42582476	b7	L3500-01	460,45	742112-33-0	PDK	11	24	<1		http://selleckchem.com/products/OSU-03012.html	OSU-03012 (AR-12) is a potent inhibitor of recombinant PDK- 1(phosphoinositide-dependent kinase 1) with IC50 of 5 μM in a cell- free assay and 2-fold increase in potency over OSU-02067.	PI3K/Akt/mTOR	C26H19F3N4O	free base	N/A	NCC(=O)NC1=CC=C(C=C1)N1N=C(C=C1C1=CC2=C(C=C1)C1=C(C=CC=C1)C=C2)C(F)(F)F |c:7,9,13,15,20,22,27,29,32,t:5,18,25|	5,212	2	2	5
S1109	BI 2536	43442221	c7	L3500-01	521,66	755038-02-9	PLK	21	40	<1		http://selleckchem.com/products/BI-2536.html	BI2536 is a potent Plk1 inhibitor with IC50 of 0.83 nM in a cell-free assay. It shows 4- and 11-fold greater selectivity against Plk2 and Plk3. Phase 2.	Cell Cycle	C28H39N7O3	free base	N/A	CC[C@H]1N(C2CCCC2)C2=NC(NC3=CC=C(C=C3OC)C(=O)NC3CCN(C)CC3)=NC=C2N(C)C1=O |r,c:16,18,33,35,t:10,14|	3,548	5	2	7
S1110	Varespladib (LY315920)	44134417	d7	L3500-01	380,39	172732-68-2	Phospholipase (e.g. PLA)	76	200	<1		http://selleckchem.com/products/LY315920(Varespladib).html	Varespladib (LY315920) is a potent and selective human non-pancreatic secretory phospholipase A2 (hnsPLA) inhibitor with IC50 of 7 nM. Phase 3.	Metabolism	C21H20N2O5	free base	N/A	CCC1=C(C(=O)C(N)=O)C2=C(C=CC=C2OCC(O)=O)N1CC1=CC=CC=C1 |c:2,11,13,26,28,t:9,24|	3,078	4	1	8
S1113	GSK690693	43976832	e7	L3500-01	425,48	937174-76-0	Akt	39	92	<1		http://selleckchem.com/products/GSK690693.html	GSK690693 is a pan-Akt inhibitor targeting Akt1/2/3 with IC50 of 2 nM/13 nM/9 nM in cell- free assays, also sensitive to the AGC kinase family: PKA, PrkX and PKC isozymes. Phase 1.	PI3K/Akt/mTOR	C21H27N7O3	free base	N/A	CCN1C(=NC2=C1C(OC[C@H]1CCCNC1)=CN=C2C#CC(C)(C)O)C1=NON=C1N |r,c:3,5,17,19,31,t:28|	1,839	6	2	7
S1115	Odanacatib (MK-0822)	42513989	f7	L3500-01	525,56	603139-19-1	Cysteine Protease	100	190	<1		http://selleckchem.com/p	Odanacatib (MK 0822) is a potent, selective, and neutral inhibitor of cathepsin K (human/rabbit) with IC50 of 0.2 nM/1 nM, and demonstrated high selectivity versus off-target cathepsin B, L, S. Phase 3.	Proteases	C25H27F4N3O3S	free base	N/A	CC(C)(F)C[C@H](N[C@@H](C1=C	3,978	3	2	10

												roducts/Odanacatib-(MK0822).html						C=C(C=C1)C1=CC=C(C=C1)S(C)(=O)=O)C(F)(F)F)C(=O)NC1(CC1)C#N |r,c:10,12,17,19,t:8,15|				
S1120	Everolimus (RAD001)	44051501	g7	L3500-01	958,22	159351-69-6	mTOR	100	104	<1		http://selleckchem.com/products/Everolimus(RAD001).html	Everolimus (RAD001) is an mTOR inhibitor of FKBP12 with IC50 of 1.6-2.4 nM in a cell-free assay.	PI3K/Akt/mTOR	C53H83NO14	free base	N/A	CO[C@@H]1C[C@H](C[C@H](C)[C@@H]2CC(=O)[C@H](C)\C=C(C)\[C@@H](O)[C@@H](OC)C(=O)[C@H](C)C[C@H](C)\C=C\C=C\C=C(C)\[C@H](C[C@@H]3CC[C@@H](C)[C@@](O)(O3)C(=O)C(=O)N3CCCC[C@H]3C(=O)O2)OC)CC[C@H] 1OCCO |r,c:14,33,t:29,31|	6,164	11	2	9
S1129	SRT1720 HCl	43662390	b8	L3500-01	506,02	1001645-58-4	Sirtuin	38	75	<1		http://selleckchem.com/products/SRT1720.html	SRT1720 HCl is a selective SIRT1 activator with EC50 of 0.16 μM in a cell-free assay, but is >230-fold less potent for SIRT2 and SIRT3.	Epigenetics	C25H24ClN7OS	Hydrochloride	N/A	Cl.O=C(NC1=CC=CC=C1C1=CN2C(CN3CCNCC3)=CSC2=N1)C1=NC2=CC=CC=C2N=C1 |c:5,7,21,25,32,34,37,t:3,10,28,30|	3,018	4	2	5
S1130	YM155 (Sepantronium Bromide)	44050248	c8	L3500-01	443,29	781661-94-7	Survivin	55	124	89	201	http://selleckchem.com/products/YM155.html	YM155 (Sepantronium Bromide) is a potent survivin suppressant by inhibiting Survivin promoter activity with IC50 of 0.54 nM in HeLa-SURP-luc and CHO- SV40-luc cells does not significantly inhibit SV40 promoter activity, but is observed to slightly inhibit the interaction of Survivin with XIAP. Phase 2.	Apoptosis	C20H19BrN4O3	Bromide	N/A	[Br-].COCCN1C(C)=[N+](CC2=CN=CC=N2)C2=C1C(=O)C1=C(C=CC=C1) C2=O |c:11,13,16,23,25,t:6,9,21|	-0,733	5	0	5
S1139	ADL5859 HCl	42681876	d8	L3500-01	428,95	850173-95-4	Opioid Receptor	86	200	5	12	http://selleckchem.com/products/ADL-5859.html	ADL5859 HCl is a δ- opioid receptor agonist with Ki of 0.8 nM, selectivity against opioid receptor κ, μ, and weak inhibitory activity at the hERG channel. Phase 2.	Neuronal Signaling	C24H29ClN2O3	Hydrochloride	N/A	Cl.CCN(CC)C(=O)C1=CC=C(C=C1)C1=CC2(CCNCC2)OC2=CC=CC(O)=C2 |c:9,11,26,t:7,14,24,29|	3,196	2	2	4
S1141	Tanespimycin (17-AAG)	44057877	e8	L3500-01	585,69	75747-14-7	HSP (e.g. HSP90)	100	171	<1		http://selleckchem.com/products/17-AAG(Geldanamycin).html	Tanespimycin (17-AAG) is a potent HSP90 inhibitor with IC50 of 5 nM in a cell-free assay, having a 100-fold higher binding affinity for HSP90 derived from tumour cells than HSP90 from normal cells. Phase 2.	Cytoskeletal Signaling	C31H43N3O8	free base	27374,NSC-330507, KOS	CO[C@H]1C[C@H](C)CC2=C(NCC=C)C(=O)C=C(NC(=O)\C(C)=C\C=C/[C@H](OC)[C@@H](OC(N)=O)\C(C)=C\[C@H](C)[C@H]1O)C2=O |r,c:7,23,t:15,21,34|	2,524	7	4	7
S1144	Ivacaftor (VX-770)	43941753	f8	L3500-01	392,49	873054-44-5	CFTR	78	199	<1		http://selleckchem.com/p	Ivacaftor (VX-770) is a selective potentiator of CFTR targeting G551D- CFTR and F508del- CFTR with EC50 of 100 nM and 25 nM in fisher rat thyroid cells, respectively.	Transmembrane Transp	C24H28N2O3	free base	N/A	CC(C)(C)C1=CC(=C(NC(=O)C2=CNC3=C(C=CC=C3)C2=O	4,516	2	3	4

												roducts/VX-770.html		orte rs				)C=C1O)C (C)(C)C |c:16,18,24,t:4,6,11,14|				
S1145	SNS-032 (BMS-387032)	43881428	g8	L3500-01	380,53	345627-80-7	CDK	76	200	<1		http://selleckchem.com/products/SNS-032.html	SNS-032 (BMS-387032) has firstly been described as a selective inhibitor of CDK2 with IC50 of 48 nM in cell-free assays and is 10- and 20- fold selective over CDK1/CDK4. It is also found to be sensitive to CDK7/9 with IC50 of 62 nM/4 nM, with little effect on CDK6. Phase 1.	Cell Cycle	C17H24N4O2S2	free base	N/A	CC(C)(C)C1=CN=C(CSC2=CN=C(NC(=O)C3CCNCC3)S2)O1 |t:4,6,10,12|	2,07	4	2	6
S1154	SNS-314	42602019	b9	L3500-01	527,04	1146618-41-8	Aurora Kinase	105	199	6	11	http://selleckchem.com/products/sns-314-mesylate.html	SNS-314 is a potent and selective inhibitor of Aurora A, Aurora B and Aurora C with IC50 of 9 nM, 31 nM, and 3 nM, respectively. It is less potent to Trk A/B, Flt4, Fms, Axl, c-Raf and DDR2. Phase 1.	Cell Cycle	C19H19ClN6O4S3	Mesylate	N/A	CS(O)(=O)=O.ClC1=CC=CC(NC(=O)NC2=NC=C(CCNC3=NC=NC4=C3SC=C4)S2)=C1 |c:7,23,25,29,33,t:5,14,16,21|	3,539	4	3	6
S1155	S3I-201	43671943	c9	L3500-01	365,36	501919-59-1	STAT	73	200	<1		http://selleckchem.com/products/S31-201.html	S3I-201 shows potent inhibition of STAT3 DNA-binding activity with IC50 of 86 µM in cell-free assays, and low activity towards STAT1 and STAT5.	JAK/STAT	C16H15NO7S	free base	NSC 74859	CC1=CC=C(C=C1)S(=O)(=O)OCC(=O)NC1=CC=C(C(O)=O)C(O)=C1 |c:3,5,24,t:1,16,18|	1,712	3	2	6
S1157	Delanzomib (CEP-18770)	43888220	d9	L3500-01	413,28	847499-27-8	Proteasome	83	201	<1		http://selleckchem.com/products/cep-18770.html	Delanzomib (CEP- 18770) is an orally active inhibitor of the chymotrypsin-like activity of proteasome with IC50 of 3.8 nM, with only marginal inhibition of the tryptic and peptidylglutamyl activities of the proteosome. Phase 1/2.	Proteases	C21H28BN3O5	free base	N/A	CC(C)[C@H](CNC(=O)[C@@H](NC(=O)C1=NC(=CC=C1)C1=CC=CC=C1)[C@@H](C)O)B(O)O |r,c:14,16,21,23,t:12,19|	2,646	3	3	9
S1159	Ganetespib (STA-9090)	44328685	e9	L3500-01	364,4	888216-25-9	HSP (e.g. HSP90)	40	110	<1		http://selleckchem.com/products/ganetespib-sta-9090.html	Ganetespib (STA-9090) is an HSP90 inhibitor with IC50 of 4 nM in OSA 8 cells, induces apoptosis of OSA cells while normal osteoblasts are not affected active metabolite of STA-1474. Phase 3.	Cytoskeletal Signaling	C20H20N4O3	free base	N/A	CC(C)C1=C(O)C=C(O)C(=C1)C1=NNC(=O)N1C1=CC2=C(C=C1)N(C)C=C2 |c:3,9,21,23,28,t:6,12,19|	3,866	2	3	3
S1173	WZ4002	44339908	f9	L3500-01	494,18	1213269-23-8	EGFR	13	26	<1		http://selleckchem.com/products/WZ4	WZ4002 is a novel, mutant-selective EGFR inhibitor for EGFR(L858R)/(T790M) with IC50 of 2 nM/8 nM in BaF3 cell line does not inhibit ERBB2 phosphorylation (T798I).	Protein Tyrosine Kinase	C25H27ClN6O3	free base	N/A	COC1=C(NC2=NC=C(Cl)C(OC3=CC(NC(=O)C=C)=CC=C3)=N2)C=CC(=C1)N1CCN(C)CC1 |c:2,19,21,23,26,28,t:5,7,1	4,807	5	2	8

												002.html						2|				
S1174	Ostarine (GTx-024, MK-2866)	42523433	g9	L3500-01	389,33	841205-47-8	Androgen Receptor	78	200	<1		http://selleckchem.com/products/Ostarine.html	MK-2866 (GTx-024) is a selective androgen receptor modulator (SARM) with Ki of 3.8 nM, and is tissue-selective for anabolic organs. Phase 3.	Endocrinology & Hormones	C19H14F3N3O3	free base	Enobosarm, ostarine	C[C@](O)(COC1=CC=C(C=C1)C#N)C(=O)NC1=CC=C(C#N)C(=C1)C(F)(F)F |r,c:7,9,23,t:5,17,19|	3,057	2	1	6
S1180	XAV-939	43911914	b10	L3500-01	312,31	284028-89-3	Wnt/beta-catenin	12	38	<1		http://selleckchem.com/products/XAV-939.html	XAV-939 selectively inhibits Wnt/β-catenin- mediated transcription through tankyrase1/2 inhibition with IC50 of 11 nM/4 nM in cell-free assays, regulates axin levels and does not affect CRE, NF-κB or TGF-β.	Stem Cells & Wnt	C14H11F3N2OS	free base	NVP-XAV939	OC1=C2CSCCC2=NC(=N1)C1=CC=C(C=C1)C(F)(F)F |c:1,8,10,15,17,t:13|	3,676	2	1	2
S1186	BIBR 1532	44336696	c10	L3500-01	331,36	321674-73-1	Telomerase	66	199	<1		http://selleckchem.com/products/BIBR1532.html	BIBR 1532 is a potent, selective, non- competitive telomerase inhibitor with IC50 of 100 nM in a cell-free assay. No inhibition of DNA and RNA polymerases, including HIV reverse transcriptase were observed at concentrations vastly exceeding the IC50 for telomerase.	DNA Damage	C21H17NO3	free base	N/A	CC(=C/C(=O)NC1=C(C=CC=C1)C(O)=O)\C1=CC2=CC=CC=C2C=C1 |c:8,10,20,22,25,t:6,16,18|	4,067	2	1	4
S1188	Anastrozole	44074811	d10	L3500-01	293,37	120511-73-1	Aromatase	59	201	<1		http://selleckchem.com/products/Anastrozole.html	Anastrozole is a third- generation nonsteroidal selective aromatase inhibitor. It may offer greater selectivity compared with other aromatase inhibitors, being without any intrinsic endocrine effects and with no apparent effect on the synthesis of adrenal steroids.	Endocrinology & Hormones	C17H19N5	free base	ZD-1033	CC(C)(C#N)C1=CC(=CC(CN2C=NC=N2)=C1)C(C)(C)C#N |c:7,12,14,16,t:5|	2,965	2	0	4
S1189	Aprepitant	44335519	e10	L3500-01	534,43	170729-80-3	Substance P	107	200	<1		http://selleckchem.com/products/Aprepitant.html	Aprepitant is a potent and selective neurokinin- 1 receptor antagonist with IC50 of 0.1 nM.	Others	C23H21F7N4O3	free base	MK-0869, L-754030	C[C@@H](O[C@H]1OCCN(CC2=NNC(=O)N2)[C@H]1C1=CC=C(F)C=C1)C1=CC(=CC(=C1)C(F)(F)F)C(F)(F)F |r,c:23,28,30,t:9,18,20,26|	4,635	4	2	8
S1191	Fulvestrant	43926657	f10	L3500-01	606,77	129453-61-8	trogen/progestogen Recep	100	165	<1		http://selleckchem.com/products/Fulvestrant.html	Fulvestrant is an estrogen receptor (ER) antagonist with IC50 of 0.94 nM in a cell-free assay.	Endocrinology & Hormones	C32H47F5O3S	free base	ICI-182780, ZD9238	[H][C@@]12CC[C@H](O)[C@@]1(C)CC[C@]1([H])C3=C(C[C@@H](CCCCCCCCCS(=O)CCCC(F)(F)C(F)(F)F)[C@@]21[H])C=C(O)C=C3 |r,c:13,45,t:42|	8,437	1	2	15

S1195	TAK-700 (Orteronel)	44126579	g10	L3500-01	307,35	426219-18-3	P450 (e.g. CYP17)	61	198	<1		http://selleckchem.com/products/TAK-700.html	TAK-700 (Orteronel) is a potent and highly selective human 17,20- lyase inhibitor with IC50 of 38 nM, exhibits >1000- fold selectivity over other CYPs (e.g. 11- hydroxylase and CYP3A4). Phase 3.	Metabolism	C18H17N3O2	free base	N/A	CNC(=O)C1=CC=C2C=C(C=CC2=C1)C1(O)CCN2C=NC=C12 |c:8,10,13,21,t:4,6,23|	1,075	2	1	2
S1196	Exemestane	44088294	b11	L3500-01	296,4	107868-30-4	Aromatase	54	182	<1		http://selleckchem.com/products/Exemestane.html	Exemestane is an aromatase inhibitor, inhibits human placental and rat ovarian aromatase with IC50 of 30 nM and 40 nM, respectively.	Endocrinology & Hormones	C20H24O2	free base	FCE24304, PNU155971	[H][C@@]12CCC(=O)[C@@]1(C)CC[C@@]1([H])[C@@]2([H])CC(=C)C2=CC(=O)C=C[C@]12C |r,c:23,t:19|	3,606	2	0	0
S1197	Finasteride	44050072	c11	L3500-01	372,54	98319-26-7	5-alpha Reductase	75	201	<1		http://selleckchem.com/products/Finasteride.html	Finasteride is a potent, reversible inhibitor of the rat type 1 5 alpha-reductase with Ki of 10.2 nM, used in the treatment of benign prostatic hyperplasia (BPH) and male pattern baldness (MPB).	Endocrinology & Hormones	C23H36N2O2	free base	MK-906	[H][C@@]12CC[C@H](C(=O)NC(C)(C)C)[C@@]1(C)CC[C@@]1([H])[C@@]2([H])CC[C@@]2([H])NC(=O) C=C[C@]12C |r,c:29|	3,174	2	2	2
S1202	Dutasteride	44131746	d11	L3500-01	528,53	164656-23-9	5-alpha Reductase	62	117	<1		http://selleckchem.com/products/Dutasteride.html	Dutasteride is a dual 5-α reductase inhibitor that inhibits conversion of testosterone to dihydrotestosterone (DHT).	Endocrinology & Hormones	C27H30F6N2O2	free base	GI198745, GG-745	[H][C@@]12CC[C@H](C(=O)NC3=CC(=CC=C3C(F)(F)F)C(F)(F)F)[C@@]1(C)CC[C@@]1([H])[C@@]2([H])CC[C@@]2([H])NC(=O)C=C[C@]12C |r,c:10,12,40,t:8|	5,703	2	2	4
S1210	Methotrexate	44341937	e11	L3500-01	454,44	59-05-2	DHFR	90	198	<1		http://selleckchem.com/products/Abitrexate.html	Methotrexate (MTX), analog of folic acid, is a nonspecific inhibitor of the dihydrofolate reductase(DHFR) of bacteria and cancerous cells as well as normal cells. It forms an inactive ternary complex with DHFR and NADPH.	Metabolism	C20H22N8O5	free base	NCI-C04671	CN(CC1=NC2=C(N)N=C(N)N=C2N=C1)C1=CC=C(C=C1)C(=O)N[C@@H](CCC(O)=O)C(O)=O |r,c:5,11,14,19,21,t:3,8,17|	0,114	7	3	9
S1216	PFI-1 (PF-6405761)	44061307	f11	L3500-01	347,39	1403764-72-6	Epigenetic Reader Domain	69	199	<1		http://selleckchem.com/products/pfl-1.html	PFI-1 is a highly selective BET (bromodomain-containing protein) inhibitor for BRD4 with IC50 of 0.22 μM and for BRD2 with IC50 of 98 nM in a cell- free assay.	Epigenetics	C16H17N3O4S	free base	N/A	COC1=C(C=CC=C1)S(=O)(=O)NC1=CC=C2NC(=O)N(C)CC2=C1 |c:4,6,24,t:2,13,15|	1,376	4	2	4
S1233	2-Methoxyestradiol (2-MeOE2)	43897322	g11	L3500-01	302,41	362-07-2	HIF	60	198	<1		http://selleckchem.com/products/2-Methoxyestradiol(2ME2). html	2-Methoxyestradiol (2- MeOE2) depolymerizes microtubules and blocks HIF-1α nuclear accumulation and HIF-transcriptional activity. Phase 2.	Angiogenesis	C19H26O3	free base	NSC 659853	[H][C@@]12CC[C@H](O)[C@@]1(C)CC[C@]1([H])C3=CC(OC)=C(O)C=C3CC[C@@]21[H] |r,c:20,t:15,17|	3,821	1	2	1

S1626	Naproxen Sodium	44140933	b2	L3500-03	252,24	26159-34-2	COX	3	12	50	198	http://selleckchem.com/products/Naproxen-Sodium(Aleve).html	Naproxen Sodium is a COX inhibitor for COX-1 and COX-2 with IC50 of 8.7 μM and 5.2 μM, respectively.	Neuronal Signaling	C14H13NaO3	Sodium Salt	RS-3650	[Na+].COC1=CC=C2C=C(C=CC2=C1)[C@H](C)C([O-])=O |r,c:6,8,11,t:2,4|	1,375	1	0	3
S1657	Enalaprilat Dihydrate	43927739	c2	L3500-03	348,4	84680-54-6	RAAS	70	201	<1		http://selleckchem.com/products/Enalaprilat.html	Enalaprilat is an angiotensin-converting enzyme (ACE) inhibitor with IC50 of 1.94 nM.	Endocrinology & Hormones	C18H28N2O7	Dihydrate	MK-422 Dihydrate	O.O.C[C@H](N[C@@H](CCC1=CC=CC=C1)C(O)=O)C(=O)N1CCC[C @H]1C(O)=O |r,c:8,10,t:6|	-1,53	3	1	8
S1738	Telmisartan	44348419	d2	L3500-03	514,62	144701-48-4	RAAS	13	25	<1		http://selleckchem.com/products/Telmisartan(Micardis).html	Telmisartan is an angiotensin II receptor antagonist (ARB) used in the management of hypertension.	Endocrinology & Hormones	C33H30N4O2	free base	BIBR 277	CCCC1=NC2=C(C=C(C=C2C)C2=NC3=C(C=CC=C3)N2C)N1CC1=CC=C(C=C1)C1=CC=CC=C1C(O)=O |c:7,9,17,19,30,32,37,39,t:3,5,13,15, 28,35|	7,799	3	0	7
S1776	Toremifene Citrate	43946954	e2	L3500-03	598,08	89778-27-8	trogen/progestogen Recep	100	167	<1		http://selleckchem.com/products/Toremifene-Citrate(Fareston).html	Toremifene Citrate is an oral selective estrogen receptor modulator (SERM), used in the treatment of advanced breast cancer.	Endocrinology & Hormones	C32H36ClNO8	Citrate	NSC 613680	OC(=O)CC(O)(CC(O)=O)C(O)=O.CN(C)CCOC1=CC=C(C=C1)C(=C(\CCCl)C1=CC=CC=C1)\C1=CC=CC= C1 |c:20,22,32,34,39,41,t:18,30,37|	1,945	4	0	14
S1801	Ranitidine Hydrochloride	44045244	f2	L3500-03	350,86	66357-59-3	Histamine Receptor	70	200	70	200	http://selleckchem.com/products/Ranitidine-hydrochloride(Zantac).html	Ranitidine is a histamine H2-receptor antagonist, used to treat stomach or intestinal ulcers.	Neuronal Signaling	C13H23ClN4O3S	Hydrochloride	AH19065	Cl.CN\C(NCCSCC1=CC=C(CN(C)C)O1)=C/[N+]([O-])=O |t:8,10|	1,421	1	2	10
S1802	AICAR (Acadesine)	44087818	g2	L3500-03	258,23	2627-69-2	AMPK	51	197	<1		http://selleckchem.com/products/Acadesine.html	AICAR (Acadesine), an AMPK activator, results in accumulation of ZMP, which mimics the stimulating effect of AMP on AMPK and AMPK kinase. Phase 3.	PI3K/Akt/mTOR	C9H14N4O5	free base	NSC105823	NC(=O)C1=C(N)N(C=N1)[C@@H]1O[C@H](CO)[C@@H](O)[C@H]1O |r,c:3,7|	-2,913	3	5	3
S1831	Carvedilol	44051317	b3	L3500-03	406,47	72956-09-3	Adrenergic Receptor	81	199	<1		http://selleckchem.com/products/carvedilol.html	Carvedilol is a non- selective beta blocker/alpha-1 blocker, used to treat congestive heart failure (CHF) and high blood pressure.	Neuronal Signaling	C24H26N2O4	free base	BM-14190, SKF 105517	COC1=CC=CC=C1OCCNCC(O)COC1=C2C(NC3=C2C=CC=C3)=CC= C1 |c:4,6,22,25,27,29,31,t:2,18|	4,014	3	3	10
S1847	Clemastine Fumarate	44130933	c3	L3500-03	459,96	14976-57-9	Histamine Receptor	35	76	<1		http://selleckchem.com/products/Clemastine-Fumarate.html	Clemastine Fumarate (Clemastine) is a selective histamine H1 receptor antagonist with IC50 of 3 nM.	Neuronal Signaling	C25H30ClNO5	Fumarate	N/A	OC(=O)\C=C\C(O)=O.CN1CCC[C@@H]1CCO[C@](C)(C1=CC=CC=C1)C1=CC=C(Cl)C=C1 |r,c:21,23,31,t:19,26,28|	1,81	3	0	8
S1880	Roxatidine Acetate HCl	44058134	d3	L3500-03	384,9	93793-83-0	Histamine Receptor	77	200	77	200	http://selleckchem.com/products/roxa	Roxatidine Acetate HCl is a specific and competitive histamin H2- receptor antagonist, with IC50 of 3.2 μM, inhibits gastric acid secretion and ulcer formation.	Neuronal Signaling	C19H29ClN2O4	Hydrochloride	N/A	Cl.CC(=O)OCC(=O)NCCCOC1=CC=CC(CN2CCCCC2)=C1 |c:14,24,t:12|	2,148	4	1	10

												tidine-acetate-hcl.html										
S1885	Felodipine	44045279	e3	L3500-03	384,25	72509-76-3	Calcium Channel	77	200	<1		http://selleckchem.com/products/Felodipine(Plendi l).html	Felodipine is a selective L-type Ca2+ channel blocker with IC50 of 0.15 nM.	Transmembrane Transporters	C18H19Cl2NO4	free base	CGH-869	CCOC(=O)C1=C(C)NC(C)=C(C1C1=CC=CC(Cl)=C1Cl)C(=O)OC |c:5,10,16,19,t:14|	3,549	4	1	6
S1905	Amlodipine	44164229	f3	L3500-03	408,88	88150-42-9	Calcium Channel	82	201	<1		http://selleckchem.com/products/Amlodipine(Norv asc).html	Amlodipine is a long- acting calcium channel blocker, used to lower blood pressure and prevent chest pain.	Transmembrane Transporters	C20H25ClN2O5	free base	UK-48340	CCOC(=O)C1=C(COCCN)NC(C)=C(C1C1=C(Cl)C=CC=C1)C(=O)OC |c:5,14,18,21,23|	1,576	5	2	10
S1909	Fluvastatin Sodium	43943657	g3	L3500-03	433,45	93957-55-2	HMG-CoA Reductase	87	201	1	2	http://selleckchem.com/products/Fluvastatin-Sodium(Lescol).html	Fluvastatin Sodium inhibits HMG-CoA reductase activity with IC50 of 8 nM in a cell- free assay.	Metabolism	C24H25FNNaO4	Sodium	XU-62-320	[Na+].CC(C)N1C(\C=C\[C@@H](O)C[C@@H](O)CC([O-])=O)=C(C2=C1C=CC=C2)C1=CC= C(F)C=C1 |r,c:15,17,20,22,30,t:25,27|	2,77	0	2	8
S1913	Tropicamide	44184081	b4	L3500-03	284,35	1508-75-4	AChR	57	200	<1		http://selleckchem.com/products/tropicamide.html	Tropicamide is an anticholinergic and a muscarinic receptor subtype M4-preferring antagonist with IC50 of 8.0 nM.	Neuronal Signaling	C17H20N2O2	free base	N/A	CCN(CC1=CC=NC=C1)C(=O)C(CO)C1=CC=CC=C1 |c:6,8,18,20,t:4,16|	1,432	2	1	6
S1959	Tolfenamic Acid	44162976	c4	L3500-03	261,7	13710-19-5	COX	52	199	<1		http://selleckchem.com/products/tolfenamic-acid.html	Tolfenamic Acid is a COX-2 inhibitor with IC50 of 0.2 μM.	Neuronal Signaling	C14H12ClNO2	free base	N/A	CC1=C(Cl)C=CC=C1NC1=C(C=CC=C1)C(O)=O |c:1,4,6,12,14,t:10|	4,16	1	1	3
S2001	Elvitegravir (GS-9137, JTK-303)	44168786	d4	L3500-03	447,88	697761-98-1	Integrase	89	199	<1		http://selleckchem.com/products/Elvitegravir.html	Elvitegravir (GS-9137, JTK-303) is an HIV integrase inhibitor for HIV-1 IIIB, HIV-2 EHO and HIV-2 ROD with IC50 of 0.7 nM, 2.8 nM and 1.4 nM in cell-free assays, respectively.	Microbiology	C23H23ClFNO5	free base	N/A	COC1=CC2=C(C=C1CC1=C(F)C(Cl)=CC=C1)C(=O)C(=CN2[C@H](CO)C(C)C)C(O)=O |r,c:4,6,10,14,16,21,t:2|	4,665	3	1	7
S2003	Maraviroc	44158802	e4	L3500-03	513,67	376348-65-1	CCR	100	195	<1		http://selleckchem.com/products/Maraviroc.html	Maraviroc is a CCR5 antagonist for MIP-1α, MIP-1β and RANTES with IC50 of 3.3 nM, 7.2 nM and 5.2 nM in cell- free assays, respectively.	Microbiology	C29H41F2N5O	free base	UK-427857	CC(C)C1=NN=C(C)N1C1CC2CCC(C1)N2CC[C@H](NC(=O)C1CCC(F)(F)CC1)C1=CC=CC=C1 |r,c:37,39,t:3,5,35|	3,921	3	1	8
S2005	Raltegravir (MK-0518)	44127912	f4	L3500-03	444,42	518048-05-0	Integrase	88	198	<1		http://selleckchem.com/products/Raltegravir-(MK-0518).html	a potent integrase (IN) inhibitor for WT and S217Q PFV IN with IC50 of 90 nM and 40 nM in cell-free assays, respectively. It shows greater than 1000-fold selectivity for HIV-1 IN over several related Mg2+-dependent enzyme such as HCV polymerase, HIV reverse transcriptase, HIV RNaseH and human α-,	Microbiology	C20H21FN6O5	free base	N/A	CN1C(=O)C(O)=C(N=C1C(C)(C)NC(=O)C1=NN=C(C)O1)C(=O)NCC1=CC=C(F)C=C1 |c:5,7,32,t:16,18,27,29|	-0,291	7	3	6

S2006	Pyrimethamine	43978706	g4	L3500-03	248,71	58-14-0	DHFR	10	40	<1		http://selleckchem.com/products/Pyrimethamine.h tml	Pyrimethamine is a dihydrofolate reductase (DHFR) inhibitor, used as an antimalarial drug.	Metabolism	C12H13ClN4	free base	N/A	CCC1=NC(N)=NC(N)=C1 C1=CC=C (Cl)C=C1 |c:5,8,16,t:2,11,13|	2,75	2	2	2
S2013	PF-573228	44136496	b5	L3500-03	491,49	869288-64-2	FAK	26	53	<1		http://selleckchem.com/products/pf-573228.html	PF-573228 is an ATP-competitive inhibitor of FAK with IC50 of 4 nM in a cell-free assay, ~50- to 250-fold selective for FAK than Pyk2, CDK1/7 and GSK-3β.	Angiogenesis	C22H20F3N5O3S	free base	N/A	CS(=O)(=O)C1=CC(CNC2=NC(NC3 =CC4=C(NC(=O)CC4)C =C3)=NC= C2C(F)(F)F)=CC=C1 |c:23,25,27,33,35,t:4,9,13,15|	3,967	5	3	7
S2018	ENMD-2076 L-(+)-Tartaric acid	43910464	c5	L3500-03	525,56	1291074-87-7	urora Kinase,FLT3,VEGF	100	190	<1		http://selleckchem.com/products/enmd-2076-l-tartaric-acid.html	ENMD-2076 L-(+)-Tartaric acid is the tartaric acid of ENMD- 2076, selective activity against Aurora A and Flt3 with IC50 of 14 nM and 1.86 nM, 25-fold more selective for Aurora A than Aurora B and less potent to VEGFR2/KDR and VEGFR3, FGFR1 and FGFR2 and PDGFRα. Phase 2.	Angiogenesis	C25H31N7O6	free base	N/A	O[C@H]([C@@H](O)C(O)=O)C(O) =O.CN1CCN(CC1)C1=N C(\C=C\C2 =CC=CC=C2)=NC(NC2= NNC(C)=C 2)=C1 |c:24,26,28,36,38,t:17,22,32|	-0,683	5	4	8
S2020	Formoterol Hemifumarate	43949271	d5	L3500-03	402,4	43229-80-7	Adrenergic Receptor	80	199	<1		http://selleckchem.com/products/formoterol-hemifumarate.html	Formoterol Hemifumarate is a potent, selective and long-acting β2- adrenoceptor agonist used in the management of asthma and chronic obstructive pulmonary disease(COPD).	Neuronal Signaling	C42H52N4O12	Hemifumarate	CGP 25827A, NSC 29958	OC(=O)\C=C\C(O)=O.COC1=CC=C (C[C@H](C)NC[C@@H](O)C2=CC =C(O)C(NC=O)=C2)C=C1.COC1=C C=C(C[C@H](C)NC[C@@H](O)C2 =CC=C(O)C(NC=O)=C2) C=C1 |r,c:28,31,54,57,t:9,11,20,22,35,37,4 6,48|	-1,526	6	8	18
S2026	Ginkgolide A	43656175	e5	L3500-03	408,4	15291-75-5	GABA Receptor	81	198	<1		http://selleckchem.com/products/ginkgolide-a.html	Ginkgolide A is an extract from in Ginkgo biloba and a g-aminobutyric acid (GABA) antagonist with a Ki of 14.5 μM.	Neuronal Signaling	C20H24O9	free base	BN52020	[H][C@]12CC34[C@@]5([H])C[C@@H](C(C)(C)C)C33[C@@H](O)C(=O)O[C@H]3O[C@@]4(C(=O)O5)[C@@]1(O)[C@H](C)C(=O)O2 |r|	-0,022	7	1	1
S2032	Rebamipide	44169142	f5	L3500-03	370,79	90098-04-7	Others	74	200	<1		http://selleckchem.com/products/rebamipide.html	Rebamipide is a cholecystokinin type 1 (CCK1) receptor inhibitor for inhibiting [125I]BH- CCK-8S with IC50 of 37.7 μM.	Others	C19H15ClN2O4	free base	OPC-12759	OC(=O)C(CC1=CC(=O)NC2=C1C= CC=C2)NC(=O)C1=CC= C(Cl)C=C1 |c:10,13,15,26,t:5,21,23|	2,606	3	2	5
S2042	Cyproterone Acetate	44356271	g5	L3500-03	416,94	427-51-0	Androgen Receptor	83	199	<1		http://selleckchem.com/products/cyproterone-acetate.html	Cyproterone acetate is an androgen receptor (AR) antagonist with IC50 of 7.1 nM, as well as a weak progesterone receptor agonist with weak pro-gestational and glucocorticoid activity.	Endocrinology & Hormones	C24H29ClO4	free base	N/A	[H][C@@]12CC[C@](OC(C)=O)(C(C)=O)[C@@]1(C)CC[C@@]1([H])[C@@]2([H])C=C(Cl)C2=CC(=O)[C@@H]3C[C@@H]3[C@]12C |r,t:22,25|	3,314	4	0	3
S2043	Memantine HCl	43906391	b6	L3500-03	215,76	41100-52-1	PA	43	199	30	139	http://selleck	Memantine HCl is a CYP2B6 and CYP2D6	Neuronal	C12H22ClN	Hydrochl	N/A		2,289	0	1	0

							Receptor-kainate Rec					chem.com/products/memantine-hydrochloride-namenda.html	inhibitor for recombinant CYP2B6 and CYP2D6 with Ki of 0.51 nM and 94.9 µM, respectively.	Signaling		oride		Cl.CC12CC3CC(C)(C1)CC(N)(C3)C 2				
S2051	Captopril	43946587	c6	L3500-03	217,29	62571-86-2	RAAS	43	198	2	9	http://selleckchem.com/products/captopril-capoten.html	Captopril is an angiotensin-converting enzyme (ACE) inhibitor with IC50 of 6 nM.	Endocrinology & Hormones	C9H15NO3S	free base	SQ 14225	C[C@H](CS)C(=O)N1CCC[C@H]1 C(O)=O |r|	0,667	2	0	3
S2061	Lovastatin	44135419	d6	L3500-03	404,54	75330-75-5	HMG-CoA Reductase	8	20	<1		http://selleckchem.com/products/lovastatin-mevacor.html	Lovastatin is an inhibitor of HMG-CoA reductase with IC50 of 3.4 nM in a cell-free assay, used for lowering cholesterol (hypolipidemic agent).	Metabolism	C24H36O5	free base	MK-803	[H][C@]12[C@H](C[C@@H](C)C=C1C=C[C@H](C)[C@@H]2CC[C@@H]1C[C@@H](O)CC(=O)O1)OC(=O)[C@@H](C)CC |r,c:6,9|	4,218	4	1	7
S2065	Lafutidine	43909646	e6	L3500-03	431,55	118288-08-7	Histamine Receptor	86	199	<1		http://selleckchem.com/products/lafutidine.html	Lafutidine, a newly developed histamine H(2)-receptor antagonist, inhibits gastric acid secretion.	Neuronal Signaling	C22H29N3O4S	free base	FRG-8813	O=C(CS(=O)CC1=CC=CO1)NC\C=C/COC1=CC(CN2CCCCC2)=CC=N 1 |c:8,28,30,t:6,18|	1,987	5	1	11
S2077	Atorvastatin Calcium	44135100	f6	L3500-03	1155,34	134523-03-8	HMG-CoA Reductase	100	87	<1		http://selleckchem.com/products/atorvastatin-calcium-lipitor.html	Atorvastatin Calcium is an inhibitor of HMG-CoA reductase used as a cholesterol-lowering medication that blocks the production of cholesterol.	Metabolism	C66H68CaF2N4O10	Calcium	N/A	[Ca++].CC(C)C1=C(C(=O)NC2=CC=CC=C2)C(=C(N1CC[C@@H](O)C[C@@H](O)CC([O-])=O)C1=CC=C(F)C=C1)C1=CC=CC=C1.CC(C)C1=C(C(=O)NC2=CC=CC=C2)C(=C(N1CC[C@@H](O)C[C@@H](O)CC([O-])=O)C1=CC=C(F)C=C1)C1=CC=C C=C1 |r,c:3,10,12,15,35,40,42,47,54,56,59,70,84,86,t:8,30,32,38,52,74,76,82|	8,163	2	6	24
S2085	Trimebutine	43950464	g6	L3500-03	387,47	39133-31-8	Opioid Receptor	50	129	<1		http://selleckchem.com/products/trimebutine.html	Trimebutine is an agonist of peripheral mu, kappa and delta opiate receptors, used as spasmolytic agent for treatment of both acute and chronic abdominal pain.	Neuronal Signaling	C22H29NO5	free base	Mebutin	CCC(COC(=O)C1=CC(OC)=C(OC)C(OC)=C1)(N(C)C)C1=CC=CC=C1 |c:17,25,27,t:7,11,23|	4,115	5	0	10
S2102	Rasagiline Mesylate	43948703	b7	L3500-03	267,34	161735-79-1	MAO	53	198	53	198	http://selleckchem.com/products/rasagiline-mesylate.html	Rasagiline Mesylate is a new MAO-B inhibitor for the treatment of idiopathic Parkinson's disease.	Metabolism	C13H17NO3S	Mesylate	TVP-1012	CS(O)(=O)=O.C#CCN[C@@H]1CCC2=CC=CC=C12 |r,c:13,t:11,15|	2,705	0	1	2
S2103	Naltrexone HCl	43949096	c7	L3500-03	377,86	16676-29-2	Opioid Receptor	14	37	14	37	http://selleckchem.com/products/naltrexone-hcl.html	Naltrexone HCl is an opioid receptor antagonist used primarily in the management of alcohol dependence and opioid dependence.	Neuronal Signaling	C20H24ClNO4	Hydrochloride	N/A	Cl.OC1=CC=C2C[C@H]3N(CC4CC4)CC[C@@]45[C@@H](OC1=C24)C(=O)CC[C@@]35O |r,c:19,t:1,3|	2,039	2	1	2

<table>
<tr><th></th><th></th><th></th><th></th><th></th><th></th><th></th><th></th><th></th><th></th><th></th><th></th><th></th><th></th><th></th><th></th><th></th><th></th><th></th><th></th><th></th><th></th><th></th></tr>
<tr><td>S2104</td><td>Levosulpiride</td><td>44133891</td><td>d7</td><td>L3500-03</td><td>341,43</td><td>23672-07-3</td><td>Dopamine Receptor</td><td>69</td><td>202</td><td><1</td><td></td><td>http://selleckchem.com/products/levosulpiride-levogastrol.html</td><td>Levosulpiride is a selective antagonist for D2 dopamine receptors used as an antipsychotic and prokinetic agent.</td><td>Neuronal Signaling</td><td>C15H23N3O4S</td><td>free base</td><td>N/A</td><td>CCN1CCC[C@H]1CNC(=O)C1=C(OC)C=CC(=C1)S(N)(=O)=O |r,c:12,16,18|</td><td>0,704</td><td>4</td><td>2</td><td>6</td></tr>
<tr><td>S2109</td><td>Imidapril HCl</td><td>43942365</td><td>e7</td><td>L3500-03</td><td>441,91</td><td>89396-94-1</td><td>RAAS</td><td>88</td><td>199</td><td>54</td><td>122</td><td>http://selleckchem.com/products/imidapril-tanatril.html</td><td>Imidapril HCl is an angiotensin-converting enzyme (ACE) inhibitor with IC50 of 2.6 nM, used for the treatment of hypertension.</td><td>Endocrinology & Hormones</td><td>C20H27N3O6</td><td>Hydrochloride</td><td>N/A</td><td>CCOC(=O)[C@H](CCC1=CC=CC=C1)N[C@@H](C)C(=O)N1[C@@H](CN(C)C1=O)C(O)=O |r,c:10,12,t:8|</td><td>-1,197</td><td>5</td><td>1</td><td>10</td></tr>
<tr><td>S2149</td><td>GSK1292263</td><td>42370968</td><td>f7</td><td>L3500-03</td><td>456,56</td><td>1032823-75-8</td><td>GPR</td><td>34</td><td>74</td><td><1</td><td></td><td>http://selleckchem.com/products/GSK1292263.ht ml</td><td>GSK1292263 is a novel GPR119 agonist, showing potential for the treatment of type 2 diabetes. Phase 2.</td><td>Endocrinology & Hormones</td><td>C23H28N4O4S</td><td>free base</td><td>N/A</td><td>CC(C)C1=NOC(=N1)N1CCC(COC2=CN=C(C=C2)C2=CC=C(C=C2)S(C)(=O)=O)CC1 |c:6,17,19,24,26,t:3,15,22|</td><td>3,893</td><td>7</td><td>0</td><td>7</td></tr>
<tr><td>S2151</td><td>Sonidegib (Erismodegib, NVP-LDE225)</td><td>44199015</td><td>g7</td><td>L3500-03</td><td>485,5</td><td>956697-53-3</td><td>Hedgehog/Smoothened</td><td>97</td><td>200</td><td><1</td><td></td><td>http://selleckchem.com/products/LDE225(NVP-LDE225).html</td><td>Sonidegib (Erismodegib, NVP-LDE225) is a Smoothened (Smo) antagonist, inhibiting Hedgehog (Hh) signaling with IC50 of 1.3 nM (mouse) and 2.5 nM (human) in cell-free assays, respectively. Phase 3.</td><td>Stem Cells & Wnt</td><td>C26H26F3N3O3</td><td>free base</td><td></td><td>C[C@H]1CN(C[C@@H](C)O1)C1=CC=C(NC(=O)C2=C(C)C(=CC=C2)C2=CC=C(OC(F)(F)F)C=C2)C=N1 |r,c:16,19,21,33,36,t:9,11,24,26|</td><td>6,732</td><td>4</td><td>1</td><td>6</td></tr>
<tr><td>S2153</td><td>CGS 21680 HCl</td><td>42396473</td><td>b8</td><td>L3500-03</td><td>535,98</td><td>124431-80-7</td><td>Adenosine Receptor</td><td>100</td><td>187</td><td><1</td><td></td><td>http://selleckchem.com/products/CGS-21680-hydrochloride.html</td><td>CGS 21680 HCl is an adenosine A2 receptor agonist with IC50 of 22 nM, exhibits 140-fold over A1 receptor.</td><td>GPCR & G Protein</td><td>C23H30ClN7O6</td><td>Hydrochloride</td><td>N/A</td><td>Cl.CCNC(=O)[C@H]1O[C@H]([C@H](O)[C@@H]1O)N1C=NC2=C1N=C(NCCC1=CC=C(CCC(O)=O)C=C1)N=C2N |r,c:14,16,33,36,t:19,24,26|</td><td>0,911</td><td>6</td><td>5</td><td>10</td></tr>
<tr><td>S2163</td><td>PF-4708671</td><td>43904409</td><td>c8</td><td>L3500-03</td><td>390,41</td><td>1255517-76-0</td><td>S6 Kinase</td><td>30</td><td>77</td><td><1</td><td></td><td>http://selleckchem.com/products/pf-4708671.html</td><td>PF-4708671 is a cell- permeable inhibitor of p70 ribosomal S6 kinase (S6K1 isoform) with Ki/IC50 of 20 nM/160 nM in cell-free assays, 400- fold greater selectivity for S6K1 than S6K2, and 4- and >20-fold selectivity for S6K1 than MSK1 and RSK1/2, respectively. First S6K1-specific inhibitor to be reported.</td><td>PI3K/Akt/mTOR</td><td>C19H21F3N6</td><td>free base</td><td>N/A</td><td>CCC1=CN=CN=C1N1CCN(CC2=NC3=C(N2)C=CC(=C3)C(F)(F)F)CC1 |c:4,6,16,20,22,t:2,14|</td><td>3,886</td><td>3</td><td>1</td><td>5</td></tr>
<tr><td>S2168</td><td>PD128907 HCl</td><td>43453455</td><td>d8</td><td>L3500-03</td><td>285,77</td><td>112960-16-4</td><td>Dopamine Receptor</td><td>12</td><td>42</td><td>50</td><td>175</td><td>http://selleckchem.com/products/PD-128907.html</td><td>PD 128907 HCl is a potent and selective dopamine D3 receptor agonist, with EC50 of 0.64 nM, exhibits 53-fold selectivity over dopamine D2 receptor.</td><td>Neuronal Signaling</td><td>C14H20ClNO3</td><td>Hydrochloride</td><td>N/A</td><td>Cl.[H][C@]12COC3=CC=C(O)C=C3[C@]1([H])OCCN2CCC |r,c:9,t:4,6|</td><td>2,463</td><td>2</td><td>1</td><td>2</td></tr>
<tr><td>S2178</td><td>AG-14361</td><td>44359424</td><td>e8</td><td>L3500-03</td><td>320,39</td><td>328543-09-5</td><td>PARP</td><td>12</td><td>37</td><td><1</td><td></td><td>http://selleckchem.com/p</td><td>AG14361 is a potent inhibitor of PARP1 with Ki of <5 nM in a cell-free assay. It is at least 1000- fold more potent than the benzamides.</td><td>DNA Damage</td><td>C19H20N4O</td><td>free base</td><td>N/A</td><td>CN(C)CC1=CC=C(C=C1)C1=NC2=</td><td>2,576</td><td>2</td><td>1</td><td>3</td></tr>
</table>

												roducts/AG14361.html						CC=CC3=C2N1CCNC3=O |c:6,8,15,17,t:4,11,13|				
S2179	Gandotinib (LY2784544)	43906781	f8	L3500-03	469,94	1229236-86-5	JAK	94	200	<1		http://selleckchem.com/products/LY2784544.html	Gandotinib (LY2784544) is a potent JAK2 inhibitor with IC50 of 3 nM, effective in JAK2V617F, 8- and 20-fold selective versus JAK1 and JAK3. Phase 2.	JAK/STAT	C23H25ClFN7O	free base	N/A	CC1=NNC(NC2=NN3C(CC4=CC=C(Cl)C=C4F)=C(C)N=C3C(CN3CCOCC3)=C2)=C1 |c:6,16,22,33,35,t:1,6,11,13,19|	3,938	4	2	6
S2193	GSK461364	43898877	g8	L3500-03	543,6	929095-18-1	PLK	10	18	<1		http://selleckchem.com/products/GSK461364.html	GSK461364 inhibits purified Plk1 with Ki of 2.2 nM in a cell-free assay. It is more than 1000-fold selective against Plk2/3. Phase 1.	Cell Cycle	C27H28F3N5O2S	free base	N/A	C[C@@H](OC1=C(SC(=C1)N1C=NC2=C1C=C(CN1CCN(C)CC1)C=C2)C(N)=O)C1=CC=CC=C1C(F)(F)F |r,c:6,10,12,26,34,36,t:3,15,32|	5,125	3	1	8
S2198	SGI-1776 free base	44190247	b9	L3500-03	405,42	1025065-69-3	Pim	81	200	<1		http://selleckchem.com/products/SGI-1776.html	SGI-1776 free base is a novel ATP competitive inhibitor of Pim1 with IC50 of 7 nM in a cell-free assay, 50- and 10- fold selective versus Pim2 and Pim3, also potent to Flt3 and haspin. Phase 1.	JAK/STAT	C20H22F3N5O	free base	N/A	CN1CCC(CNC2=NN3C(C=C2)=NC=C3C2=CC=CC(OC(F)(F)F)=C2)CC 1 |c:11,13,15,20,27,t:7,18|	5,206	3	1	6
S2205	OSI-420	42369646	c9	L3500-03	415,87	183320-51-6	EGFR	83	200	<1		http://selleckchem.com/products/OSI-420-Desmethyl-Erlotinib,CP-473420.html	OSI-420 is the active metabolite of Erlotinib (EGFR inhibitor with IC50 of 2 nM).	Protein Tyrosine Kinase	C21H22ClN3O4	Hydrochloride	ethyl Erlotinib (CP-473420	Cl.COCCOC1=CC2=C(C=C1OCCO)C(NC1=CC=CC(=C1)C#C)=NC=N2 |c:7,9,20,22,26,28,t:5,18|	4,283	5	2	9
S2216	Mubritinib (TAK 165)	43962250	d9	L3500-03	468,47	366017-09-6	HER2	13	28	<1		http://selleckchem.com/products/Mubritinib-TAK-165.html	Mubritinib (TAK-165) is a potent inhibitor of HER2/ErbB2 with IC50 of 6 nM in BT-474 cell no activity to EGFR, FGFR, PDGFR, JAK1, Src and Blk in BT-474 cell line. Phase 1.	Protein Tyrosine Kinase	C25H23F3N4O2	free base	N/A	FC(F)(F)C1=CC=C(\C=C\C2=NC(COC3=CC=C(CCCCN4C=CN=N4)C=C3)=CO2)C=C1 |c:24,26,29,31,35,t:4,6,10,15,17|	5,844	5	0	11
S2217	Irinotecan HCl Trihydrate	43951285	e9	L3500-03	677,18	136572-09-3	Topoisomerase	100	148	1	1	http://selleckchem.com/products/Irinotecan-Hcl-Trihydrate-Campto.html	Irinotecan prevents DNA from unwinding by inhibition of topoisomerase 1.	DNA Damage	C33H45ClN4O9	HCl & trihydrat	CPT-11 HCl Trihydrate	O.O.O.Cl.CCC1=C2CN3C(=CC4=C(COC(=O)[C@]4(O)CC)C3=O)C2=NC2=CC=C(OC(=O)N3CCC(CC3)N3CCCCC3)C=C12 |r,c:2,6,23,t:8,25,27,46|	3,446	6	0	5
S2221	Apatinib?mesylate	44138608	f9	L3500-03	493,58	1218779-75-9	VEGFR	22	45	<1		http://selleckchem.com/products/Apatinib-YN968D1.html	Apatinib is an orally bioavailable, selective VEGFR2 inhibitor with IC50 of 1 nM.	Protein Tyrosine Kinase	C25H27N5O4S	Sulfate	YN968D1	CS(O)(=O)=O.O=C(NC1=CC=C(C=C1)C1(CCCC1)C#N)C1=CC=CN=C1NCC1=CC=NC=C1 |c:9,11,24,26,33,35,t:7,22,31|	3,164	3	2	6
S2222	PF-3716556	44416479	g9	L3500-03	394,47	928774-43-0	ATPase,Proton Pump	79	200	<1		http://selleck	and selective P-CAB (potassium-competitive acid blocker), with pIC50 of 6.026 and 7.095 for the inhibition of porcine H+,K+-ATPase activity in ion-leaky and ion-tight assay, respectively, inhibits gastric acid secretion, displays no activity at Na+,K+-ATPase, used for the treatment of gastroesophageal reflux	Transmembrane Transp	C22H26N4O3	Free Base	N/A	CN(CCO)C(=O)C1=CN2C	1,499	3	2	5

												chem.com/products/PF-3716556.html		orters				=C(C)N= C2C(N[C@@H]2CCOC3 =CC=CC(C)=C23)=C1				
S2228	Belnacasan (VX-765)	44113798	b10	L3500-03	509	273404-37-8	Caspase	100	196	<1		http://selleckchem.com/products/VX-765.html	Belnacasan (VX-765) is a potent and selective inhibitor of caspase-1 with Ki of 0.8 nM in a cell- free assay. Phase 2.	Apoptosis	C24H33ClN4O6	free base	N/A	CCO[C@@H]1OC(=O)C[C@@H]1 NC(=O)[C@@H]1CCCN 1C(=O)[C @@H](NC(=O)C1=CC(Cl)=C(N)C= C1)C(C)(C)C |r,c:31,t:25,28|	1,698	6	3	8
S2230	Galunisertib (LY2157299)	43880930	c10	L3500-03	369,42	700874-72-2	TGF-beta/Smad	74	200	<1		http://selleckchem.com/products/ly2157299.html	Galunisertib (LY2157299) is a potent TGFβ receptor I (TβRI) inhibitor with IC50 of 56 nM in a cell-free assay. Phase 2/3.	TGF-beta/Smad	C22H19N5O	free base	N/A	CC1=CC=CC(=N1)C1=N N2CCCC2 =C1C1=CC=NC2=C1C= C(C=C2)C(N)=O |c:3,5,15,20,22,25,27,t:1,8,18|	3,095	4	1	3
S2233	Esomeprazole sodium	43976369	d10	L3500-03	367,4	161796-78-7	ATPase	73	199	73	199	http://selleckchem.com/products/esomeprazole-sodium.html	Esomeprazole Sodium is a sodium salt of esomeprazole that is a potent proton pump inhibitor with an IC50 of 0.076 mg/kg.	Transmembrane Transporters	C17H18N3NaO3S	Sodium salt	N/A	COC1=CC=C2N([Na])C(=NC2=C1)[S@@](=O)CC1=NC=C(C)C(OC)=C 1C	3,008	5	0	5
S2239	Tubacin	43643540	e10	L3500-03	721,86	537049-40-4	HDAC	100	139	<1		http://selleckchem.com/products/Tubacin.html	Tubacin is a highly potent and selective, reversible, cell-permeable HDAC6 inhibitor with an IC50 of 4 nM in a cell-free assay, approximately 350-fold selectivity over HDAC1.	Cytoskeletal Signaling	C41H43N3O7S	Free Base	N/A	OCC1=CC=C(C=C1)[C@@H]1C[C @H](CSC2=NC(=C(O2)C2=CC=CC =C2)C2=CC=CC=C2)O[C@@H](O1)C1=CC=C(NC(=O)CCC CCCC(=O) NO)C=C1	7,181	6	3	16
S2262	Apigenin	43038898	f10	L3500-03	270,24	520-36-5	P450 (e.g. CYP17)	54	200	<1		http://selleckchem.com/products/Apigenin.html	Apigenin is a potent P450 inhibitor for CYP2C9 with Ki of 2 µM.	Metabolism	C15H10O5	free base	NSC 83244, LY 080400	OC1=CC=C(C=C1)C1=C C(=O)C2= C(O1)C=C(O)C=C2O |c:3,5,12,19,t:1,8,16|	2,41	2	3	1
S2270	Bergenin	43964667	g10	L3500-03	328,27	477-90-7	Others	66	201	<1		http://selleckchem.com/products/Bergenin-(Cuscutin).html	Bergenin is trihydroxybenzoic acid glycoside and the C-glycoside of 4-O-methyl gallic acid.	Others	C14H16O9	free base	Cuscutin	[H][C@]12OC(=O)C3=C(C(O)=C(O C)C(O)=C3)[C@]1([H])O[C@H](CO)[C@@H](O)[C@@H]2O |r,c:13,t:5,8|	-0,849	4	5	2
S2285	Cryptotanshinone	43933848	b11	L3500-03	296,36	35825-57-1	STAT	5	17	<1		http://selleckchem.com/products/Cryptotanshinon e.html	Cryptotanshinone is a STAT3 inhibitor with IC50 of 4.6 µM in a cell- free assay, strongly inhibits phosphorylation of STAT3 Tyr705, with a small effect on STAT3 Ser727, but none against STAT1 nor STAT5.	JAK/STAT	C19H20O3	free base	N/A	C[C@H]1COC2=C1C(=O)C(=O)C1 =C3CCCC(C)(C)C3=CC= C21 |r,c:4,11,20,t:22|	3,761	3	0	0
S2341	(-)-Parthenolide	44088983	c11	L3500-03	248,32	20554-84-1	HDAC,NF-κB,Mdm2,p53	49	197	<1		http://selleck	(-)-Parthenolide, an inhibitor of the Nuclear Factor-κB Pathway, specifically depletes HDAC1 protein without affecting other class I/II HDACs Also promotes the ubiquitination of MDM2 and activates p53 cellular functions.	NF-κB	C15H20O3	free base	N/A	CC1=CCC[C@@]2(C)O[	2,923	3	0	0

												chem.com/products/Parthenolide((-)-Parthenolide).html						C@H]2[C @H]2OC(=O)C(=C)[C@ @H]2CC1 |r|				
S2437	Rotundine	43452428	d11	L3500-03	355,43	483-14-7	Dopamine Receptor	8	23	<1		http://selleckchem.com/products/rotundine.html	Rotundine (L-tetrahydropalmatine, L- THP) is a selective dopamine D1 receptor antagonist with IC50 of 166 nM.	Neuronal Signaling	C21H25NO4	free base	N/A	[H][C@@]12CC3=CC=C(OC)C(OC)=C3CN1CCC1=C2C=C(OC)C(OC)= C1 |r,c:11,19,28,t:3,5,22|	3,599	4	0	4
S2449	Forskolin	44193380	e11	L3500-03	410,5	66575-29-9	cAMP	82	200	<1		http://selleckchem.com/products/forskolin.html	Forskolin is a ubiquitous activator of eukaryotic adenylyl cyclase (AC) in a wide variety of cell types, commonly used to raise levels of cAMP in the study and research of cell physiology.	GPCR & G Protein	C22H34O7	free base	Coleonol	[H][C@@]12[C@H](O)[C@H](OC(C)=O)[C@@]3(C)O[C@](C)(CC(=O)[C@]3(O)[C@@]1(C)[C@@H](O)C CC2(C)C)C=C |r|	0,837	4	2	3
S2454	Bupivacaine HCl	44183221	f11	L3500-03	324,89	18010-40-7	Sodium Channel	65	200	23	71	http://selleckchem.com/products/Bupivacaine-hydrochloride(Marcain).h tml	Bupivacaine HCl binds to the intracellular portion of voltage-gated sodium channels and blocks sodium influx into nerve cells, used for treating cardiac arrhythmias.	Transmembrane Transporters	C18H29ClN2O	Hydrochloride	N/A	Cl.CCCCN1CCCCC1C(=O)NC1=C(C)C=CC=C1C |c:14,17,19|	4,692	1	1	5
S2496	Ozagrel	43948969	g11	L3500-03	228,25	82571-53-7	P450 (e.g. CYP17)	46	202	<1		http://selleckchem.com/products/Ozagrel.html	Ozagrel is a selective thromboxane A(2) (TXA(2)) synthetase inhibitor with IC50 of 11 nM for rabbit platelet, used for the improvement of postoperative cerebrovascular contraction and accompanying cerebral ischaemia.	Metabolism	C13H12N2O2	free base	OKY-046	OC(=O)\C=C\C1=CC=C(CN2C=CN=C2)C=C1 |c:11,13,16,t:5,7|	1,604	2	0	4
S7414	Caffeic Acid Phenethyl Ester	43928825	b2	L3500-08	284,31	104594-70-9	NF-κB	57	200	<1		http://selleckchem.com/products/caffeic-acid-phenethyl-ester.html	Caffeic acid phenethyl ester is a potent and specific inhibitor of NF- κB activation, and also displays antioxidant, immunomodulatory and antiinflammatory activities.	NF-κB	C17H16O4	free base	APE, Phenylethyl Caffeat	OC1=CC=C(\C=C\C(=O)OCCC2=CC=CC=C2)C=C1O |c:14,16,19,t:1,3,12|	3,573	2	2	6
S7421	CGP 57380	43643696	c2	L3500-08	244,23	522629-08-9	MNK	48	197	<1		http://selleckchem.com/products/cgp57380.html	CGP 57380 is a potent MNK1 inhibitor with IC50 of 2.2 μM, exhibiting no inhibitory activity on p38, JNK1, ERK1 and -2, PKC, or c-Src-like kinases.	MAPK	C11H9FN6	free base	N/A	NC1=C2C(NC3=CC=C(F)C=C3)=N NC2=NC=N1 |c:1,10,12,16,18,t:5,7|	1,981	3	3	2
S7424	PD 151746	43474748	d2	L3500-08	237,25	179461-52-0	Cysteine Protease	47	198	<1		http://selleckchem.com/products/pd-151746.html	PD 151746 is a selective, cell-permeable calpain inhibitor with Ki of 0.26 μM for μ-Calpain, about 20-fold selectivity over m-calpain.	Proteases	C11H8FNO2S	free base	N/A	OC(=O)C(\S)=C\C1=CNC2=CC=C(F)C=C12 |t:6,9,11,14|	2,49	1	1	2

S7430	SB-3CT	44090853	e2	L3500-08	306,4	292605-14-2	MMP	61	199	<1		http://selleckchem.com/products/sb-3ct.html	SB-3CT is an effective and selective gelatinase inhibitor with Ki of 13.9 nM and 600 nM for MMP- 2 and MMP-9, respectively.	Proteases	C15H14O3S2	free base	N/A	O=S(=O)(CC1CS1)C1=CC=C(OC2=CC=CC=C2)C=C1 |c:15,17,20,t:8,10,13|	3,377	3	0	5
S7436	NH125	44090252	f2	L3500-08	524,56	278603-08-0	CaMK	100	191	<1		http://selleckchem.com/products/nh125.html	NH125 is a selective eEF 2 kinase inhibitor with IC50 of 60 nM, >125-fold selectivity over PKC, PKA, and CaMKII, and also a potent histidine kinase inhibitor.	Neuronal Signaling	C27H45IN2	iodide	N/A	[I-].CCCCCCCCCCCCCCCC[N+]1=C(C)N(CC2=CC=CC=C2)C=C1 |c:16,23,25,28,t:21|	6,032	0	0	17
S7437	Sal003	43466476	g2	L3500-08	463,21	1164470-53-4	phosphatase	93	201	<1		http://selleckchem.com/products/sal003.html	Sal003 is a potent and cell-permeable eIF-2α phosphatase inhibitor.	Others	C18H15Cl4N3OS	free base	N/A	ClC1=CC=C(C=C1)NC(=S)NC(C(Cl)(Cl)Cl)NC(=O)\C=C\C1=CC=CC=C1 |c:3,5,24,26,t:1,22|	6,298	1	3	8
S7445	E3330	43469573	b3	L3500-08	378,46	136164-66-4	DNA/RNA Synthesis	75	198	<1		http://selleckchem.com/products/e3330.html	E3330 is a potent and selective APE1(Ref-1) inhibitor, which suppressed NF-kappa B DNA-binding activity.	DNA Damage	C21H30O6	free base	N/A	CCCCCCCCC\C(=C/C1=C(C)C(=O)C(OC)=C(OC)C1=O)C(O)=O |c:11,t:18|	4,663	5	0	12
S7449	CRT0044876	43887457	c3	L3500-08	206,15	6960-45-8	DNA/RNA Synthesis	41	199	<1		http://selleckchem.com/products/crt0044876.html	CRT0044876 is a potent and selective APE1 inhibitor with IC50 of ~3 μM.	DNA Damage	C9H6N2O4	free base	NSC 69877,7-NO2-ICA	OC(=O)C1=CC2=C(N1)C(=CC=C2)[N+]([O-])=O |c:5,9,11,t:3|	1,939	1	1	2
S7457	XEN445	43127263	d3	L3500-08	366,33	1515856-92-4	Lipase	80	218	<1		http://selleckchem.com/products/xen445.html	XEN445 is a potent and selective endothelial lipase inhibitor with IC50 of 0.237 μM.	Metabolism	C18H17F3N2O3R	free base	N/A	[*].OC(=O)C1=CC(=CC=C1N1CC[C@@H](C1)OCC1=NC=CC=C1)C(F)(F)F |r,$(S)-2-(3-(pyridin-2- ylmethoxy)pyrrolidin-1-yl)-5-(trifluoromethyl)benzoic acid_R0;;;;;;;;;;;;;;;;;;;;;;;;;;$,c:5,7,20, 22,t:3,18|	3,725	3	0	6
S7460	BTB06584	43514837	e3	L3500-08	417,82	219793-45-0	ATPase	84	201	<1		http://selleckchem.com/products/btb06584.html	BTB06584 is an IF1-dependent, selective inhibitor of the mitochondrial F1 Fo-ATPase.	Transmembrane Transporters	C19H12ClNO6S	free base	N/A	[O-][N+](=O)C1=CC=C(C=C1OC(=O)C1=CC=C(Cl)C=C1)S(=O)(=O)C1=C C=CC=C1 |c:5,7,18,26,28,t:3,13,15,24|	4,923	4	0	6
S7461	LDC000067	42581412	f3	L3500-08	370,43	1073485-20-7	CDK	74	200	<1		http://selleckchem.com/products/ldc000067.html	LDC000067 is a highly selective CDK9 inhibitor with IC50 of 44 nM, 55/125/210/ >227/ >227-fold selectivity over CDK2/1/4/6/7.	Cell Cycle	C18H18N4O3S	free base	LDC067	COC1=CC=CC=C1C1=NC=NC(NC2=CC(CS(N)(=O)=O)=CC=C2)=C1 |c:4,6,11,22,24,26,t:2,9,15|	2,783	5	2	6
S7462	PI-1840	44084277	g3	L3500-08	394,47	1401223-22-0	Proteasome	78	198	<1		http://selleckchem.com/products/pi-1840.html	PI-1840 is a reversible and selective chymotrypsin-like (CT-L) inhibitor with IC50 of 27 nM with little effects on the other two major proteasome proteolytic activities, trypsin-like (T- L) and postglutamyl- peptide-hydrolysis-like (PGPH-L).	Proteases	C22H26N4O3	free base	N/A	CCCC1=CC=C(OCC(=O)N(CC2=NC(=NO2)C2=CC=CN=C2)C(C)C)C= C1 |c:15,21,23,29,t:3,5,13,19|	3,22	6	0	9
S7467	LB42708	43426837	b4	L3500-08	555,46	226929-39-1	Transferase	100	180	<1			LB42708 is an orally active farnesyltransferase (FTase) inhibitor with IC50 of 0.8, 1.2, and	Metabolis	C30H27BrN	free base	N/A		4,744	3	0	6

												http://selleckchem.com/products/lb42708.html	2.0 nM toward H-ras, N-ras, and K-ras, respectively.	m	4O2			BrC1=CC=C(CN2C=NC=C2CN2C=C(C(=O)N3CCOCC3)C(=C2)C2=C3C=CC=CC3=CC=C2)C=C1 |c:7,9,25,28,30,32,35,37,40,t:1,3,14|				
S7492	Uprosertib (GSK2141795)	43950663	c4	L3500-08	429,25	1047634-65-0	Akt	85	198	<1		http://selleckchem.com/products/gsk2141795.html	Uprosertib (GSK2141795) is a selective, ATP- competitive, and orally bioavailable Akt inhibitor with IC50 of 180 nM, 328 nM, and 38 nM for Akt 1, 2 and 3, respectively. Phase 2.	PI3K/Akt/mTOR	C18H16Cl2F2N4O2	free base	GSK795	CN1N=CC(Cl)=C1C1=C(Cl)OC(=C1)C(=O)N[C@H](CN)CC1=CC(F)=C(F)C=C1 |r,c:2,5,8,12,28,t:22,25|	3,531	3	2	6
S7497	CK-636	43460020	d4	L3500-08	284,38	442632-72-6	Microtubule Associated	57	200	<1		http://selleckchem.com/products/ck-636.html	CK-636 is an Arp2/3 complex inhibitor with IC50 of 4 μM, 24 μM and 32 μM for inhibition of actin polymerization induced by human, fission yeast and bovine Arp2/3 complex, respectively.	Cytoskeletal Signaling	C16H16N2OS	free base	CK-0944636	CC1=C(CCNC(=O)C2=CC=CS2)C2=CC=CC=C2N1 |c:1,10,16,18,t:8,14|	3,335	1	2	4
S7498	DDR1-IN-1	43468531	e4	L3500-08	552,59	1449685-96-4	Others	100	181	<1		http://selleckchem.com/products/ddr1-in-1.html	DDR1-IN-1 is a potent and selective discoidin domain receptor 1 (DDR1) receptor tyrosine kinase inhibitor with IC50 of 105 nM, about 3-fold selectivity over DDR2.	Others	C30H31F3N4O3	free base	N/A	CCN1CCN(CC2=CC=C(C=C2C(F)(F)F)C(=O)NC2=CC(OC3=CC=C4NC(=O)CC4=C3)=C(C)C=C2)CC1 |c:9,11,34,39,t:7,21,25,27,36|	4,972	3	2	8
S7500	HJC0350	43460788	f4	L3500-08	277,38	885434-70-8	cAMP	52	187	<1		http://selleckchem.com/products/hjc0350.html	HJC0350 is a potent and selective EPAC2 inhibitor with IC50 of 0.3 μM, exhibiting no inhibition on Epac1.	GPCR & G Protein	C15H19NO2S	free base	N/A	CC1=CN(C(C)=C1)S(=O)(=O)C1=C(C)C=C(C)C=C1C |c:5,11,17,t:1,14|	4,371	2	0	2
S7501	HO-3867	44093653	g4	L3500-08	464,55	1172133-28-6	STAT	13	28	<1		http://selleckchem.com/products/ho-3867.html	HO-3867, an analog of curcumin, is a selective STAT3 inhibitor that inhibits its phosphorylation, transcription, and DNA binding without affecting the expression of other active STATs.	JAK/STAT	C28H30F2N2O2	free base	N/A	[H]ON1C(C)(C)C=C(CN2C\C(=C/C3=CC=C(F)C=C3)C(=O)\C(C2)=C\C2=CC=C(F)C=C2)C1(C)C |c:18,32,t:6,13,15,27,29|	5,209	1	0	4
S7505	(S)-crizotinib	43959300	b5	L3500-08	450,34	1374356-45-2	MTH1	42	93	<1		http://selleckchem.com/products/s-crizotinib.html	(S)-crizotinib, the (S)- enantiomer of crizotinib, is a potent MTH1 (NUDT1) inhibitor with IC50 of 72 nM in a cell- free assay.	DNA Damage	C21H22Cl2FN5O	free base	N/A	C[C@H](OC1=C(N)N=CC(=C1)C1=CN(N=C1)C1CCNCC1)C1=C(Cl)C=CC(F)=C1Cl |r,c:3,6,8,14,24,27,30,t:11|	3,693	3	2	5
S7508	JNK Inhibitor IX	44118216	c5	L3500-08	332,42	312917-14-9	JNK	20	60	<1		http://selleckchem.com/products/jnk-inhibitor-ix.html	JNK inhibitor IX is a selective and potent JNK inhibitor with pIC50 of 6.5 and 6.7 for JNK2 and JNK3, respectively.	MAPK	C20H16N2OS	free base	TCS JNK 5a	O=C(NC1=C(C#N)C2=C(CCCC2)S1)C1=CC=CC2=C1C=CC=C2 |c:3,18,20,23,25,t:7,16|	4,571	1	1	2
S7513	Trelagliptin	44119968	d5	L3500-08	357,38	865759-25-7	DPP-4	71	199	<1			Trelagliptin is a highly selective,	Protease	C18H20FN5	free base	SYR-472	CN1C(=O)C=C(N2CCC[	1,476	2	1	3

												http://selleckchem.com/products/trelagliptin.html	long-acting DPP-4 inhibitor. Phase 3.	s	O2			C@@H](N) C2)N(CC2=CC(F)=CC=C 2C#N)C1= O |r,c:19,21,t:4,16|				
S7517	AZD7545	43451857	e5	L3500-08	478,87	252017-04-2	Others	95	198	<1		http://selleckchem.com/products/azd7545.html	AZD7545 is a potent PDHK inhibitor with IC50 of 36.8 nM and 6.4 nM for PDHK1 and PDHK2, respectively. It failed to inhibit PDHK4 at higher concentrations(>10 nM), AZD7545 stimulates PDHK4 activity.	Others	C19H18ClF3N2O5S	free base	N/A	CN(C)C(=O)C1=CC=C(C =C1)S(=O) (=O)C1=CC(Cl)=C(NC(= O)[C@@](C)(O)C(F)(F)F)C=C1 |r,c:7,9,30,t:5,15,18|	2,913	4	1	6
S7519	GNF-5837	44098068	f5	L3500-08	535,49	1033769-28-6	Trk receptor	100	187	<1		http://selleckchem.com/products/gnf-5837.html	GNF-5837 is a selective, and orally bioavailable pan-TRK inhibitor for TrkA, and TrkB with IC50 of 8 nM, and 12 nM, respectively.	Protein Tyrosine Kinase	C28H21F4N5O2	free base	N/A	CC1=CC=C(NC(=O)NC2 =CC(=CC= C2F)C(F)(F)F)C=C1NC1 =CC=C2C(NC(=O)\C2=C/C2=CC=C N2)=C1 |c:11,13,21,27,38,41,t:1,3, 9,25,36|	5,993	2	5	6
S7524	FR 180204	44084108	g5	L3500-08	327,34	865362-74-9	ERK	65	199	<1		http://selleckchem.com/products/fr180204.html	FR 180204 is an ATP-competitive, selective ERK inhibitor with Ki of 0.31 μM and 0.14 μM for ERK1 And ERK2, respectively. It is 30-fold less potent against the related kinase p38α and failed to inhibit any kinases(MEK1, MKK4, IKKα, PKCα, Src, Syc, and PDGFα) at less than 30 μM.	MAPK	C18H13N7	free base	N/A	NC1=NNC2=C1C=C(N=N 2)C1=C2C =CC=CN2N=C1C1=CC= CC=C1 |c:4,7,9,12,14,16,20,25,27 ,t:1,23|	2,969	4	2	2
S7525	XMD8-92	43892044	b6	L3500-08	474,55	1234480-50-2	ERK	73	154	<1		http://selleckchem.com/products/xmd8-92.html	XMD8-92 is a potent and selective BMK1/ERK5 inhibitor with Kd of 80 nM.	MAPK	C26H30N6O3	free base	N/A	CCOC1=CC(=CC=C1NC1 =NC2=C(C=N1)N(C)C(=O)C1=C(C =CC=C1) N2C)N1CCC(O)CC1 |c:5,7,13,15,24,26,t:3,11,2 2|	3,505	4	2	5
S7526	GNF-5	44121649	c6	L3500-08	418,37	778277-15-9	Bcr-Abl	83	198	<1		http://selleckchem.com/products/gnf-5.html	GNF-5 is a selective and allosteric Bcr-Abl inhibitor with IC50 of 220 nM.	Angiogenesis	C20H17F3N4O3	free base	N/A	OCCNC(=O)C1=CC=CC(=C1)C1=C C(NC2=CC=C(OC(F)(F)F)C=C2)=N C=N1 |c:8,10,26,28,30,t:6,13,17, 19|	4,876	4	3	8
S7528	GNE-7915	43471155	d6	L3500-08	443,4	1351761-44-8	LRRK2	22	50	<1		http://selleckchem.com/products/gne-7915.html	GNE-7915 is a highly potent, selective, and brain-penetrable leucine- rich repeat kinase 2 (LRRK2) inhibitor, with IC50 and Ki of 9 nM and 1 nM, respectively.	Autophagy	C19H21F4N5O3	free base	N/A	CCNC1=NC(NC2=CC(F) =C(C=C2O C)C(=O)N2CCOCC2)=N C=C1C(F)(F)F |c:10,12,25,27,t:3,7|	3,052	5	2	7
S7529	ML323	42518114	e6	L3500-08	384,48	1572414-83-5	DUB	76	198	<1		http://selleckchem.com/products/ml323.html	ML323 displays reversible, nanomolar inhibitory activity and excellent selectivity toward USP1/UAF1 with IC50 of 76 nM.	Ubiquitin	C23H24N6	free base	N/A	CC(C)C1=C(C=CC=C1) C1=NC(NC C2=CC=C(C=C2)N2C=C N=N2)=C(C)C=N1 |c:5,7,17,19,23,25,30,t:3,1 0,15,27|	5,042	4	1	6
S7531	UMI-77	43465416	f6	L3500-08	468,34	518303-20-3	Bcl-2	93	199	<1		http://selleckchem.com/products/umi-77.html	UMI-77 is a selective Mcl- 1 inhibitor with Ki of 490 nM, showing selectivity over other members of Bcl-2 family.	Apoptosis	C18H14BrNO5S2	free base	N/A	OC(=O)CSC1=C(O)C2=C C=CC=C2 C(NS(=O)(=O)C2=CC=C(Br)C=C2) |c:5,10,12,25,27,t:8,20,22|	3,755	3	2	6

S7534	BAPTA-AM	43651067	g6	L3500-08	764,68	126150-97-8	Others	20	26	<1		http://selleckchem.com/products/bapta-am.html	BAPTA-AM is a selective, membrane-permeable calcium chelator.	Others	C34H40N2O18	free base	N/A	CC(=O)OCOC(=O)CN(CC(=O)OCOC(C)=O)C1=C(OCCOC2=C(C=CC=C2)N(CC(=O)OCOC(C)=O)CC(=O)OCOC(C)=O)C=CC=C1 |c:19,27,29,51,53,t:25|	1,886	18	0	31
S7539	PTC-209 HBr	43452516	b7	L3500-08	576,1	1217022-63-3	BMI-1	100	174	<1		http://selleckchem.com/products/ptc-209-hbr.html	PTC-209 HBr is the hydrobromide salt of PTC-209, which is a potent and selective BMI- 1 inhibitor with IC50 of 0.5 µM, and results in irreversible reduction of cancer-initiating cells (CICs).	Cell Cycle	C17H14Br3N5OS	free base	N/A	Br.COC1=CC(Br)=C(NC2=NC(=CS2)C2=C(C)N=C3N=CC=CN23)C(Br)=C1 |c:10,14,19,21,27,t:2,5,8,17|	5,362	4	1	4
S7541	Decernotinib (VX-509)	43172612	c7	L3500-08	392,38	944842-54-0	JAK	78	199	<1		http://selleckchem.com/products/decernotinib-vx-509.html	Decernotinib (VX-509) is a potent and selective JAK3 inhibitor with Ki of 2.5 nM, >4-fold selectivity over JAK1, JAK2 and TYK2, respectively). Phase 2/3.	JAK/STAT	C18H19F3N6O	free base	N/A	CC[C@@](C)(NC1=NC(=NC=C1)C1=CNC2=NC=CC=C12)C(=O)NCC(F)(F)F |r,c:7,9,17,t:5,12,15,19|	2,747	4	3	7
S7555	4SC-202	43908221	d7	L3500-08	447,51	910462-43-0	HDAC	89	199	<1		http://selleckchem.com/products/4sc-202.html	4SC-202 is a selective class I HDAC inhibitor with IC50 of 1.20 µM, 1.12 µM, and 0.57 µM for HDAC1, HDAC2, and HDAC3, respectively. Also displays inhibitory activity against Lysine specific demethylase 1 (LSD1). Phase I.	Epigenetics	C23H21N5O3S	free base	N/A	CN1C=C(C=N1)C1=CC=C(C=C1)S(=O)(=O)N1C=CC(\C=C\C(=O)NC2=CC=CC=C2N)=C1 |c:2,4,9,11,18,28,30,33,t:7,26|	2,955	4	2	6
S7557	CL-387785 (EKI-785)	46340914	e7	L3500-08	381,23	194423-06-8	EGFR	63	165	<1		http://selleckchem.com/products/cl-387785-eki-785.html	CL-387785 (EKI-785) is an irreversible, and selective EGFR inhibitor with IC50 of 370 pM.	Protein Tyrosine Kinase	C18H13BrN4O	free base	N/A	CC#CC(=O)NC1=CC2=C(NC3=CC=CC(Br)=C3)N=CN=C2C=C1 |c:8,13,16,19,21,24,t:6,11|	4,568	3	2	4
S7566	IM-12	44197302	f7	L3500-08	377,41	1129669-05-1	GSK-3	75	199	<1		http://selleckchem.com/products/im-12.html	IM-12 is a selective GSK- 3β inhibitor with IC50 of 53 nM, and also enhances canonical Wnt signalling.	PI3K/Akt/mTOR	C22H20FN3O2	free base	N/A	CN1C(=O)C(NCCC2=CC=C(F)C=C2)=C(C1=O)C1=C(C)NC2=C1C=CC=C2 |c:13,15,20,24,27,29,t:8,10|	3,074	2	2	5
S7572	A-366	43464914	g7	L3500-08	329,44	1527503-11-2	Histone Methyltransferase	65	197	<1		http://selleckchem.com/products/a-366.html	A-366 is a potent and selective G9a/GLP histone lysine methyltransferase inhibitor with IC50 of 3.3nM, exhibiting >1000-fold selectivity for G9a/GLP over 21 other methyltransferases.	Epigenetics	C19H27N3O2	free base	N/A	COC1=C(OCCCN2CCCC2)C=C2N=C(N)C3(CCC3)C2=C1 |c:2,25,t:14,16|	2,467	3	1	6
S7573	GSK2830371	43439964	b8	L3500-08	461,02	1404456-53-6	phosphatase	92	200	<1		http://selleckchem.com/products/gsk2830371.html	GSK2830371 is an orally active, allosteric Wip1 phosphatase inhibitor with IC50 of 6 nM.	Others	C23H29ClN4O2S	free base	N/A	CC1=NC=C(Cl)C=C1NCC1=CC=C(S1)C(=O)N[C@@H](CC1CCCC1)C(=O)NC1CC1 |r,c:6,13,t:1,3,11|	4,064	3	3	9

S7574	GSK-LSD1 2HCl	44094082	c8	L3500-08	289,24	1431368-48-7(free base)	Histone Demethylase	57	197	57	197	http://selleckchem.com/products/gsk-lsd1-2hcl.html	GSK-LSD1 2HCl is an irreversible, and selective LSD1 inhibitor with IC50 of 16 nM, > 1000 fold selective over other closely related FAD utilizing enzymes (i.e. LSD2, MAO-A, MAO-B).	Epigenetics	C14H22Cl2N2	dihydrochloride	N/A	Cl.Cl.C1[C@@H](NC2CCNCC2)[C@@H]1C1=CC=CC=C1 |r,c:14,16,t:12|	1,964	0	2	3
S7577	AGK2	43438387	d8	L3500-08	434,27	304896-28-4	Sirtuin	10	23	<1		http://selleckchem.com/products/agk2.html	AGK2 is a potent, and selective SIRT2 inhibitor with IC50 of 3.5 µM that minimally affects either SIRT1 or SIRT3 at 10-fold higher levels.	Cytoskeletal Signaling	C23H13Cl2N3O2	free base	N/A	ClC1=CC=C(Cl)C(=C1)C1=CC=C(O1)\C=C(\C#N)C(=O)NC1=C2C=CC=NC2=CC=C1 |c:5,11,22,24,26,29,31,t:1,3,9|	5,641	3	1	4
S7585	SB-334867	43467556	e8	L3500-08	319,32	792173-99-0	OX Receptor	63	197	<1		http://selleckchem.com/products/sb-334867.html	SB-334867 is a selective orexin-1 (OX1) receptor antagonist.	GPCR & G Protein	C17H13N5O2	free base	N/A	CC1=NC2=C(O1)C=C(NC(=O)NC1=CC=NC3=CC=CN=C13)C=C2 |c:3,15,19,25,t:1,7,13,17,21|	1,773	5	2	2
S7589	N6022	43471491	f8	L3500-08	414,46	1208315-24-5	Others	82	198	<1		http://selleckchem.com/products/n6022.html	N6022 is a selective, and reversible inhibitor of S-nitrosoglutathione reductase (GSNOR) with IC50 of 8 nM and Ki of 2.5 nM. Phase 1/2.	Others	C24H22N4O3	free base	N/A	CC1=C(C=CC(=C1)C(N)=O)N1C(CCC(O)=O)=CC=C1C1=CC=C(C=C1)N1C=CN=C1 |c:3,5,17,19,24,26,30,32,t:1,22|	3,664	3	1	7
S7593	Splitomicin	44074208	g8	L3500-08	198,22	5690-03-9	HDAC	39	197	<1		http://selleckchem.com/products/splitomicin.html	Splitomicin is a selective NAD(+)-dependent histone deacetylase Sir2p inhibitor with IC50 of 60 µM, showing a higher activity in a cell- based assay.	Epigenetics	C13H10O2	free base	N/A	O=C1CCC2=C3C=CC=CC3=CC=C 2O1 |c:4,6,8,11,13|	2,831	2	0	0
S7595	Santacruzamate A (CAY10683)	43973505	b9	L3500-08	278,35	1477949-42-0	HDAC	55	198	<1		http://selleckchem.com/products/santacruzamate - a-cay10683.html	Santacruzamate A (CAY10683) is a potent and selective HDAC inhibitor with IC50 of 119 pM for HDAC2, >3600-fold selectivity over other HDACs.	DNA Damage	C15H22N2O3	free base	N/A	CCOC(=O)NCCCC(=O)NCCC1=CC=CC=C1 |c:16,18,t:14|	1,899	3	2	9
S7596	CAY10603	44172378	c9	L3500-08	446,5	1045792-66-2	HDAC	89	199	<1		http://selleckchem.com/products/cay10603.html	CAY10603 is a potent and selective HDAC6 inhibitor with IC50 of 2 pM, >200-fold selectivity over other HDACs.	DNA Damage	C22H30N4O6	free base	N/A	CC(C)(C)OC(=O)NC1=CC=C(C=C1)C1=CC(=NO1)C(=O)NCCCCCCC(=O)NO |c:10,12,17,t:8,15|	3,23	6	3	12
S7605	Filgotinib (GLPG0634)	44094736	d9	L3500-08	425,5	1206161-97-8	JAK	85	200	<1		http://selleckchem.com/products/filgotinib.html	Filgotinib (GLPG0634) is a selective JAK1 inhibitor with IC50 of 10 nM, 28 nM, 810 nM, and 116 nM for JAK1, JAK2, JAK3, and TYK2, respectively. Phase 2.	JAK/STAT	C21H23N5O3S	free base	N/A	O=C(NC1=NN2C(C=CC=C2C2=CC=C(CN3CCS(=O)(=O)CC3)C=C2)= N1)C1CC1 |c:7,9,26,28,t:3,12,14|	2,461	5	1	5
S7611	EI1	43887924	e9	L3500-08	390,48	1418308-27-6	Histone Methyltransferase	42	108	<1		http://selleckchem.com/p	EI1 is a potent and selective EZH2 inhibitor with IC50 of 15 nM and 13 nM for EZH2 (WT) and EZH2 (Y641F), respectively.	Epigenetics	C23H26N4O2	free base	N/A	CCC(CC)N1C=CC2=C(C=C(C=C12)C#N)C(=O)NCC1=C(C)C	3,54	2	2	6

												roducts/ei1.html						=C(C)NC1 =O |c:6,10,22,t:8,12,25|				
S7612	PX-478 2HCl	44198803	f9	L3500-08	394,12	685898-44-6	HIF	78	198	78	198	http://selleckchem.com/products/px-478-2hcl.html	PX-478 2HCl is an orally active, and selective hypoxia-inducible factor- 1α (HIF-1α) inhibitor. Phase 1.	Angiogenesis	C13H20Cl4N2O3	dihydrochloride	N/A	Cl.Cl.N[C@@H](CC1=CC=C(C=C1) [N+]([O-])(CCCl)CCCl)C(O)=O |r,c:5,7,t:3|	-0,251	1	1	8
S7620	GSK1324726A (I-BET726)	43091552	g9	L3500-08	434,91	1300031-52-0	Epigenetic Reader Domain	86	198	<1		http://selleckchem.com/products/gsk1324726a-i-bet726.html	GSK1324726A (I-BET726) is a highly selective inhibitor of BET family proteins with IC50 of 41 nM, 31 nM, and 22 nM for BRD2, BRD3, and BRD4, respectively.	Epigenetics	C25H23ClN2O3	free base	N/A	C[C@H]1C[C@@H](NC2=CC=C(Cl)C=C2)C2=C(C=CC(=C2)C2=CC=C(C=C2)C(O)=O)N1C(C)=O |r,c:10,15,17,22,24,t:5,7,13,20|	4,648	2	1	4
S7624	SD-208	42684102	b10	L3500-08	352,75	627536-09-8	TGF-beta/Smad	9	26	<1		http://selleckchem.com/products/sd-208.html	SD-208 is a selective TGF-βRI (ALK5) inhibitor with IC50 of 48 nM, >100- fold selectivity over TGF- βRII.	TGF-beta/Smad	C17H10ClFN6	free base	N/A	FC1=CC=C(Cl)C=C1C1=NC2=NC=CN=C2C(NC2=CC=NC=C2)=N1 |c:6,13,15,22,24,26,t:1,3,9,11,20|	3,139	5	1	3
S7625	Niraparib (MK-4827) tosylate	44141994	c10	L3500-08	492,59	1038915-73-9	PARP	98	199	<1		http://selleckchem.com/products/mk-4827-niraparib-tosylate.html	Niraparib (MK-4827) tosylate is a selective inhibitor of PARP1/PARP2 with IC50 of 3.8 nM/2.1 nM.	DNA Damage	C26H28N4O4S	4-?methylbenzen	arib tosylate, MK 4827 tos	CC1=CC=C(C=C1)S(O)(=O)=O.NC(=O)C1=CC=CC2=CN(N=C12)C1=CC=C(C=C1)[C@@H]1CCCNC1 |r,c:3,5,16,27,29,t:1,14,18,21,25|	4,267	2	2	4
S7631	TH287	43443491	d10	L3500-08	269,13	1609960-30-6	MTH1	53	197	<1		http://selleckchem.com/products/th287.html	TH287 is a potent and selective MTH1 (NUDT1) inhibitor with IC50 of 0.8 nM.	DNA Damage	C11H10Cl2N4	free base	N/A	CNC1=CC(=NC(N)=N1)C1=C(Cl)C(Cl)=CC=C1 |c:4,7,10,14,16,t:2|	3,449	2	2	2
S7632	TH588	43469282	e10	L3500-08	295,17	1609960-31-7	MTH1	20	68	<1		http://selleckchem.com/products/th588.html	selective MTH1 (NUDT1) inhibitor with IC50 of 5 nM. It has no relevant inhibition of any of the other tested proteins at 100?µM, although TH588 showed reasonable selectivity when tested on a much larger panel of 87 enzymes, GPCRs, kinases, ion channels and transporters at 10?µM.	DNA Damage	C13H12Cl2N4	free base	N/A	NC1=NC(=CC(NC2CC2)=N1)C1=C(Cl)C(Cl)=CC=C1 |c:3,10,13,17,19,t:1|	3,938	2	2	3
S7638	LDC1267	44178868	f10	L3500-08	560,55	1361030-48-9	TAM Receptor	100	178	<1		http://selleckchem.com/products/ldc1267.html	LDC1267 is a highly selective TAM kinase inhibitor with IC50 of <5 nM, 8 nM, and 29 nM for Mer, Tyro3, and Axl, respectively. Displays lower activity against Met, Aurora B, Lck, Src, and CDK8.	Protein Tyrosine Kinase	C30H26F2N4O5	free base	N/A	CCOC1=CN(N=C1C(=O)NC1=CC=C(OC2=CC=NC3=CC(OC)=C(OC)C=C23)C(F)=C1)C1=CC=C(F)C=C1C |c:6,19,34,42,t:3,12,14,17,21,25,29, 37,39|	6,213	7	1	9
S7651	SB225002	44089774	g10	L3500-08	352,14	182498-32-4	CXCR	70	199	<1		http://selleck	SB225002 is a potent, and selective CXCR2 antagonist with IC50 of 22 nM for inhibiting interleukin IL-8 binding to CXCR2, > 150-fold selectivity over the other 7-TMRs tested.	GPCR & G Protein	C13H10BrN3O4	free base	N/A	OC1=CC(=CC=C1NC(=O)NC1=C(B	2,923	1	3	3

												chem.com/products/sb225002.html						r)C=CC=C1)[N+]([O-])=O |c:3,5,12,15,17,t:1|				
S7655	CB-839	42600551	b11	L3500-08	571,57	1439399-58-2	Glutaminase	100	175	<1		http://selleckchem.com/products/cb-839.html	CB-839 is a potent, selective, and orally bioavailable glutaminase inhibitor with IC50 of 24 nM for recombinant human GAC. Phase 1.	Proteases	C26H24F3N7O3S	free base	N/A	FC(F)(F)OC1=CC(CC(=O)NC2=CC =C(CCCCC3=NN=C(NC(=O)CC4=C C=CC=N4)S3)N=N2)=CC =C1 |c:30,32,37,39,41,t:5,12,14,20,22,28|	5,597	8	2	13
S7656	CPI-360	44088850	c11	L3500-08	437,53	1802175-06-9	Histone Methyltransferase	20	46	<1		http://selleckchem.com/products/cpi-360.html	CPI-360 is a potent, selective,and SAM- competitive EZH1 inhibitor with IC50 of 102.3 nM, >100-fold selectivity over other methyltransferases.	Epigenetics	C25H31N3O4	free base	N/A	COC1=C(CNC(=O)C2=C(C)N([C@H](C)C3CCOCC3)C3=C2C=CC=C3)C(=O)NC(C)=C1 |r,c:2,8,21,24,26,33|	2,071	4	2	6
S7668	Picropodophyllin (PPP)	44127321	d11	L3500-08	414,41	477-47-4	IGF-1R	82	198	<1		http://selleckchem.com/products/picropodophyllin- ppp.html	Picropodophyllin (PPP) is a IGF-1R inhibitor with IC50 of 1 nM. It displays selectivity for IGF-1R and does not coinhibit tyrosine phosphorylation the IR, or of a selected panel of receptors less related to IGF-IR(FGF- R, PDGF-R, OR EGF- R).	Protein Tyrosine Kinase	C22H22O8	free base	AXL1717	[H][C@]12COC(=O)[C@@]1([H])[C@H](C1=CC(OC)=C(OC)C(OC)=C1)C1=CC3=C(OCO3)C=C1[C@@H]2 O |r,c:20,31,t:10,14,23,25|	2,111	7	1	4
S7674	Savolitinib(AZD6094, HMPL-504)	44050817	e11	L3500-08	345,36	1313725-88-0	c-Met	16	46	<1		http://selleckchem.com/products/hmpl-504-azd6094-volitinib.html	Savolitinib (volitinib, AZD6094, HMPL-504) is a novel, potent, and selective MET inhibitor currently in clinical development in various indications, including PRCC. The IC50 values of this compound for c- Met and p-Met are 5 nM and 3 nM, respectively. It shows exquisite selectivity for c-Met over 274 kinase.	Protein Tyrosine Kinase	C17H15N9	free base	Volitinib	C[C@H](N1N=NC2=NC=C(N=C12) C1=CN(C)N=C1)C1=CN2C=CN=C2 C=C1	1,032	6	0	3
S7680	SP2509	43462904	f11	L3500-08	437,9	1423715-09-6	Histone Demethylase	38	87	<1		http://selleckchem.com/products/sp2509.html	SP2509 is a selective histone demethylase LSD1 inhibitor with IC50 of 13?nM, showing no activity against MAO-A, MAO-B, lactate dehydrogenase and glucose oxidase.	Epigenetics	C19H20ClN3O5S	free base	HCI-2509	C\C(=N/NC(=O)C1=CC=CC(=C1)S(=O)(=O)N1CCOCC1)C1=CC(Cl)=C C=C1O |c:8,10,26,28,t:6,23|	1,891	5	2	5
S7683	PIK-III	44088304	g11	L3500-08	319,36	1383716-40-2	Autophagy,PI3K	63	197	<1		http://selleckchem.com/products/pik-iii.html	PIK-III, which is a selective inhibitor of ?VPS34 enzymatic activity, inhibits autophagy and de novo lipidation of LC3 and leads to the stabilization of autophagy substrates. The IC50 values for VPS34 and PI(3)Kδ are 0.018 μM and 1.2 μM respectively.	Autophagy	C17H17N7	free base	VPS34-IN2	NC1=NC=C(C(CC2CC2)=N1)C1=C C=NC(NC2=CC=NC=C2)=N1 |c:3,10,15,21,23,25,t:1,13,19|	2,2	5	2	5

Plate layout: L3500-01

	1	2	3	4	5	6	7	8	9	10	11	12
a	Empty	Empty	Empty	Empty	Empty	Empty	Empty	Empty	Empty	Empty	Empty	Empty
b	Empty	S1004<br>Veliparib (ABT-888)	S1029<br>Lenalidomide (CC-5013)	S1055<br>Enzastaurin (LY317615)	S1078<br>MK-2206 2HCl	S1092<br>KU-55933 (ATM Kinase Inhibitor)	S1106<br>OSU-03012 (AR-12)	S1129<br>SRT1720 HCl	S1154<br>SNS-314	S1180<br>XAV-939	S1196<br>Exemestane	Empty
c	Empty	S1007<br>Roxadustat (FG-4592)	S1033<br>Nilotinib (AMN-107)	S1065<br>Pictilisib (GDC-0941)	S1080<br>SU11274	S1093<br>GSK1904529A	S1109<br>BI 2536	S1130<br>YM155 (Sepantronium Bromide)	S1155<br>S3I-201	S1186<br>BIBR 1532	S1197<br>Finasteride	Empty
d	Empty	S1008<br>Selumetinib (AZD6244)	S1036<br>PD0325901	S1067<br>SB431542	S1085<br>Belinostat (PXD101)	S1094<br>PF-04217903	S1110<br>Varespladib (LY315920)	S1139<br>ADL5859 HCl	S1157<br>Delanzomib (CEP-18770)	S1188<br>Anastrozole	S1202<br>Dutasteride	Empty
e	Empty	S1014<br>Bosutinib (SKI-606)	S1041<br>STF-62247	S1069<br>Luminespib (AUY-922, NVP-AUY922)	S1087<br>Iniparib (BSI-201)	S1097<br>BTZ043 Racemate	S1113<br>GSK690693	S1141<br>Tanespimycin (17-AAG)	S1159<br>Ganetespib (STA-9090)	S1189<br>Aprepitant	S1210<br>Methotrexate	Empty
f	Empty	S1020<br>PD184352 (CI-1040)	S1044<br>Temsirolimus (CCI-779, NSC 683864)	S1070<br>PHA-665752	S1089<br>Refametinib (RDEA119, Bay 86-9766)	S1100<br>MLN8054	S1115<br>Odanacatib (MK-0822)	S1144<br>Ivacaftor (VX-770)	S1173<br>WZ4002	S1191<br>Fulvestrant	S1216<br>PFI-1 (PF-6405761)	Empty
g	Empty	S1022<br>Ridaforolimus (Deforolimus, MK-8669)	S1048<br>Tozasertib (VX-680, MK-0457)	S1071<br>HA14-1	S1091<br>Linsitinib (OSI-906)	S1103<br>ZM 447439	S1120<br>Everolimus (RAD001)	S1145<br>SNS-032 (BMS-387032)	S1174<br>Ostarine (GTx-024, MK-2866)	S1195<br>TAK-700 (Orteronel)	S1233<br>2-Methoxyestradiol (2-MeOE2)	Empty
h	Empty	Empty	Empty	Empty	Empty	Empty	Empty	Empty	Empty	Empty	Empty	Empty

Plate layout: L3500-03

	1	2	3	4	5	6	7	8	9	10	11	12
a	Empty	Empty	Empty	Empty	Empty	Empty	Empty	Empty	Empty	Empty	Empty	Empty
b	Empty	S1626<br>Naproxen Sodium	S1831<br>Carvedilol	S1913<br>Tropicamide	S2013<br>PF-573228	S2043<br>Memantine HCl	S2102<br>asagiline Mesyl	S2153<br>CGS 21680 HC	S2198<br>GI-1776 free ba	S2228<br>nacasan (VX-7	S2285<br>Cryptotanshinon	Empty
c	Empty	S1657<br>Enalaprilat Dihydrate	S1847<br>Clemastine Fumarate	S1959<br>Tolfenamic Acid	S2018<br>ENMD-2076 L-(+)-Tartaric acid	S2051<br>Captopril	S2103<br>Naltrexone HCl	S2163<br>PF-4708671	S2205<br>OSI-420	S2230<br>nisertib (LY2157	S2341<br>(-)-Parthenolide	Empty
d	Empty	S1738<br>Telmisartan	S1880<br>Roxatidine Acetate HCl	S2001<br>Elvitegravir (GS-9137, JTK-303)	S2020<br>Formoterol Hemifumarate	S2061<br>Lovastatin	S2104<br>Levosulpiride	S2168<br>PD128907 HCl	S2216<br>ubritinib (TAK 1	S2233<br>meprazole sodi	S2437<br>Rotundine	Empty
e	Empty	S1776<br>Toremifene Citrate	S1885<br>Felodipine	S2003<br>Maraviroc	S2026<br>Ginkgolide A	S2065<br>Lafutidine	S2109<br>Imidapril HCl	S2178<br>AG-14361	S2217<br>ecan HCl Trihy	S2239<br>Tubacin	S2449<br>Forskolin	Empty
f	Empty	S1801<br>Ranitidine Hydrochloride	S1905<br>Amlodipine	S2005<br>Raltegravir (MK-0518)	S2032<br>Rebamipide	S2077<br>rvastatin Calciu	S2149<br>GSK1292263	S2179<br>dotinib (LY2784	S2221<br>patinib?mesylat	S2262<br>Apigenin	S2454<br>Bupivacaine HC	Empty
g	Empty	S1802<br>AICAR (Acadesine)	S1909<br>Fluvastatin Sodium	S2006<br>Pyrimethamine	S2042<br>Cyproterone Acetate	S2085<br>Trimebutine	S2151<br>Erismodegib, NV	S2193<br>GSK461364	S2222<br>PF-3716556	S2270<br>Bergenin	S2496<br>Ozagrel	Empty
h	Empty	Empty	Empty	Empty	Empty	Empty	Empty	Empty	Empty	Empty	Empty	Empty

Plate layout: L3500-08

	1	2	3	4	5	6	7	8	9	10	11	12
a	Empty	Empty	Empty	Empty	Empty	Empty	Empty	Empty	Empty	Empty	Empty	Empty
b	Empty	S7414<br>Caffeic Acid Phenethyl Ester	S7445<br>E3330	S7467<br>LB42708	S7505<br>(S)-crizotinib	S7525<br>XMD8-92	S7539<br>PTC-209 HBr	S7573<br>GSK2830371	S7595<br>uzamate A (CA	S7624<br>SD-208	S7655<br>CB-839	Empty
c	Empty	S7421<br>CGP 57380	S7449<br>CRT0044876	S7492<br>Uprosertib (GSK2141795)	S7508<br>JNK Inhibitor IX	S7526<br>GNF-5	S7541<br>cernotinib (VX-5	S7574<br>GSK-LSD1 2HC	S7596<br>CAY10603	S7625<br>rib (MK-4827) t	S7656<br>CPI-360	Empty
d	Empty	S7424<br>PD 151746	S7457<br>XEN445	S7497<br>CK-636	S7513<br>Trelagliptin	S7528<br>GNE-7915	S7555<br>4SC-202	S7577<br>AGK2	S7605<br>otinib (GLPG06	S7631<br>TH287	S7668<br>ropodophyllin (P	Empty
e	Empty	S7430<br>SB-3CT	S7460<br>BTB06584	S7498<br>DDR1-IN-1	S7517<br>AZD7545	S7529<br>ML323	S7557<br>-387785 (EKI-7	S7585<br>SB-334867	S7611<br>EI1	S7632<br>TH588	S7674<br>b(AZD6094, HM	Empty
f	Empty	S7436<br>NH125	S7461<br>LDC000067	S7500<br>HJC0350	S7519<br>GNF-5837	S7531<br>UMI-77	S7566<br>IM-12	S7589<br>N6022	S7612<br>PX-478 2HCl	S7638<br>LDC1267	S7680<br>SP2509	Empty
g	Empty	S7437<br>Sal003	S7462<br>PI-1840	S7501<br>HO-3867	S7524<br>FR 180204	S7534<br>BAPTA-AM	S7572<br>A-366	S7593<br>Splitomicin	S7620<br>1324726A (I-BET	S7651<br>SB225002	S7683<br>PIK-III	Empty
h	Empty	Empty	Empty	Empty	Empty	Empty	Empty	Empty	Empty	Empty	Empty	Empty

Supplementary Table 4: Summary of a comparison of the differences between protocols that used commercially available or custom-made plates.

**Microwell plate, Cell type and numbers**	**Coating**	**Organoid type**	**EB**	**Cultured in**	**Embedding**	**Homogeneity**	**Duration (Days)**	**Cryo-preservation**	**Application and purpose**	**Ref.**
ULA 384-well plate;**hESCs**; 400, 2000 or 10000	Pluronic F-127	Embryoid bodies	yes	No transfer	yes	Consistent	4	no	None; High-throughput method to produce EBs	1
Custom agarose microwell;**hESCs and iPSCs**;5000-45000	no	Embryoid bodies	yes	Petri dishes	no	Consistent	20	no	None; High-throughput method to produce EBs	2
PDMS microwell arrays; **iPSCs**; N/A	no	Brain organoids	yes	6 well plate	yes	Variable	30	no	None; Simplified high-throughput method to produce brain organoids	3
3D printed PDMS; **hESCs**; 200000	mPEG, lipidure or BSA	Cerebral organoids	yes	T25 flask	no	Consistent	45	no	None; To produce uniform and mature cerebral organoids	4
eNUVIO EB-Disks;**iPSCs;**~1500	no	Midbrain organoids	yes	Spinner flask	yes	Consistent	100	no	None; High-throughput method to produce midbrain organoids	5
ULA 384-well plate;**iPSCs**; 1000	no	Cerebral organoids	yes	Pillar plates	yes	Variable	31	no	None; High-throughput and short method to produce cerebral organoids	6
AggreWell™800; **hESCs and iPSCs**; 2000	no	Cerebral organoids	yes	24 wells plate	yes	Variable	25	no	Compare human, gorilla and chimpanzee brain organoids; To investigate the human brain expansion mechanisms	7
AggreWell™400; **hESCs and iPSCs**; 1000	no	Midbrain organoids	yes	6 wells plate on orbital shaker	no	N/A	21	no	None; To produce dopaminergic neurons in midbrain organoids	8
AggreWell™800; **iPSCs**; 10000	no	Cortical organoids	no	Petri dishes	no	Consistent	100	no	None; High-throughput method to reliably produce cortical organoids	9
AggreWell™800; **iPSCs**; N/A	Rinsing Solution	Asteroids	no	96-wells plate	no	N/A	21	no	Drug screening in tauopathy; To model tauopathy and test a treatment	10
Custom-made COC plate; **iPSCs**;10000	no	Brain organoids	no	Spinner bioreactor	no	Consistent	150	yes	Modeling of microcephaly, glioma invasion, and drug screening; High-throughput and reproducible method to generate brain organoids	Current work

Supplementary Table 5 hiPSC details

Name	Donor species	Cell type	Karyotyp	Gender	Source
Crx-iPS	Homo sapiens sapiens	hiPSC	46, XX	female	Generated by Laboratory of Olivier Goureau and published in doi: 10.1155/2019/7858796[11]
TUBA1B-GFP	Homo sapiens sapiens	hiPSC	46, XY	male	Allen Cell Collection at Coriell, Cat. No.: AICS 0012
IMR90-1	Homo sapiens sapiens	hiPSC	46, XX	female	Commercially available at WiCell, ID: WISCi004-A iPS(IMR90) clone (#1)
TUBA1B-RFP	Homo sapiens sapiens	hiPSC	46, XY	male	Allen Cell Collection at Coriell, Cat. AICS-0031-035
CSB-GM739	Homo sapiens sapiens	hiPSC	46, XY	male	Generated by the Laboratory of Alyson Muotri
CDK5RAP2	Homo sapiens sapiens	hiPSC	46, XY	male	Reprogrammed in this work

All cell lines were maintained in mTeSR1 medium on Matrigel-coated tissue-culture dishes.

References:

1. Ungrin, M. D., Joshi, C., Nica, A., Bauwens, C. & Zandstra, P. W. Reproducible, ultra high-throughput formation of multicellular organization from single cell suspension-derived human embryonic stem cell aggregates. PloS one **3**, e1565 (2008). https://doi.org:10.1371/journal.pone.0001565
2. Pettinato, G., Wen, X. & Zhang, N. Formation of well-defined embryoid bodies from dissociated human induced pluripotent stem cells using microfabricated cell-repellent microwell arrays. Scientific reports **4**, 7402 (2014). https://doi.org:10.1038/srep07402
3. Zhu Y, Wang L, Yu H, et al. In situ generation of human brain organoids on a micropillar array. *Lab Chip*. 2017;17(17):2941-2950. doi:10.1039/c7lc00682a
4. Chen, Cheng et al. “A matrigel-free method to generate matured human cerebral organoids using 3D-Printed microwell arrays.” *Bioactive materials* vol. 6,4 1130-1139. 20 Oct. 2020, doi:10.1016/j.bioactmat.2020.10.003
5. Mohamed, N. V. et al. Microfabricated disk technology: Rapid scale up in midbrain organoid generation. Methods **203**, 465-477 (2022). https://doi.org:10.1016/j.ymeth.2021.07.008
6. Acharya, P. et al. Uniform cerebral organoid culture on a pillar plate by simple and reproducible spheroid transfer from an ultralow attachment well plate. Biofabrication **16** (2024). https://doi.org:10.1088/1758-5090/ad1b1e

7. Benito-Kwiecinski, S. et al. An early cell shape transition drives evolutionary expansion of the human forebrain. Cell **184**, 2084-2102.e2019 (2021). https://doi.org:10.1016/j.cell.2021.02.050
8. Tieng, V. et al. Engineering of midbrain organoids containing long-lived dopaminergic neurons. Stem cells and development **23**, 1535-1547 (2014). https://doi.org:10.1089/scd.2013.0442
9. Yoon, S. J. et al. Reliability of human cortical organoid generation. Nature methods **16**, 75-78 (2019). https://doi.org:10.1038/s41592-018-0255-0
10. Rickner, H. D. et al. Single cell transcriptomic profiling of a neuron-astrocyte assembloid tauopathy model. Nature communications **13**, 6275 (2022). https://doi.org:10.1038/s41467-022-34005-1

11. Slembrouck-Brec A. et al. Reprogramming of adult retinal müller glial cells into human-induced pluripotent stem cells as an efficient source of retinal cells." Stem cells international vol. **7858796**, (2019) doi:10.1155/2019/7858796
