# Supplementary material for: Reliability of high-quantity human brain organoids for modeling microcephaly, glioma invasion and drug screening
Source: Nat Commun. 2024 Dec 19;15:10703. doi: 10.1038/s41467-024-55226-6 (PMC11659410; doi:10.1038/s41467-024-55226-6)
Supplement: Supplementary file 3 — Description of Additional Supplementary Files [file 41467_2024_55226_MOESM3_ESM.pdf]

Description of Additional Supplementary Files

Supplementary Movie 1: Spinner flasks culturing Hi-Q brain organoids

Supplementary Movie 2: Day 20 organoids, PSD95

Supplementary Movie 3: Day 20 organoids, SOX2 and DCX

Supplementary Movie 4: Day 20 Synapsin

Supplementary Movie 5: Day 20 Acetylated alpha-tubulin and MAP2

Supplementary Movie 6: Day 20 PSD95

Supplementary Movie 7: Day 20 organoids, Tau and PCP4

Supplementary Movie 8: Day 60 organoids, Acetylated alpha-tubulin and MAP2

Supplementary Movie 9: Day 60 organoids, Actin and P-vimentin

Supplementary Movie 10: Day 60 organoids, Nestin and TUJ1

Supplementary Movie 11: Day 60 organoids, Pax6 and CTIP2

Supplementary Movie 12: Day 60 organoids, SOX2 and DCX

Supplementary Movie 13: Day 60 organoids, Synapsin

Supplementary Movie 14: Day 60 organoids, Tau and PCP4

Supplementary Movie 15: Control Day 30 never frozen organoids

Supplementary Movie 16: Post thaw Day 30 organoids. Example 1

Supplementary Movie 17: Post thaw Day 30 organoids. Example 2
