# Supplementary material for: Reliability of high-quantity human brain organoids for modeling microcephaly, glioma invasion and drug screening
Source: Nat Commun. 2024 Dec 19;15:10703. doi: 10.1038/s41467-024-55226-6 (PMC11659410; doi:10.1038/s41467-024-55226-6)
Supplement: Supplementary file 21 — Reporting Summary [file 41467_2024_55226_MOESM21_ESM.pdf]

nature portfolio

Corresponding author(s): *Double-anonymous peer review submissions: write DAPR and your manuscript number here instead of author names.*

Last updated by author(s): *YYYY-MM-DD*

Reporting Summary

Nature Portfolio wishes to improve the reproducibility of the work that we publish. This form provides structure for consistency and transparency in reporting. For further information on Nature Portfolio policies, see our Editorial Policies and the Editorial Policy Checklist.

Statistics

For all statistical analyses, confirm that the following items are present in the figure legend, table legend, main text, or Methods section.

n/a	Confirmed	
☐	☒	The exact sample size (*n*) for each experimental group/condition, given as a discrete number and unit of measurement
☐	☒	A statement on whether measurements were taken from distinct samples or whether the same sample was measured repeatedly
☐	☒	The statistical test(s) used AND whether they are one- or two-sided<br>*Only common tests should be described solely by name; describe more complex techniques in the Methods section.*
☐	☒	A description of all covariates tested
☒	☐	A description of any assumptions or corrections, such as tests of normality and adjustment for multiple comparisons
☐	☒	A full description of the statistical parameters including central tendency (e.g. means) or other basic estimates (e.g. regression coefficient) AND variation (e.g. standard deviation) or associated estimates of uncertainty (e.g. confidence intervals)
☐	☒	For null hypothesis testing, the test statistic (e.g. *F*, *t*, *r*) with confidence intervals, effect sizes, degrees of freedom and *P* value noted<br>*Give P values as exact values whenever suitable.*
☒	☐	For Bayesian analysis, information on the choice of priors and Markov chain Monte Carlo settings
☒	☐	For hierarchical and complex designs, identification of the appropriate level for tests and full reporting of outcomes
☒	☐	Estimates of effect sizes (e.g. Cohen's *d*, Pearson's *r*), indicating how they were calculated

Our web collection on statistics for biologists contains articles on many of the points above.

Software and code

Policy information about availability of computer code

Data collection	Custom codes have been developed for scRNA sequence analysis which are deposited.
Data analysis	Seurat (Ver.5) and Harmony (Ver 1.2) packages were used for scRNA seq analysis

For manuscripts utilizing custom algorithms or software that are central to the research but not yet described in published literature, software must be made available to editors and reviewers. We strongly encourage code deposition in a community repository (e.g. GitHub). See the Nature Portfolio guidelines for submitting code & software for further information.

Data

Policy information about availability of data

All manuscripts must include a data availability statement. This statement should provide the following information, where applicable:

- Accession codes, unique identifiers, or web links for publicly available datasets
- A description of any restrictions on data availability
- For clinical datasets or third party data, please ensure that the statement adheres to our policy

Raw data in this study have been deposited at https://www.ncbi.nlm.nih.gov/bioproject/PRJNA1091065

Research involving human participants, their data, or biological material

Policy information about studies with human participants or human data. See also policy information about sex, gender (identity/presentation), and sexual orientation and race, ethnicity and racism.

Reporting on sex and gender	Sex of the iPS cell donors are specified in the methods and table summarizing the cell lines used.
Reporting on race, ethnicity, or other socially relevant groupings	NA
Population characteristics	NA
Recruitment	NA
Ethics oversight	University of Jena, University of Dusseldorf and Istituto Superiore di Sanità, Rome

Note that full information on the approval of the study protocol must also be provided in the manuscript.

Field-specific reporting

Please select the one below that is the best fit for your research. If you are not sure, read the appropriate sections before making your selection.

☒ Life sciences ☐ Behavioural & social sciences ☐ Ecological, evolutionary & environmental sciences

For a reference copy of the document with all sections, see nature.com/documents/nr-reporting-summary-flat.pdf

Life sciences study design

All studies must disclose on these points even when the disclosure is negative.

Sample size	Sample sizes were chosen between controls and experiments to arrive statistical significance
Data exclusions	No data were excluded
Replication	Several independent replicates have been preformed to ensure the reproducibility. The number of replicates and batches performed are given in respective figure legends
Randomization	For organoid size measurements a random size was The phenotypeschosen from the bulk of organoids from independent batches.
Blinding	The phenotypes observed between healthy control and mutants were morphologically obvious and thus there was no blinded experiments were performed.

Reporting for specific materials, systems and methods

We require information from authors about some types of materials, experimental systems and methods used in many studies. Here, indicate whether each material, system or method listed is relevant to your study. If you are not sure if a list item applies to your research, read the appropriate section before selecting a response.

Materials & experimental systems

n/a	Involved in the study	
☐	☒	Antibodies
☐	☒	Eukaryotic cell lines
☒	☐	Palaeontology and archaeology
☐	☒	Animals and other organisms
☒	☐	Clinical data
☒	☐	Dual use research of concern
☒	☐	Plants

Methods

n/a	Involved in the study	
☒	☐	ChIP-seq
☒	☐	Flow cytometry
☒	☐	MRI-based neuroimaging

Antibodies

Antibodies used	Mouse anti-Nestin (1:100, Novus Biologicals), mouse anti-SOX2 (1:50, Abcam), rabbit anti-Arl13b (1:100, Proteintech), rabbit anti-Doublecortin (1:100, Synaptic Systems), rabbit anti-MAP2 (1:200, Proteintech), rabbit anti-synapsin-1 (1:200, Cell Signalling),Rabbit anti-TUJ1(1:400, Sigma -

Aldrich), Mouse anti-acetylated tubulin (1:400, Sigma-Aldrich), Mouse anti-Tau (1:100, DSHB), Rat anti-CTIP2 (1:300, Abcam), Rabbit anti-PCP4 (1:100, Proteintech), Rabbit anti-pH2AX (1:400, Cell signalling), Mouse anti-Actin (1:100, R&D systems), rabbit anti-PSD95 (1:100, Proteintech), mouse anti-PAX6 (1:100, Proteintech), mouse anti-p-vimentin (1:200, Abcam) and TUNEL staining kit (Thermo Fischer). We used Alexa Fluor Dyes conjugated with goat/donkey anti-mouse, anti-rabbit, or anti-rat (1:1000, molecular probes, Thermo Fisher, USA) for secondary antibodies. In addition, DAPI 1 µg/ml (Sigma Aldrich, USA) was used to stain the nucleus.

Validation

Validation statements for each antibody is provided in manufacturers declaration. Besides, the validation of antodies were cross checked in literatures.

Eukaryotic cell lines

Policy information about cell lines and Sex and Gender in Research

Cell line source(s)

Cell line sources are described in a tabular form in the manuscript.
Crx-iPS Homo
sapiens
sapiens
iPSCs 46, XX
female Generated by Laboratory of
Olivier Goureau and
published in
doi: 10.1155/2019/7858796.

TUBA1BGFP
Homo
sapiens
sapiens
iPSCs 46, XY male Allen Cell Collection at
Coriell, Cat. No.: AICS 0012.

IMR90-1 Homo
sapiens
sapiens
iPSCs 46, XX female Commercially available at
WiCell, ID: WISCi004-A
iPS(IMR90)

TUBA1BRFP
Homo
sapiens
sapiens
iPSCs 46, XY male Allen Cell Collection at
Coriell, Cat. AICS-0031-035
CSB-GM739 Homo
sapiens
sapiens
iPSCs 46, XY male Generated by the Laboratory
of Alyson Muotri
CDK5RAP2 Homo
sapiens
sapiens
iPSCs 46, XY

Authentication

Cell lines were authenticated for their pluripotency, differentiation, mycoplasma free, morphology and mutant verification by sequencing

Mycoplasma contamination

All lines were tested for mycoplasma free

Commonly misidentified lines
(See ICLAC register)

None of the misidentified cell lines were used in this study

Animals and other research organisms

Policy information about studies involving animals; ARRIVE guidelines recommended for reporting animal research, and Sex and Gender in Research

Laboratory animals

Immunosuppressed NOD1279
SCID mice

Wild animals	NA
Reporting on sex	NA
Field-collected samples	NA
Ethics oversight	Ethics Committee of the Istituto Superiore di Sanità, Rome (Pr. No. 4701/17).

Note that full information on the approval of the study protocol must also be provided in the manuscript.

Plants

Seed stocks	NA
Novel plant genotypes	NA
Authentication	NA
